# Adverse events caused by cannabinoids in middle aged and older adults for all indications: a meta-analysis of incidence rate difference

**DOI:** 10.1093/ageing/afae261

**Published:** 2024-11-27

**Authors:** Latha Velayudhan, Sara Pisani, Marta Dugonjic, Katie McGoohan, Sagnik Bhattacharyya

**Affiliations:** Division of Academic Psychiatry, Institute of Psychiatry, Psychology and Neuroscience, King’s College London, London SE5 8AF, UK; Division of Academic Psychiatry, Institute of Psychiatry, Psychology and Neuroscience, King’s College London, London SE5 8AF, UK; Division of Academic Psychiatry, Institute of Psychiatry, Psychology and Neuroscience, King’s College London, London SE5 8AF, UK; Division of Academic Psychiatry, Institute of Psychiatry, Psychology and Neuroscience, King’s College London, London SE5 8AF, UK; Division of Academic Psychiatry, Institute of Psychiatry, Psychology and Neuroscience, King’s College London, London SE5 8AF, UK

**Keywords:** cannabinoid-based medication, delta-9-tetrahydrocannabinol (THC), cannabidiol, adverse events, middle aged and older adults, systematic review, older people

## Abstract

**Background:**

Cannabinoid-based medicines (CBMs) are being used widely in older people. However, information on the incidence of adverse events (AEs) is limited.

**Objective:**

To quantify the incidence rate difference (IRD) of AEs in middle aged and older adults of age ≥50 years receiving CBMs and also examine associations with weekly doses.

**Design:**

Systematic review and meta-analysis.

**Data sources:**

MEDLINE, PubMed, EMBASE, CINAHL, PsychInfo, Cochrane Library and ClinicalTrials.gov (1st Jan 1990–12th June 2023).

**Methods:**

We included randomised clinical trials (RCTs) using CBMs with mean participant age ≥50 years for medicinal purposes for all clinical indications. Paired reviewers independently screened studies, extracted data and appraised risk of bias. We estimated pooled effect-sizes IRD under the random-effects model.

**Results:**

Data from 58 RCTs (37 moderate-high quality studies, pooled n = 6611, mean age range 50–87 years, 50% male, n = 3450 receiving CBMs) showed that compared with controls, the incidence of all-cause and treatment-related AEs attributable to delta-9-tetrahydrocannabinol (THC)-containing CBMs were: THC alone [IRD:18.83(95% Confidence Interval [CI], 1.47–55.79) and 16.35(95% CI, 1.25–48.56)] respectively; THC:cannabidiol (CBD) combination [IRD:19.37(95% CI, 4.24–45.47) and 11.36(95% CI, 2.55–26.48)] respectively. IRDs of serious AEs, withdrawals and deaths were not significantly greater for CBMs containing THC with or without CBD. THC dose-dependently increased the incidence of dry mouth, dizziness/lightheadedness, mobility/balance/coordination difficulties, dissociative/thinking/perception problems and somnolence/drowsiness. The interaction of weekly THC:CBD doses played a role in mostly neurological, psychiatric and cardiac side-effects.

**Conclusions:**

Although CBMs in general are safe and acceptable in middle aged and older adults, one needs to be mindful of certain common dose-dependent side-effects of THC-containing CBMs.

## Key Points

- There is a particular need to quantify risk of various adverse events (AEs) with use of cannabinoid-based medicines (CBMs) in older people.
- We examined incidence rate differences of AEs in middle aged and older adults receiving CBMs for all conditions.
- Delta-9-tetrahydrocannabinol (THC) containing CBMs were associated with gastrointestinal, neurological and psychiatric side-effects in a dose-related manner.
- Cumulative weekly doses of delta-9-THC and CBDs played a role in mostly neurological, psychiatric and cardiac side-effects.
- We present age-specific safety/tolerability information about cannabinoids that is critical to prescribing in older people.

## Introduction

Cannabinoid-based medicines (CBMs) are increasingly being used in the older people, a fast-growing segment of the population [1, 2]. The term cannabinoid generally refers to chemicals that have a certain (terpenophenolic) structure, which are naturally present in the extract of the cannabis plant (when they are also known as phytocannabinoids) or may have a synthetic origin. Out of 150 cannabinoids in the cannabis plant, delta-9-tetrahydorcannabinol (THC) and cannabidiol (CBD) are commonly used for medicinal purposes with a range of reported benefits [3–6].

For any novel treatment, safety and tolerability must be weighed up against clinical benefits to inform their use in different contexts. This is of particular importance in middle aged and older adults, who often have various comorbid health conditions requiring treatments that may interact with any additional treatment being prescribed. They are also more sensitive to side-effects of medications than many other demographic groups. With growing usage of CBMs, there is a particular need therefore to quantify the risk of various adverse events (AEs) associated with CBM use, so as to enable informed risk–benefit analysis during clinical use. However, to the best of our knowledge there is limited evidence in this regard. Although, a number of randomised clinical trials (RCTs) of CBMs have been carried out, the sample sizes of these RCTs on their own are underpowered to systematically and meaningfully estimate the risk of individual AEs. Against this background and in the absence of large-scale population level pharmaco-vigilance data which will only accrue over time, meta-analytic pooling of incidence rate data of individual AEs across placebo-controlled RCTs allows the best estimate of risk associated with CBM treatments based on available evidence. By estimating difference in the incidence rate between the CBM and control intervention arms, such evidence can help understand the additional risk of AEs associated with CBM use. A number of previous reviews [7–10] have examined whether CBMs are associated with greater risk (either as odds or risk ratios or incident rate ratios) of side-effects and reported them as ratios. However, estimates of relative effect such as these do not lend themselves as easily to use in a clinical context unless the risk in the control group is readily known. Incident rate difference (IRD), which in this context refers to the additional risk of AEs estimated as the number of events per person-years of exposure associated with CBM use over and above a control intervention may be more easily understood but has not been systematically examined before. Another gap in current evidence relates to understanding about how the risk of AEs relate to the range of doses or ratio of doses used in formulations containing single or multiple cannabinoids respectively being used in the clinical settings. With limited number of studies being available, there is a paucity of data for any clinical indication-specific dose–response relationships with regard to AEs (that may exist) to become easily apparent. Meta-analytic pooling of data therefore will allow for an estimation of the likelihood of such risks at different doses across clinical indications to inform clinicians and researchers. Such a detailed assessment may help inform use of CBM in older people, in whom certain side-effects may be more directly related to morbidity and even mortality. For example, dizziness, which may contribute to risk for falls in older people, can in turn result in serious injuries such as fractures, head injuries or accidental deaths [11, 12]. Furthermore, certain AEs and dose–response relationships may be systematically different between THC and THC:CBD formulations [8]. Therefore, the overarching objective of the present endeavour was to address these gaps in knowledge by conducting a search of evidence from placebo-controlled RCTs and systematically report the incidence rate of all individual AEs attributable to the use of different types of CBMs. Specifically, we aimed to quantify the IRDs of AEs in people receiving THC only and THC-CBD combination treatment in middle aged and older adults with mean age of 50 years and older. We also aimed to examine the association of AEs with the weekly doses of THC and CBD.

## Methods

### Search strategy and selection criteria

The review was undertaken according to the Preferred Reporting Items for Systematic Reviews and Meta-analyses (PRISMA) reporting guidelines [13] and registered with PROSPERO (CRD42019148869). A detailed description of the bibliographic search strategy, as previously published, is presented in Supplementary Methods [8]. We identified studies published from 1 January 1990 up to 12 June 2023, from several electronic databases. Studies were independently assessed by pair of researchers (LV, KM, SP, MD) and disagreements resolved through consensus or discussions with senior researcher.

As described in our previous meta-analysis [8], studies were included if [1] published from 1990 onwards; [2] included middle aged and older adults (defined as mean age ≥50 years) or reported a distinct subgroup of middle aged and older adults and provided separate results for this subgroup; and [3] provided data on the safety and tolerability of medical cannabinoids administered by any route, at any dose, for any duration and for any indication. Studies were excluded if they [1] included exclusively younger subjects (≤50 years); [2] studied effects of cannabinoids for recreational purposes or failed to provide the dosage of cannabinoids and [3] were not reported in English language. Here we focus on results from RCTs.

### Data analysis

All relevant available data for examination of the safety and tolerability of different CBMs (THC:CBD combination or THC alone) was collected from eligible studies, complemented with information from www.ClinicalTrials.gov and we also contacted study authors to supplement information. Data were extracted for study design, participant characteristics, indication, dosage and duration of intervention, all cause and treatment-related AEs and serious AEs (SAEs), AE-related withdrawals and deaths. AEs and SAEs were coded according to the Medical Dictionary for Regulatory Activities (MedDRA) ‘system organ classes’ (SOC). Data were extracted for the top five (as reported by each study) AEs for each SOC, where available. Withdrawals and deaths outcomes were extracted as reported in the studies from the text and tables for each treatment arm. Data extraction and coding was verified by a medically qualified researcher and discrepancies resolved following discussions with senior researcher. The disease conditions investigated were classified into broader subgroups for analysis purpose [8]. Overall quality of evidence was assessed using recommended criteria [14] and summarised to reflect confidence in estimates [15].

Pooled effect-sizes were estimated (as square root transformed incident rate difference; IRSD) if there were two or more RCTs within each group or sub-group under the random-effects model using the restricted maximum-likelihood estimator because of anticipated heterogeneity. For reporting purposes, IRSDs have been converted to IRDs for ease of understanding, unless otherwise specified. However, for dose–response relationships (as in Tables 2 and 4), we have reported the IRSD values to allow an interested reader to estimate the expected IRD for a particular dose of CBM and shown an example calculation in table footnotes. Doses of both THC and CBD were included separately, as well as their interaction as predictors, in the same regression model for studies using THC:CBD combinations (Table 4). For each category of intervention, analyses combined both parallel-arm and crossover RCTs, with the latter treated as parallel-arm design [16] for pooled analyses. We also report results by RCT design. In studies with more than one active treatment arm, each active arm was considered as a different study. Throughout the manuscript, results are reported for analyses treating all studies as independent. We investigated heterogeneity using forest-plots and the *QE* statistic (and its significance; *QEp*) and publication bias using Egger’s regression test [17] and the ‘Trim and fill’ method [18]. Data for all clinical conditions were combined. We also examined the effect of treatment, design, clinical condition and weekly dose of THC and CBD and their interaction in THC:CBD combination studies using meta-regression except for the route of administration which was oral for all the included studies. Statistical analyses were performed using the metafor package in R (version 3.6.3) [19].

## Results

**Figure 1:**
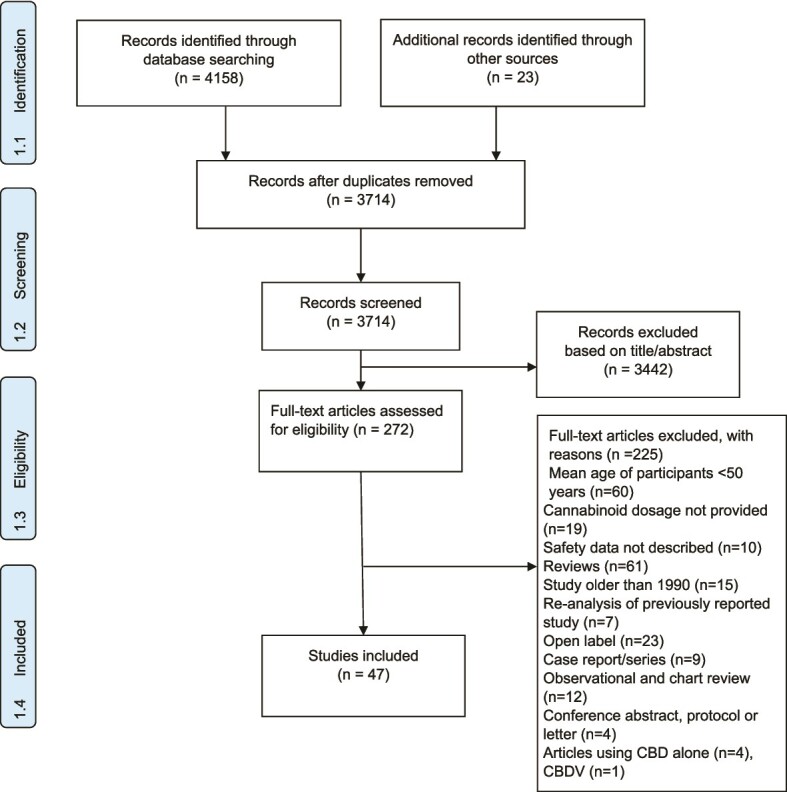
PRISMA flow diagram. In total, 58 studies were obtained from 47 papers, as in studies with more than one active treatment arm, each active arm was considered as a different study.

A total of 58 RCTs (n = 6611 participants; 1655.84 person-years of cannabinoid exposure) from 47 published articles were included (see Fig. 1, PRISMA flow chart for summary of study selection procedure and Supplementary Table 1a-b in the Supplementary Material for main study characteristics).

Supplementary Figures 1-7 (THC studies) and Figs 8–14 (THC: CBD combination studies) show the forest-plots and results stratified according to study design, for all cause and treatment-related AEs and SAEs, withdrawals, deaths, respectively.

Overall study quality (Grading of Recommendations Assessment, Development and Evaluation, GRADE) [15] is reported in Supplementary Table 1a and 1b. Risk of bias estimates are reported in Supplementary Figs 15(a,b) and 16(a,b). Sub-group meta-analysis at SOC level was done for systems with three or more AEs for THC (Supplementary Table 2a) and THC-CBD combination treatment (Supplementary Table 2b).

### THC studies

In total, 31 RCTs (15 crossover and 16 parallel-arm) from 29 articles [20–48] (see Supplementary Table 1a in the Supplementary Material), reported on 1473 patients (analysed 1429; 1255.82 person-years; mean ± SD: 40.51 ± 181.32 person-years) on active and 1265 (analysed 1224) on control intervention, with mean reported ages across studies ranging from 50–87 years (males: 0–100%). All except four studies used placebo as control [20, 23, 32, 43].

Pooled IRDs for all cause (*k* = 21) and treatment-related AEs (*k* = 9) from all RCTs were 18.83 (95% Confidence Interval [CI], 1.47–55.79) and 16.35 (95% CI, 1.25–48.56) AEs per 1000 person-years, respectively. Pooled IRDs of the most commonly reported AEs (Table 1) suggested significantly higher incidence rate of dizziness/lightheadedness, somnolence/drowsiness, impaired mobility/balance/coordination, sedation, headache, dissociative/thinking/perception disorders, euphoria and dry mouth, amounting on average to an additional incidence of 0.819 (95% CI 0.489–1.232), 0.684 (95% CI 0.055–2.014), 0.078 (95% CI 0.006–0.234), 11.103 (95% CI 0.596–34.721), 5.287 (95% CI 0.191–17.324), 0.510 (95% CI 0.260–0.844), 9.117 (95% CI 0.765–26.669) and 1.059 (95% CI 0.346–2.161) per 1000 person-years respectively in active compared to control arms.

**Table 1:** Effect of cumulative THC treatment across studies expressed as incidence rate difference (IRD, indicated by the summary estimate) followed by 95% confidence intervals and associated *P* value for each type of adverse event.

MedDRA high-level grouping	Individual AE	Summary estimate	95% CI (lower, upper)	*P* value	N	Q	Qp
Blood/Lymphatic System	Anaemia	0.002	0.162, 0.230	0.863	16	2.105	1.000
Cardiac	Dyspnoea	0.002	0.159, 0.233	0.852	16	1.329	1.000
	Palpitation	0.000	0.000, 0.000	0.992	15	1.436	1.000
	Chest pain	0.000	0.219, 0.187	0.938	16	3.720	0.999
Ear & Labyrinth	Vertigo	0.000	0.185, 0.222	0.929	15	3.992	0.996
Eye Disorders	Visual impairment/disturbances	0.141	0.006, 0.681	0.103	15	1.201	1.000
**Gastrointestinal**	Nausea	0.021	0.082, 0.330	0.511	22	29.595	0.100
	Vomiting	0.040	0.047, 0.378	0.348	18	10.998	0.857
	**Dry Mouth**	**1.059**	**0.346, 2.161**	**<0.001**	**20**	**43.062**	**0.001**
General	Pain: non-specific	0.000	0.178, 0.209	0.937	17	3.552	0.999
	Fatigue/tiredness	0.003	0.024, 0.067	0.615	21	4.958	1.000
	Weakness/reduced mobility	0.009	0.125, 0.299	0.674	16	2.911	1.000
Infections	Unspecified	0.001	0.030, 0.058	0.754	16	2.738	1.000
	UTI	0.009	0.013, 0.091	0.378	16	0.210	1.000
	RTI	0.001	0.178, 0.226	0.908	16	14.231	0.508
Injury/Poisoning	Falls & injuries	0.001	0.029, 0.060	0.723	15	2.330	1.000
Investigations	Raised Gamma GT	0.001	0.171, 0.232	0.881	16	3.370	0.999
Metabolism/Nutritional	Fluid retention	0.001	0.157, 0.196	0.913	16	2.129	1.000
	Decreased appetite	0.001	0.169, 0.232	0.877	16	5.407	0.988
	Increased appetite	0.001	0.182, 0.223	0.920	16	1.990	1.000
Musculoskeletal	Spasm stiffness	0.000	0.035, 0.052	0.852	17	1.998	1.000
	Joint disorders	0.005	0.019, 0.077	0.513	16	0.116	1.000
	Musculoskeletal pain	0.039	0.000, 0.163,	0.062	18	13.544	0.699
**Nervous System**	**Sedation**	**11.103**	**0.596, 34.721**	**0.011**	2	1.855	0.173
	**Dizziness/Lightheaded**	**0.819**	**0.489, 1.232**	**<0.001**	**25**	**61.099**	**<0.001**
	**Mobility/Balance/Coordination**	**0.078**	**0.006, 0.234**	**0.007**	17	21.216	0.170
	Muscle weakness	0.006	0.016, 0.082	0.453	18	5.052	0.998
	**Headache/migraine**	**5.287**	**0.191, 17.324**	**0.016**	8	10.475	0.163
**Psychiatric**	Sleep problems/Insomnia	0.110	0.158, 1.125	0.373	17	9.656	0.884
	**Dissociative/Thinking/Perception**	**0.510**	**0.260, 0.844**	**<0.001**	17	17.454	0.357
	**Somnolence/Drowsiness**	**0.684**	**0.055, 2.014**	**0.006**	**20**	**53.934**	**<0.001**
	Anxiety/Depression	0.008	0.014, 0.089	0.399	13	2.708	0.997
	Concentration/attention problem	6.361	0.051, 27.778	0.072	5	2.146	0.709
	**Euphoria**	**9.117**	**0.765, 26.669**	**0.006**	6	3.171	0.674
Renal and Urinary	Bladder symptoms	0.009	0.128, 0.295	0.686	15	0.020	1.000
Reproductive system	Male impotence	0.058	0.438, 0.032	0.260	15	8.150	0.881
Respiratory/Thoracic	Nose tenderness	0.001	0.178, 0.229	0.902	15	1.985	1.000
Skin/Subcutaneous	Other skin problem	0.072	0.515, 0.033	0.244	15	3.397	0.998
	Rash	0.002	0.161, 0.249	0.831	15	1.968	1.000
	Pressure sore	0.108	0.606, 0.015	0.153	15	1.225	1.000
Vascular	Hypotension	0.000	0.195, 0.205	0.980	16	3.999	0.998

I^2^ = percent of total variability (heterogeneity plus sampling variability) attributed to heterogeneity amongst the true effects. N = number of studies included in analysis. QE = test statistic for the test of heterogeneity. QEp = *P* value for the test of heterogeneity. UTI, Urinary tract infection. RTI, Respiratory tract infection. NA = not applicable. Statistically significant results are presented in bold.

Meta-regression analyses suggested a significant association between cumulative THC dose per week across THC studies and incidence rate (expressed as IRSD) of some of the AEs (Table 2) such as dry mouth, dizziness/lightheadedness, mobility/balance/coordination difficulties, dissociative/thinking perception and somnolence/drowsiness. However, these estimates need to be interpreted with caution due to heterogeneity across the studies reporting the AEs.

**Table 2:** Effect of cumulative THC dose per week across THC studies expressed as square root transformed incidence rate difference (IRSD, i.e. summary estimate) for each single adverse event. Summary estimates are reported here for intercept and THC dose per week (i.e. regression coefficient), followed by 95% confidence intervals and associated *P* value.

MedDRA high-level grouping	Individual AE	MODEL	Summary estimate	95% CI (lower, upper)	*P* value	N	QE	QEp
**Gastrointestinal**	Nausea	Intercept	0.013	−0.010, 0.036	0.268	22	28.800	0.092
		THC	0.000	−0.000, 0.000	0.373	22		
	Vomiting	Intercept	0.016	−0.005, 0.037	0.139	18	9.686	0.883
		THC	0.000	0.000, 0.000	0.252	18		
	**Dry mouth**	Intercept	0.075	0.050, 0.100	<0.001	20	27.136	0.076
		**THC**	**−0.00005**	**−0.00001, 0.00003**	**<0.001**	20		
**Nervous System**	**Dizziness/Lightheaded**	Intercept	0.055	0.038, 0.071	<0.001	25	**49.357**	**0.001**
		**THC**	**−0.00001**	**−0.00003, −0.00001**	**0.001**	**25**		
	**Mobility/Balance/Coordination**	Intercept	0.025	0.009, 0.041	0.002	17	16.338	0.360
		**THC**	**−0.000005**	**−0.00001, −0.000001**	**0.027**	17		
	Muscle weakness	Intercept	0.003	−0.015, 0.020	0.744	18	5.049	0.996
		THC	0.000	0.000, 0.000	0.961	18		
	Headache/migraine	Intercept	0.004	−0.095, 0.104	0.936	8	7.667	0.264
		THC	0.002	0.000, 0.004	0.094	8		
	Sedation	Intercept	0.260	0.023, 0.498	0.032	2	0.000	1.000
		THC	−0.013	−0.032, 0.006	0.173	2		
**Psychiatric**	Sleep problems/Insomnia	Intercept	0.039	−0.024, 0.102	0.222	16	8.723	0.848
		THC	0.000	−0.001, 0.000	0.338	16		
	**Dissociative/Thinking/Perception**	Intercept	0.002	−0.013, 0.018	0.761	17	9.988	0.820
		**THC**	**0.00001**	**0.000002, 0.00001**	**0.006**	17		
	**Somnolence/Drowsiness**	Intercept	0.064	0.030, 0.097	<0.001	20	**47.142**	**<0.001**
		**THC**	**−0.0003**	**−0.00001, −0.001**	**0.009**	**20**		
	Anxiety/Depression	Intercept	−0.002	−0.020, 0.016	0.824	13	2.377	0.997
		THC	0.000	0.000, 0.000	0.565	13		
	Concentration/attention problem	Intercept	0.119	0.006, 0.232	0.039	5	1.004	0.800
		THC	−0.001	−0.004, 0.001	0.285	5		
	Euphoria	Intercept	0.098	−0.030, 0.226	0.135	6	3.169	0.530
		THC	0.000	−0.003, 0.002	0.967	6		
Cardiac	Dyspnoea	Intercept	0.004	−0.021, 0.029	0.744	16	1.256	1.000
		THC	0.000	0.000, 0.000	0.788	16		
	Palpitation	Intercept	0.000	−0.001, 0.001	0.973	15	1.434	1.000
		THC	0.000	0.000, 0.000	0.968	15		
	Chest pain	Intercept	−0.002	−0.029, 0.025	0.886	16	3.706	0.997
		THC	0.000	0.000, 0.000	0.904	16		
Vascular	Hypotension	Intercept	0.001	−0.026, 0.027	0.963	16	3.998	0.995
		THC	0.000	0.000, 0.000	0.969	16		
Infections	Unspecified	Intercept	−0.006	−0.024, 0.011	0.470	16	2.312	1.000
		THC	0.000	0.000, 0.000	0.514	16		
	UTI	Intercept	0.001	−0.017, 0.018	0.929	16	0.006	1.000
		THC	0.000	0.000, 0.000	0.651	16		
	RTI	Intercept	0.008	−0.018, 0.035	0.534	15	7.858	0.853
		THC	0.000	0.000, 0.000	0.601	15		
General	Pain: non-specific	Intercept	−0.009	−0.034, 0.016	0.490	16	2.554	1.000
		THC	0.000	0.000, 0.000	0.416	16		
	Fatigue/tiredness	Intercept	0.005	−0.012, 0.022	0.584	21	4.812	1.000
		THC	0.000	0.000, 0.000	0.702	21		1.000
	Weakness/reduced mobility	Intercept	0.015	−0.012, 0.042	0.271	16	0.451	1.000
		THC	0.000	0.000, 0.000	0.117	16		
Blood/Lymphatic System	Anaemia	Intercept	0.004	−0.021, 0.029	0.761	16	2.042	1.000
		THC	0.000	0.000, 0.000	0.802	16		
Ear & Labyrinth	Vertigo	Intercept	0.002	−0.024, 0.029	0.871	15	3.974	0.991
		THC	0.000	0.000, 0.000	0.892	15		
Eye Disorders	Visual impairment/disturbances	Intercept	0.010	−0.016, 0.037	0.451	15	1.182	1.000
		THC	0.000	0.000, 0.000	0.891	15		
Injury/Poisoning	Falls & injuries	Intercept	0.007	−0.011, 0.025	0.436	14	1.845	1.000
		THC	0.000	0.000, 0.000	0.486	14		
Investigations	Raised gamma GT	Intercept	0.004	−0.023, 0.030	0.792	16	3.322	0.998
		THC	0.000	0.000, 0.000	0.828	16		
Metabolism/Nutritional	Fluid retention	Intercept	−0.002	−0.024, 0.020	0.864	16	2.112	1.000
		THC	0.000	0.000, 0.000	0.895	16		
	Decreased appetite	Intercept	0.003	−0.023, 0.029	0.801	16	5.367	0.980
		THC	0.000	0.000, 0.000	0.841	16		
	Increased appetite	Intercept	0.002	−0.024, 0.029	0.863	16	1.970	1.000
		THC	0.000	0.000, 0.000	0.888	16		
Musculoskeletal	Spasm stiffness	Intercept	0.001	−0.016, 0.019	0.894	17	1.993	1.000
		THC	0.000	0.000, 0.000	0.945	17		
	Joint disorders	Intercept	0.001	−0.017, 0.018	0.947	16	0.003	1.000
		THC	0.000	0.000, 0.000	0.737	16		
	Musculoskeletal pain	Intercept	0.007	−0.010, 0.025	0.396	18	10.692	0.828
		THC	0.000	0.000, 0.000	0.091	18		
Reproductive system	Male impotence	Intercept	−0.020	−0.042, 0.002	0.072	15	6.188	0.939
		THC	0.000	0.000, 0.000	0.161	15		0.939
Respiratory/Thoracic	Nose tenderness	Intercept	0.003	−0.024, 0.030	0.822	15	1.949	1.000
		THC	0.000	0.000, 0.000	0.850	15		
Skin/Subcutaneous	Other skin problem	Intercept	0.005	−0.021, 0.032	0.694	15	1.961	1.000
		THC	0.000	0.000, 0.000	0.231	15		
	Rash	Intercept	0.005	−0.021, 0.032	0.692	15	1.856	1.000
		THC	0.000	0.000, 0.000	0.738	15		
	Pressure sore	Intercept	0.002	−0.024, 0.029	0.864	15	0.007	1.000
		THC	0.000	0.000, 0.000	0.270	15		
Renal and Urinary	Bladder symptoms	Intercept	0.001	−0.025, 0.028	0.915	15	0.004	1.000
		THC	0.000	0.000, 0.000	0.898	15		

N = number of studies included in analysis. QE = test statistic for the test of heterogeneity. QEp = *P* value for the test of heterogeneity. NA = not applicable. Statistically significant results are presented in bold.

A summary estimate of the intercept model represents the square root transformed incidence rate difference (IRSD) of a given AE when the dose of THC treatment (per week) is 0. The summary estimate for THC refers to the additional increase in incidence rate per milligramme of increase in weekly THC dose per person-year, over and above the corresponding summary estimate of the intercept. Reported here need to be converted into incidence rate difference to be meaningfully interpreted as shown in the example below. For example, the square root transformed incident rate difference of developing dizziness/lightheadedness for a person taking 100 mg of THC per week over 1 year may be estimated using the formula [IRSD = intercept + summary estimate * (THC dose per week)] as IRSD = 0.055 + (−0.00001)*100,= 0.056. To convert IRSD per person-year into incidence rate difference (IRD) per 1000 person-years (i.e. incidence rate difference associated with cumulative exposure at the specified dose over 1 year for 1000 individuals) one would need to use the formula (IRSD^2^ * 1000). Using this formula, the additional incidence (IRD) of dizziness/lightheadedness attributable to THC exposure of 100 mg/week in 1000 people over 1 year amounts to 3.136 per 1000 person-years. Therefore, in a sample of 1000 individuals taking100 mg of THC per week over a 1-year period, 3.136 additional individuals will experience dizziness/lightheadedness attributable to their THC treatment.

Pooled IRDs for all cause (*k* = 28) and treatment-related (*k* = 24) SAEs from all RCTs were 0.002 (95% CI, 0.117–0.188) and 0.908 (95% CI, 0.05–4.54) SAEs per 1000 person years respectively. Pooled IRDs for all cause (*k* = 14) and treatment-related withdrawals (*k* = 28) from all RCTs were 0.052 (95% CI, 0.43–0.04) and 0.517 (95% CI, 0.01–2.34) withdrawals per 1000 person years respectively. IRDs for all cause deaths (*k* = 31) from all RCTs were 0.023 (95% CI, 0.002–0.012) deaths per 1000 person-years.

Neither Egger’s test nor ‘Trim and fill’ method indicated publication or other selection bias for any of the other outcomes except for all cause AEs (Supplementary Figs 17a-f, 18a-f, 19a-e, 20a-c). For all cause AEs for all RCTs as outcome, Egger’s test was p = 0.0265, and Trim and fill method indicated one missing study. Meta-regression analyses indicated effects of clinical condition on estimated effect of THC treatment on all cause AEs, which seemed to be mainly related to a significantly lower estimated effect in crossover studies investigating neurodegenerative disorder (p = 0.005) patients compared to other conditions.

### THC: CBD combination

A total of 27 studies (five crossover and 22 were parallel-arm; see Supplementary Table 1b in the Supplementary Material for additional details) from 22 articles [24, 26, 27, 31, 42, 49–64] reported on 1977 patients (analysed 1952; 400.02 person-years; mean ± SD: 14.82 ± 27.71 person-years) on active and 1896 (analysed 1872) on placebo, with mean reported ages across studies ranging from 51 to 67 years (males: 0–80%). All studies used placebo as control.

Pooled IRDs for all cause (*k* = 16) and treatment-related (*k* = 10) AEs from all RCTs was 19.37 (95% CI, 4.24–45.47) and 11.36 (95% CI, 2.55–26.48) respectively. Pooled IRDs of AEs (Table 3) from cumulative THC: CBD combination treatment across all studies per 1000 person-years for each single AE suggested significantly higher incidence rate of nausea, vomiting, dry mouth, fatigue/tiredness, dizziness/lightheadedness, somnolence/drowsiness and disorientation, amounting on average to an additional incidence of 0.674 (95% CI 0.100–1.7 54), 0.214 (95% CI 0.000–0.837), 1.227 (95% CI 0.093–3.650), 0.439 (95% CI 0.025–1.361), 2.467 (95% CI 0.519–5.862), 1.650 (95% CI 0.361–3.875) and 2.536 (95% CI 0.458–6.290)per 1000 person-years respectively in active compared to control arms.

**Table 3:** Effect of cumulative THC:CBD combination treatment across THC:CBD studies expressed as incidence rate difference (IRD, indicated by the summary estimate, followed by 95% confidence intervals and associated *P* value).

MedDRA high-level grouping	Individual AE	Summary estimate	95% CI (lower, upper)	*P* value	N	Q	Qp
Blood/Lymphatic System	Anaemia	0.003	0.115, 0.204	0.780	14	2.248	1.000
Cardiac	Dyspnoea	0.004	0.112, 0.212	0.757	11	1.349	0.999
	Palpitation	0.002	0.153, 0.227	0.847	10	8.871	0.449
Ear & Labyrinth	Vertigo	1.602	0.001, 6.579	0.056	**11**	**24.747**	**0.006**
Eye Disorders	Visual impairment/disturbances	0.354	0.107, 2.305	0.206	9	7.400	0.494
**Gastrointestina**l	**Nausea**	**0.674**	**0.100, 1.754**	**0.001**	21	24.371	0.227
	**Vomiting**	**0.214**	**0.000, 0.837**	**0.045**	19	23.852	0.160
	**Dry mouth**	**1.227**	**0.093, 3.650**	**0.007**	**17**	**52.664**	**<0.001**
**General**	Pain: non-specific	0.005	0.276, 0.444	0.818	14	20.025	0.095
	**Fatigue/tiredness**	**0.439**	**0.025, 1.361**	**0.010**	**19**	**29.920**	**0.038**
	Weakness/reduced mobility	0.008	0.107, 0.258	0.670	14	5.839	0.952
Infections	Unspecified	0.002	0.159, 0.230	0.858	8	0.015	1.000
	UTI	0.005	0.111, 0.219	0.740	11	0.580	1.000
	RTI	0.000	0.142, 0.176	0.916	11	2.693	0.988
Injury/Poisoning	Falls & injuries	0.136	0.004, 0.644	0.096	10	4.121	0.903
Investigations	Raised Gamma GT	0.000	0.177, 0.201	0.951	10	0.323	1.000
Metabolism/Nutritional	Decreased appetite	0.011	0.076, 0.237	0.585	16	8.888	0.883
	Increased appetite	0.067	0.065, 0.600	0.323	10	6.947	0.643
	Anorexia	0.010	0.078, 0.228	0.606	14	13.756	0.391
Musculoskeletal	Back pain	0.000	0.187, 0.194	0.984	10	0.169	1.000
	Spasm stiffness	0.002	0.153, 0.232	0.839	10	5.902	0.750
	Musculoskeletal pain	0.000	0.178, 0.205	0.946	9	0.682	1.000
Neoplasms	Neoplasms progression	0.009	0.074, 0.217	0.606	16	9.477	0.851
**Nervous System**	Altered taste	0.237	0.039, 1.367	0.163	13	16.906	0.153
	**Dizziness/Lightheaded**	**2.467**	**0.519, 5.862**	**<0.001**	**24**	**75.465**	**<0.001**
	Headache/migraine	0.024	0.037, 0.250	0.385	18	19.517	0.300
	Numbness/paraesthesia	0.009	0.284, 0.117	0.668	9	0.111	1.000
**Psychiatric**	Sleep problems	0.001	0.148, 0.190	0.904	12	13.621	0.255
	Dissociative/Thinking/Perception	0.010	0.098, 0.258	0.640	12	12.317	0.340
	**Somnolence/Drowsiness**	**1.650**	**0.361, 3.875**	**<0.001**	**19**	**32.569**	**0.019**
	Anxiety/Depression	0.716	0.085, 3.933	0.145	**11**	**30.209**	**0.001**
	Concentration/attention problem	0.277	0.060, 1.685	0.181	11	15.614	0.111
	**Disorientation**	**2.536**	**0.458, 6.290**	**0.001**	**15**	**40.301**	**<0.001**
Renal and Urinary	Renal & urinary symptoms	0.001	0.161, 0.214	0.890	10	5.094	0.826
Respiratory/Thoracic	Nose Tenderness	0.001	0.136, 0.192	0.867	10	0.165	1.000
Skin/Subcutaneous	Other skin problem	0.029	0.072, 0.367	0.448	9	5.806	0.669
	Rash	0.000	0.150, 0.177	0.935	10	0.038	1.000
	Pressure Sore	0.059	0.037, 0.463	0.274	9	0.605	1.000
Vascular	Hypotension	0.003	0.141, 0.243	0.792	10	5.880	0.752

N = number of studies included in analysis. QE = test statistic for the test of heterogeneity. QEp = p value for the test of heterogeneity. NA = not applicable. Statistically significant results are presented in bold.

Meta-regression analyses suggested a significant association between individual AEs and weekly doses of THC and CBD and their interaction expressed as IRSD (Table 4) for some of the AEs such as palpitations (CBD and THC*CBD interaction), altered taste (CBD), dizziness and lightheadedness (THC), concentration and attention problems (THC, CBD, THC*CBD interaction) and disorientation (THC).

**Table 4:** Effect of cumulative THC and CBD doses per week and THC*CBD dose interaction across THC:CBD combination studies expressed as square root transformed incidence rate difference (IRSD, i.e. summary estimate) for each single adverse event. Summary estimates (i.e. regression coefficient) are reported here for intercept and THC and CBD doses as well as their interaction, followed by 95% confidence intervals and associated *P* value.

MedDRA high-level grouping	Individual AE	Model	Summary estimate	95% CI (lower, upper)	*P* value	N	QE	QEp
Blood/Lymphatic System	Anaemia	Intercept	−0.007	−0.089, 0.075	0.871	14	0.773	1.000
		THC	0.000	−0.001, 0.000	0.890	14		
		CBD	0.000	−0.001, 0.001	0.889	14		
		THC*CBD interaction	0.000	0.000, 0.000	0.815	14		
**Cardiac**	Dyspnoea	Intercept	0.067	−0.073, 0.208	0.346	11	0.047	1.000
		THC	0.000	−0.002, 0.001	0.422	11		
		CBD	−0.001	−0.007, 0.005	0.730	11		
		THC*CBD interaction	0.000	0.000, 0.000	0.724	11		
	**Palpitation**	Intercept	0.437	−0.132, 1.005	0.132	10	1.919	0.927
		THC	−0.004	−0.008, 0.000	0.053	10		
		**CBD**	**−0.008118**	**−0.016085, −0.000151**	**0.046**	10		
		THC*CBD interaction	**0.000066**	**0.000008, 0.000124**	**0.026**	10		
Ear & Labyrinth	Vertigo	Intercept	0.024	−0.295, 0.344	0.881	11	5.204	0.635
		THC	−0.001	−0.002, 0.001	0.535	11		
		CBD	0.001	−0.001, 0.002	0.352	11		
		THC*CBD interaction	0.000	0.000, 0.000	0.920	11		
Eye Disorders	Visual impairment/disturbances	Intercept	0.018	−1.309, 1.345	0.979	9	6.621	0.250
		THC	0.000	−0.012, 0.011	0.941	9		
		CBD	0.000	−0.028, 0.028	0.999	9		
		THC*CBD interaction	0.000	0.000, 0.000	0.966	9		
Gastrointestinal	Nausea	Intercept	0.023	−0.054, 0.100	0.555	21	14.301	0.646
		THC	0.000	−0.001, 0.000	0.112	21		
		CBD	0.000	0.000, 0.001	0.202	21		
		THC*CBD interaction	0.000	0.000, 0.000	0.925	21		
	Vomiting	Intercept	−0.027	−0.159, 0.105	0.687	19	13.342	0.576
		THC	0.000	−0.001, 0.001	0.848	19		
		CBD	0.000	−0.001, 0.001	0.410	19		
		THC*CBD interaction	0.000	0.000, 0.000	0.930	19		
	Dry Mouth	Intercept	0.020	−0.121, 0.162	0.777	17	49.346	<0.001
		THC	0.000	−0.001, 0.001	0.561	17		
		CBD	0.001	0.000, 0.002	0.323	17		
		THC*CBD interaction	0.000	0.000, 0.000	0.643	17		
General	Pain: non-specific	Intercept	0.029	−0.053, 0.111	0.493	14	18.617	0.045
		THC	0.000	−0.001, 0.000	0.698	14		
		CBD	0.000	−0.002, 0.001	0.475	14		
		THC*CBD interaction	0.000	0.000, 0.000	0.316	14		
	Fatigue/tiredness	Intercept	0.024	−0.054, 0.102	0.544	19	24.526	0.057
		THC	0.000	−0.001, 0.000	0.266	19		
		CBD	0.000	0.000, 0.001	0.543	19		
		THC*CBD interaction	0.000	0.000,0.000	0.714	19		
	Weakness/reduced mobility	Intercept	−0.041	−0.202, 0.121	0.623	14	5.009	0.891
		THC	0.000	−0.001, 0.001	0.801	14		
		CBD	0.001	−0.001, 0.002	0.399	14		
		THC*CBD interaction	0.000	0.000, 0.000	0.450	14		
Infections	Unspecified	Intercept	−0.007	−1.334, 1.321	0.992	8	0.014	1.000
		THC	0.000	−0.011, 0.011	0.994	8		
		CBD	0.000	−0.028, 0.028	0.991	8		
		THC*CBD interaction	0.000	0.000, 0.000	0.991	8		
	UTI	Intercept	−0.063	−0.867, 0.742	0.879	11	0.534	0.999
		THC	0.001	−0.006, 0.007	0.876	11		
		CBD	0.001	−0.014, 0.016	0.859	11		
		THC*CBD interaction	0.000	0.000, 0.000	0.865	11		
	RTI	Intercept	0.078	−0.279, 0.435	0.668	11	0.476	1.000
		THC	0.000	−0.002, 0.002	0.674	11		
		CBD	−0.002	−0.005, 0.001	0.242	11		
		THC*CBD interaction	0.000	0.000, 0.000	0.238	11		
Injury/Poisoning	Falls & injuries	Intercept	−0.251	−0.820, 0.317	0.386	10	1.252	0.974
		THC	0.002	−0.002, 0.007	0.286	10		
		CBD	0.005	−0.003, 0.013	0.183	10		
		THC*CBD interaction	0.000	0.000, 0.000	0.172	10		
Investigations	Raised Gamma GT	Intercept	0.077	−0.434, 0.587	0.768	10	0.015	1.000
		THC	−0.001	−0.004, 0.003	0.699	10		
		CBD	−0.002	−0.010, 0.007	0.707	10		
		THC*CBD interaction	0.000	0.000,0.000	0.671	10		
Metabolism/Nutritional	Decreased Appetite	Intercept	0.053	−0.031, 0.137	0.216	16	4.443	0.974
		THC	0.000	−0.001,0.000	0.296	16		
		CBD	0.000	0.000,0.000	0.080	16		
		THC*CBD interaction	0.000	0.000,0.000	0.263	16		
	Increased Appetite	Intercept	−0.071	−0.571, 0.428	0.779	10	2.657	0.850
		THC	0.000	−0.003, 0.003	0.884	10		
		CBD	0.002	−0.006, 0.011	0.616	10		
		THC*CBD interaction	0.000	0.000,0.000	0.697	10		
	Anorexia	Intercept	−0.103	−0.249, 0.043	0.167	14	10.513	0.397
		THC	0.000	0.000, 0.001	0.272	14		
		CBD	0.001	0.000, 0.002	0.158	14		
		THC*CBD interaction	0.000	0.000,0.000	0.229	14		
Musculoskeletal	Back pain	Intercept	0.084	−1.168, 1.335	0.896	10	0.138	1.000
		THC	−0.001	−0.011, 0.010	0.898	10		
		CBD	−0.002	−0.028, 0.024	0.887	10		
		THC*CBD interaction	0.000	0.000, 0.000	0.891	10		
	Spasm stiffness	Intercept	0.300	−0.952, 1.551	0.639	10	4.242	0.644
		THC	−0.003	−0.014,0.008	0.578	10		
		CBD	−0.006	−0.032,0.020	0.659	10		
		THC*CBD interaction	0.000	0.000,0.000	0.617	10		
	Musculoskeletal pain	Intercept	−0.173	−1.500, 1.154	0.799	9	0.026	1.000
		THC	0.001	−0.010, 0.012	0.859	9		
		CBD	0.002	−0.026, 0.029	0.915	9		
		THC*CBD interaction	0.000	0.000,0.000	0.929	9		
Neoplasms	Neoplasms progression	Intercept	0.120	−0.040, 0.279	0.142	16	6.544	0.886
		THC	−0.001	−0.001,0.000	0.123	16		
		CBD	−0.001	−0.002, 0.001	0.340	16		
		THC*CBD interaction	0.000	0.000,0.000	0.283	16		
**Nervous system**	**Altered taste**	Intercept	−0.077	−0.226, 0.072	0.311	13	8.557	0.479
		THC	0.000	−0.001,0.001	0.759	13		
		**CBD**	**0.001**	**0.000,0.002**	**0.024**	13		
		THC*CBD interaction	0.000	0.000,0.000	0.141	13		
	**Dizziness/Lightheaded**	Intercept	−0.018	−0.077,0.041	0.549	24	60.352	<0.001
		**THC**	**0.000498**	**0.000080, 0.000916**	**0.020**	**24**		
		CBD	0.000	0.000,0.000	0.628	24		
		THC*CBD interaction	0.000	0.000,0.000	0.602	24		
	Headache/migraine	Intercept	0.087	−0.055,0.228	0.229	18	14.376	0.422
		THC	−0.001	−0.001,0.000	0.102	18		
		CBD	0.000	−0.001,0.001	0.816	18		
		THC*CBD interaction	0.000	0.000,0.000	0.651	18		
	Numbness/paraesthesia	Intercept	−0.016	−1.343, 1.311	0.981	9	0.106	1.000
		THC	0.000	−0.011, 0.011	0.985	9		
		CBD	0.000	−0.027, 0.028	0.977	9		
		THC*CBD interaction	0.000	0.000,0.000	0.979	9		
**Psychiatric**	Sleep problems	Intercept	0.045	−0.117,0.206	0.587	12	12.044	0.149
		THC	0.000	−0.001,0.001	0.973	12		
		CBD	−0.001	−0.002,0.001	0.311	12		
		THC*CBD interaction	0.000	0.000,0.000	0.459	12		
	Dissociative/Thinking/Perception	Intercept	0.026	−0.135, 0.188	0.751	12	9.068	0.337
		THC	0.000	−0.001, 0.001	0.718	12		
		CBD	−0.001	−0.003,0.000	0.162	12		
		THC*CBD interaction	0.000	0.000,0.000	0.294	12		
	Somnolence/Drowsiness	Intercept	0.066	−0.067, 0.199	0.332	19	11.992	0.680
		THC	−0.001	−0.001,0.000	0.063	19		
		CBD	0.000	−0.001,0.001	0.500	19		
		THC*CBD interaction	0.000	0.000,0.000	0.465	19		
	Anxiety/Depression	Intercept	−0.027	−0.515, 0.461	0.915	11	16.508	0.021
		THC	0.000	−0.003,0.003	0.887	11		
		CBD	0.002	−0.006,0.010	0.580	11		
		THC*CBD interaction	0.000	0.000,0.000	0.733	11		
	**Concentration/attention problem**	Intercept	0.502257	0.145686, 0.858829	0.006	11	1.154	0.992
		**THC**	**−0.003128**	**−0.005162, −0.001094**	**0.003**	11		
		**CBD**	**−0.003718**	**−0.006809, −0.000628**	**0.018**	11		
		THC*CBD interaction	**0.000024**	**0.000007, 0.000041**	**0.006**	11		
	**Disorientation**	Intercept	0.103	−0.045, 0.251	0.173	15	8.917	0.630
		**THC**	**−0.001014**	**−0.001833, −0.000194**	**0.015**	15		
		CBD	0.001	−0.001,0.002	0.315	15		
		THC*CBD interaction	0.000	0.000,0.000	0.386	15		
Renal and Urinary	Renal & urinary symptoms	Intercept	0.490	−0.314, 1.295	0.233	10	1.199	0.977
		THC	−0.004	−0.011,0.003	0.237	10		
		CBD	−0.011	−0.026,0.004	0.154	10		
		THC*CBD interaction	0.000	0.000,0.000	0.178	10		
Respiratory/Thoracic	Nose Tenderness	Intercept	0.024	−0.475, 0.523	0.925	10	0.001	1.000
		THC	0.000	−0.003,0.003	0.895	10		
		CBD	−0.001	−0.009,0.008	0.906	10		
		THC*CBD interaction	0.000	0.000,0.000	0.888	10		
Skin/Subcutaneous	Other skin problem	Intercept	0.049	−1.277, 1.376	0.942	9	2.369	0.796
		THC	0.000	−0.011, 0.012	0.972	9		
		CBD	−0.002	−0.030, 0.026	0.883	9		
		THC*CBD interaction	0.000	0.000,0.000	0.952	9		
	Rash	Intercept	0.012	−0.488, 0.511	0.964	10	0.000	1.000
		THC	0.000	−0.003,0.003	0.949	10		
		CBD	0.000	−0.009,0.008	0.955	10		
		THC*CBD interaction	0.000	0.000,0.000	0.946	10		
	Pressure Sore	Intercept	−0.041	−1.368, 1.286	0.952	9	0.568	0.989
		THC	0.000	−0.011, 0.012	0.961	9		
		CBD	0.001	−0.027, 0.029	0.941	9		
		THC*CBD interaction	0.000	0.000,0.000	0.946	9		
Vascular	Hypotension	Intercept	0.328	−0.182, 0.839	0.207	10	0.276	1.000
		THC	−0.003	−0.006, 0.001	0.099	10		
		CBD	−0.007	−0.016,0.002	0.109	10		
		THC*CBD interaction	0.000	0.000,0.000	0.070	10		

Statistically significant results are presented in bold. A summary estimate of the intercept model represents the square root transformed incidence rate difference (IRSD) of a given AE when the doses of THC and CBD dose per week are 0. The incidence rate at a particular dose combination of THC and CBD formulation may be estimated using the formula: IRSD = intercept + summary estimate * (THC dose per week) + summary estimate * (CBD dose per week) + summary estimate * (THC dose per week)* (CBD dose per week)]. For example, the square root transformed incident rate difference (IRSD) of developing disorientation if a patient is taking a THC:CBD combination formulation containing 10 mg of THC per week and 10 mg of CBD per week will be IRSD = 0.103 + (−0.001014)*10 + (0.001)*10 + (0.000)*10*10 = 0.10286. To convert IRSD per person-year into incidence rate difference (IRD) per 1000 person-years (i.e. incidence rate difference associated with cumulative exposure at the specified doses of THC and CBD in combination over 1 year for 1000 individuals) one would need to use the formula (IRSD^2^ * 1000). Using this formula, the additional incidence (IRD) of disorientation attributable to THC:CBD combination exposure of 10 mg/week of THC and 10 mg/week of CBD in 1000 people over 1 year amounts to 10.58 per 1000 person-years. Therefore, in a sample of 1000 individuals taking a THC: CBD combination treatment containing 10 mg of THC and 10 mg of CBD per week over a 1-year period, 10.58 additional individuals will experience dizziness/lightheadedness attributable to their THC:CBD combination treatment.

Pooled IRDs for all cause (*k* = 26) and treatment-related (*k* = 22) SAEs from all RCTs was 0.056 (95% CI, 0.02–0.39) and 0.058 (95% CI, 0.08–0.59) respectively. Pooled IRDs for all cause (*k* = 22) and treatment related (*k* = 27) withdrawals from all RCTs was 0.036 (95% CI, 0.44–0.08) and 0.489 (95% CI, 0.05–1.37) respectively. IRDs for all cause deaths (*k* = 27) from all RCTs were 0.010 (95% CI, 0.04–0.17).

Neither Egger’s test nor ‘Trim and fill’ method indicated significant effect of publication or other selection bias for any of the outcomes except for all cause AEs (Supplementary Figs 21a-d, 22a-f, 23a-e, 24a-c). For all cause AEs for all RCTs as outcome, Egger’s test was p = 0.0332, and ‘Trim and fill’ method indicated two missing studies. Meta-regression analysis indicated that there was a significant effect of neurodegenerative disorder on effect-size for all cause withdrawals (p = 0.044) compared to other conditions. Except these, moderators such as study design or type of intervention did not significantly influence estimated effects of THC:CBD combination treatment on any of the outcomes assessed.

## Discussion

In this systematic review and meta-analysis, we estimated the additional risk of organ-specific and total AEs attributable to exposure with medicinal cannabinoids in middle aged and older adults by assessing incidence rate differences of AEs. For medications containing THC-alone, on average this amounted to an additional incidence of ~19 all-cause and ~16 treatment-related AEs, whilst for THC:CBD combination treatments, there was an additional incidence of ~19 all-cause and ~11 treatment-related AEs per 1000 person-years of exposure.

Importantly, in this meta-analysis, we identified specific AEs associated with THC in THC alone or THC: CBD combination treatments. We found that THC significantly increased the incidence of dizziness/lightheadedness, somnolence/drowsiness, impaired mobility/balance/coordination, sedation, headache, dissociative/thinking/perception disorders, euphoria and dry mouth amounting on average to an additional incidence from ˂1 to ~11 per 1000 person-years, respectively. Further, there was a dose-dependent increase in the additional incidence of the aforementioned AEs as well as dry mouth and dissociative/thinking/perception problems, such that the higher the weekly dose of THC the higher was the additional attributable incidence of these specific AEs. These individual AEs are worth noting, as they not only impair quality of life but may also contribute to risk of falls in this age group [11, 65, 66], a leading cause of fatal and nonfatal injuries amongst older people [66, 67]. Incidence of psychotic-like experiences such as dissociative/thinking/perception abnormalities was significantly increased in THC alone studies and associated with higher THC doses as noted in our previous observation [9] and can be distressing to patients and their carers.

In addition, further analysis showed that THC and CBD in combination significantly increased the incidence of nausea, vomiting, dry mouth, fatigue/tiredness, dizziness/lightheadedness, somnolence/drowsiness and disorientation, amounting on average to an additional incidence of ˂1 to ~3 per 1000 person-years, respectively. This highlights the need to be mindful of higher weekly doses of THC and CBD in the older population, who are also on other medications for multiple co-morbidities. Furthermore, there was a dose-dependent relationship of weekly CBD doses with palpitation, altered taste and problems of inattention and concentration. Some of these effects are consistent with another meta-analysis, though they also reported abnormal liver function tests, decreased appetite, diarrhoea, pneumonia [10]. However, most of the studies were in those with childhood epilepsies and authors conjectured that this may have been due to interaction of CBD with other medications such as clobazam and/or sodium valproate and excluding these studies showed only diarrhoea as an adverse event for CBD [10]. It is interesting to note that these side-effects were not found in our analysis for middle aged and older adults, although interaction with other medications was not examined.

This report, which includes data from 58 RCTs is an update of our previous meta-analysis summarising 46 RCTs, and confirms that whilst middle aged and older adults are at greater risk of both treatment-related and all cause AEs from CBMs containing THC, they are not associated with SAEs, withdrawals or death [8]. Critically, we extend previous literature by providing the first pooled estimate of incidence of AEs attributable to CBMs. As described before, previous reviews of AEs with CBMs have either been qualitative, did not specifically focus on middle aged and older adults, or did not consider the effects of THC, CBD, or their combination separately [8] or focused on specific clinical indications [7, 68–71]. They have sometimes reported conflicting findings in terms of AEs, likely contributed partly by varying pooled sample sizes, as well as the quality of included studies (details in Supplementary Discussion). In general, those with larger number of pooled participants tend to show a modest but significant increase in risk of AEs as we have reported here, though results vary in terms of specific individual AEs reported [68–71]. By pooling data across all indications, here, we extend previous evidence to provide a more comprehensive and robust CBM-specific summary of the plethora of AEs associated with CBM use in adults over the age of 50. We also provide dose–response relationship estimates that have not been reported before to the best of our knowledge, which may help clinicians and researchers in dosage decisions in different contexts [72]. Further, across different meta-analyses, AEs have commonly been reported in terms of risk ratios, odds ratios or incidence rate ratios. Whilst these metrics are useful to convey whether there are significant additional risks associated with CBMs, they do not lend themselves as easily to everyday use. One needs to be aware of the baseline risk in the control (or placebo) group, which often remains unclear, to estimate the additional incidence associated with exposure to the CBM over a period of time. By estimating the additional incidence rate of all AEs as well as specific AEs associated with CBM use, we hope that results reported here will allow easier use of this information in the clinical and research contexts, especially in terms of estimating and communicating additional risk.

### Limitations

Our review has some limitations. Using GRADE Framework, we found evidence of moderate to high quality evidence in ~64% of studies and low to very low quality in 36% of studies. Some of the trials had inadequate information about randomisation, allocation concealment, selective outcome reporting and objective outcomes which restricts interpretation of results (see supplementary material for full details of bias). However, we included double-blind studies to increase the methodological rigour of the contributing evidence. Therefore, these results need to be considered in light of potential selective reporting of side-effects, often relatively short duration of treatment in included RCTs and imprecision in the estimated IRD values. Notwithstanding this, we provide estimates from a larger pool of patients with indication that publication or other selection biases are unlikely to have influenced the pooled estimates reported here [8]. Further, our dose–response relationship tables may also aid dosage and formulation decisions in clinicians and researchers using CBMs by allowing them to compute ball-park estimates of incidence of AEs at different dose ranges (see footnotes for Tables 2 and 4 for guidance on calculations).

Unlike in other recent meta-analyses, which reported summary effects separately based on indications [7, 68–71], we pooled safety and tolerability data in middle aged and older adults across a broad range of indications. Whilst this may have added to the heterogeneity of the data synthesised, it allowed us to comprehensively estimate separately the tolerability of the two broad categories of cannabinoid-based interventions i.e. THC only and THC:CBD combination, something that has not been done before. This is a key strength of the present approach, given the reported opposite effects of different cannabinoids [3, 73]. Another important strength of the present report relates to the analysis of the effects of moderators to examine the extent to which they may have influenced results, in particular relationship with cannabinoid weekly doses used and interaction.

There is growing evidence that THC and CBD may have opposing acute effects on autonomic arousal, brain [73] and cardiovascular function [74] and CBD may mitigate some of the harmful effects of THC on cognition and behaviour [73, 75, 76], consistent with their opposing effects on some of their molecular targets [3]. The suggestion that THC and CBD may have distinct tolerability profiles, with certain side-effects noticeable in those taking THC-only formulations whilst adverse effects may even be mitigated in those taking THC and CBD in combination, underscored the importance of examining their safety and tolerability separately as well as dose–response relationships as we have done here. Our findings of AEs are consistent with other meta-analyses but in addition show the association of weekly doses with some of the adverse effects of THC and CBD. Few studies have examined the drug–drug interaction of CBMs given their effect on cytochrome p450 enzymes [77], an important likely determinant of tolerability and dose adjustment in older people, and therefore worthy of investigation in future studies.

## Conclusions

Results of the present study suggest that THC-containing CBMs are associated with certain gastrointestinal, neurological and psychiatric side-effects in a dose-related manner, both for THC only and for THC: CBD combinations some of which can be mitigated by CBD. Efficacy should additionally consider dose–response relationships with regard to tolerability whilst prescribing CBMs, particularly in older people.

## Supplementary Material

aa-24-0836-File002_afae261
