# Supplementary material for: Adverse events caused by cannabinoids in middle aged and older adults for all indications: a meta-analysis of incidence rate difference
Source: Age Ageing. 2024 Nov 27;53(11):afae261. doi: 10.1093/ageing/afae261 (PMC11601816; doi:10.1093/ageing/afae261)
Supplement: aa-24-0836-File002_afae261 [file aa-24-0836-file002_afae261.docx]

**Supplementary material**

**Adverse events caused by cannabinoids in middle aged and older adults for all indications: A meta-analysis of incidence rate difference**

Contents

[METHODS 4](#_Toc180600585)

[Search strategy 4](#_Toc180600586)

[Quality assessment 4](#_Toc180600587)

[DISCUSSION: 5](#_Toc180600588)

[FIGURES 7](#_Toc180600589)

[Forest plots for THC and THC:CBD studies 7](#_Toc180600590)

[Figure 1. Forest plot of all cause Adverse Events: THC studies 7](#_Toc180600591)

[Figure 2. Forest plot of treatment-related adverse events: THC studies 7](#_Toc180600592)

[Figure 3. Forest plot of all cause Serious Adverse Events: THC studies 8](#_Toc180600593)

[Figure 4. Forest plot of Treatment-related Serious Adverse Events: THC studies 8](#_Toc180600594)

[Figure 5. Forest plot of all cause Withdrawals: THC studies 9](#_Toc180600595)

[Figure 6. Forest plot of treatment-related Withdrawals: THC studies 9](#_Toc180600596)

[Forest 7. Forest plot of deaths: THC studies 10](#_Toc180600597)

[Figure 8. Forest plot of All cause Adverse Events: THC:CBD studies 10](#_Toc180600598)

[Figure 9. Forest plot of Treatment-related Adverse Events: THC:CBD studies 11](#_Toc180600599)

[Figure 10. Forest plot of all cause serious adverse events: THC:CBD studies 11](#_Toc180600600)

[Figure 11. Forest plot of Treatment-related Serious Adverse Events: THC:CBD studies 12](#_Toc180600601)

[Figure 12. Forest plot of all cause withdrawals: THC:CBD studies 12](#_Toc180600602)

[Figure 13. Forest plot of treatment-related withdrawals: THC:CBD studies 13](#_Toc180600603)

[Figure 14. Forest plot of Deaths: THC:CBD studies 13](#_Toc180600604)

[Quality rating – risk of bias plots 14](#_Toc180600605)

[Figure 15a. Traffic lights plot showing quality ratings for THC studies 14](#_Toc180600606)

[Figure 15b. Overall summary plot showing quality ratings per domain across studies for THC studies. 15](#_Toc180600607)

[Figure 16a. Traffic lights plot showing quality ratings for THC:CBD studies 15](#_Toc180600608)

[Figure 16b. Overall summary plot showing quality ratings per domain across studies for THC:CBD studies 16](#_Toc180600609)

[Publication bias – funnel plots 16](#_Toc180600610)

[Figure 17a. Funnel plots for all-cause adverse events (AEs) meta-analyses for THC studies 16](#_Toc180600611)

[Figure 17b. Funnel plots for all-cause adverse events (AEs) meta-analyses for THC crossover studies 17](#_Toc180600612)

[Figure 17c. Funnel plots for all-cause adverse events (AEs) meta-analyses for THC RCT studies 17](#_Toc180600613)

[Figure 17d. Funnel plots for treatment-related adverse events (AEs) meta-analyses for THC studies 18](#_Toc180600614)

[Figure 17e. Funnel plots for treatment-related adverse events (AEs) meta-analyses for THC crossover studies 18](#_Toc180600615)

[Figure 17f. Funnel plots for treatment-related adverse events (AEs) meta-analyses for THC RCT studies 19](#_Toc180600616)

[Figure 18a. Funnel plots for all-cause serious adverse events (SAEs) meta-analyses for THC studies. 19](#_Toc180600617)

[Figure 18b. Funnel plots for all-cause serious adverse events (SAEs) meta-analyses for THC crossover studies 20](#_Toc180600618)

[Figure 18c. Funnel plots for all-cause serious adverse events (SAEs) meta-analyses for THC RCT studies 20](#_Toc180600619)

[Figure 18d. Funnel plots for treatment-related serious adverse events (SAEs) meta-analyses for THC studies 21](#_Toc180600620)

[Figure 18e. Funnel plots for treatment-related serious adverse events (SAEs) meta-analyses for THC crossover studies 21](#_Toc180600621)

[Figure 18f. Funnel plots for treatment-related serious adverse events (SAEs) meta-analyses for THC RCT studies 22](#_Toc180600622)

[Figure 19a. Funnel plots for all-cause withdrawals meta-analyses for THC studies 22](#_Toc180600623)

[Figure 19b. Funnel plots for all-cause withdrawals meta-analyses for THC RCT studies 23](#_Toc180600624)

[Figure 19c. Funnel plots for treatment-related withdrawals meta-analyses for THC studies. 23](#_Toc180600625)

[Figure 19d. Funnel plots for treatment-related withdrawals meta-analyses for THC crossover studies 24](#_Toc180600626)

[Figure 19e. Funnel plots for treatment-related withdrawals meta-analyses for THC RCT studies 24](#_Toc180600627)

[Figure 20a. Funnel plots for deaths meta-analyses for THC studies 25](#_Toc180600628)

[Figure 20b. Funnel plots for deaths meta-analyses for THC crossover studies 25](#_Toc180600629)

[Figure 20c. Funnel plots for deaths meta-analyses for THC RCT studies 26](#_Toc180600630)

[Figure 21a. Funnel plots for all-cause adverse events (AEs) meta-analyses for THC:CBD studies 26](#_Toc180600631)

[Figure 21b. Funnel plots for all-cause adverse events (AEs) meta-analyses for THC:CBD crossover studies 27](#_Toc180600632)

[Figure 21c. Funnel plots for all-cause adverse events (AEs) meta-analyses for THC:CBD RCT studies 27](#_Toc180600633)

[Figure 21d. Funnel plots for treatment-related adverse events (AEs) meta-analyses for THC:CBD RCT studies 28](#_Toc180600634)

[Figure 22a. Funnel plots for all-cause serious adverse events (SAEs) meta-analyses for THC:CBD studies 28](#_Toc180600635)

[Figure 22b. Funnel plots for all-cause serious adverse events (SAEs) meta-analyses for THC:CBD crossover studies 29](#_Toc180600636)

[Figure 22c. Funnel plots for all-cause serious adverse events (SAEs) meta-analyses for THC:CBD RCT studies 29](#_Toc180600637)

[Figure 22d. Funnel plots for treatment-related serious adverse events (SAEs) meta-analyses for THC:CBD studies 30](#_Toc180600638)

[Figure 22e. Funnel plots for treatment-related serious adverse events (SAEs) meta-analyses for THC:CBD crossover studies 30](#_Toc180600639)

[Figure 22f. Funnel plots for treatment-related serious adverse events (SAEs) meta-analyses for THC:CBD RCT studies 31](#_Toc180600640)

[Figure 23a. Funnel plots for all-cause withdrawals meta-analyses for THC:CBD studies 31](#_Toc180600641)

[Figure 23b. Funnel plots for all-cause withdrawals meta-analyses for THC:CBD RCT studies 32](#_Toc180600642)

[Figure 23c. Funnel plots for treatment-related withdrawals meta-analyses for THC:CBD studies 32](#_Toc180600643)

[Figure 23d. Funnel plots for treatment-related withdrawals meta-analyses for THC:CBD crossover studies 33](#_Toc180600644)

[Figure 23e. Funnel plots for treatment-related withdrawals meta-analyses for THC:CBD RCT studies 33](#_Toc180600645)

[Figure 24a. Funnel plots for deaths meta-analyses for THC:CBD studies 34](#_Toc180600646)

[Figure 24b. Funnel plots for deaths meta-analyses for THC:CBD crossover studies 34](#_Toc180600647)

[Figure 24c. Funnel plots for deaths meta-analyses for THC:CBD RCT studies 34](#_Toc180600648)

[TABLES 36](#_Toc180600649)

[Table 1a: Characteristics of included randomised controlled trials of THC in middle aged and older adults (N=31) 36](#_Toc180600650)

[Table 1b: Characteristics of included randomised controlled trials of CBD:THC in middle aged and older adults (N=27). 39](#_Toc180600651)

[Table 2a. Incidence rate difference for each system of classification (THC studies) 42](#_Toc180600652)

[Table 2b. Incidence rate difference for each system of classification (THC:CBD studies) 42](#_Toc180600653)

[REFERENCES 43](#_Toc180600654)

# METHODS

## Search strategy

Two categories of search terms were used. For subject groups we used: ‘Aged’ OR ‘frail’ OR ‘elderly’ OR ‘older’ OR ‘aging’ OR ‘ageing’ OR ‘geriatric’ OR ‘dementia’ OR ‘Parkinson’s’ OR Alzheimer’s’ OR ‘Huntington’s’ OR ‘demented’. For the intervention, we used: ‘Cannabinoids’ OR ‘cannabinoid’ OR ‘cannabinol’ OR ‘cannabidiol’ OR ‘tetrahydrocannabinol’ OR ‘THC’ OR ‘CBD’ OR ‘Sativex’ OR ‘nabilone’ OR ‘dronabinol’ OR ‘delta-9-tetrahydrocannabinol’ OR ‘delta-THC’ OR ‘medical cannabis’ OR ‘epidiolex’. The existing clinical query ‘Therapy/Broad’ was used in PubMed to select therapeutic studies. We identified additional studies from the reference lists of included studies and review articles. The search was complemented with information from ClinicalTrials.gov. We also contacted authors of the identified studies to clarify further appropriateness of inclusion if needed.

As described previously (1), the initial search strategy (KM and LV) identified 4132 citations (Pubmed n = 1305; OVID (Medline, EMBASE and Psychinfo) n = 2041; CINAHL n =786) on the initial inclusion/exclusion criteria between January 1990 to Oct 2020 leaving final 46 studies for final analysis after removal of duplicates, screening of titles and abstracts, and assessment of full text for inclusion. Further update of literature search until 11^th^ Nov 2021 (SP and MD) provided 26 more citations. Following removal of duplicates (n=0), excluded based on screening of title and abstract (n= 15), and assessed based on the eligibility criteria of the full text (n= 10), we identified one more study (2), so we had a total of 47 studies that could be included with a focus for analysis of randomised trials only. A further search on 12^th^  June 2023 yielded no further studies for inclusion in this review.

## Quality assessment

For assessing the overall quality of evidence, we used the GRADE (Grading of Recommendations Assessment, Development and Evaluation) criteria to rate risk of bias, publication bias, imprecision, inconsistency, indirectness, and magnitude of effect (3). We have summarised the GRADE ratings of very low–, low-, moderate-, or high-quality evidence to reflect the extent to which we have confidence in the effect estimates are correct (4). This was done by one reviewer (KM) and checked by a second reviewer (LV), and disagreements were resolved via discussion with a third reviewer (SB) for articles for articles reviewed until Oct 2020. Following further updated literature search, grading was done by SP, MD and reviewed by LV.

In this approach, as previously described (1), evidence from randomised controlled trials (RCT) is initially rated as “high quality” but can be downgraded up to three levels to “moderate quality”, “low quality”, or “very low quality” based on five categories of limitations. A high-quality rating indicates that we are confident that the true effect is similar to the estimated effect; a very-low-quality rating indicates that the true effect is likely to be substantially different from the estimated effect. Limitations considered are the risk of bias (i.e., whether limitations in study design and execution would bias the effect estimate), indirectness of evidence (e.g., whether the adverse events (AEs) of cannabinoids had to be inferred from indirect evidence), imprecision (i.e., sample sizes, commonality of AE outcomes), and publication bias (i.e., selective publication of studies leading to a systematic bias in the effect estimate).

# DISCUSSION:

As described in our earlier review, previous reviews of AEs with CBMs have either been qualitative, did not specifically focus on middle aged and older adults, or did not consider the effects of THC, CBD, or their combination separately (1). A Cochrane review of CBMs for dementia that included studies considering natural (Namisol) and synthetic THC analogues (dronabinol and nabilone) (5), found no clear differences between groups in numbers of AEs, except for sedation (including lethargy). Evidence for AE outcomes was low or very low with concerns regarding imprecision and indirectness as per the quality assessment of the studies included. Another meta-analysis of 17 RCTs using CBMs for Multiple Sclerosis which included 3161 patients, found a higher risk of AEs with active treatments versus placebo and reported a higher risk of dizziness or vertigo, dry mouth, fatigue, feeling drunk, impaired balance or ataxia, memory impairment, and somnolence with cannabinoids (6). A meta-analysis of published and unpublished 83 studies (40 RCTS, n=3067) with median age under 50 years found pharmaceutical THC-CBD led to significantly more adverse events (pooled AEs) (OR=1.99, 95%CI 1.20:3.29; k=10, n=1495; I 2=59%) in those with mental disorders (7). A recent meta-analysis of 32 RCTs (5174 adult patients) for medical cannabis and cannabinoids for chronic pain at ≥ 1 month follow up (median of the mean age 53 years; interquartile range 50-60 years), with moderate certainty of evidence showed oral medical cannabis small increase in risk of transient cognitive impairment, vomiting, drowsiness, impaired attention, and nausea. High certainty showed higher risk of dizziness at ≥3 months versus <3 months follow-up (8). Another recent meta-analysis of medical cannabis for impaired sleep (39 trials, n=5100) with median of average age 53 years (interquartile range 48-58 years), reported that moderate to high certainty evidence indicated that medical cannabis resulted in a substantial increase in the risk of dizziness for trials with ≥3 months follow-up), and a small increase in the risk of somnolence, dry mouth, fatigue, and nausea compared to placebo (9) .

# FIGURES

## Forest plots for THC and THC:CBD studies

### Figure 1. Forest plot of all cause Adverse Events: THC studies


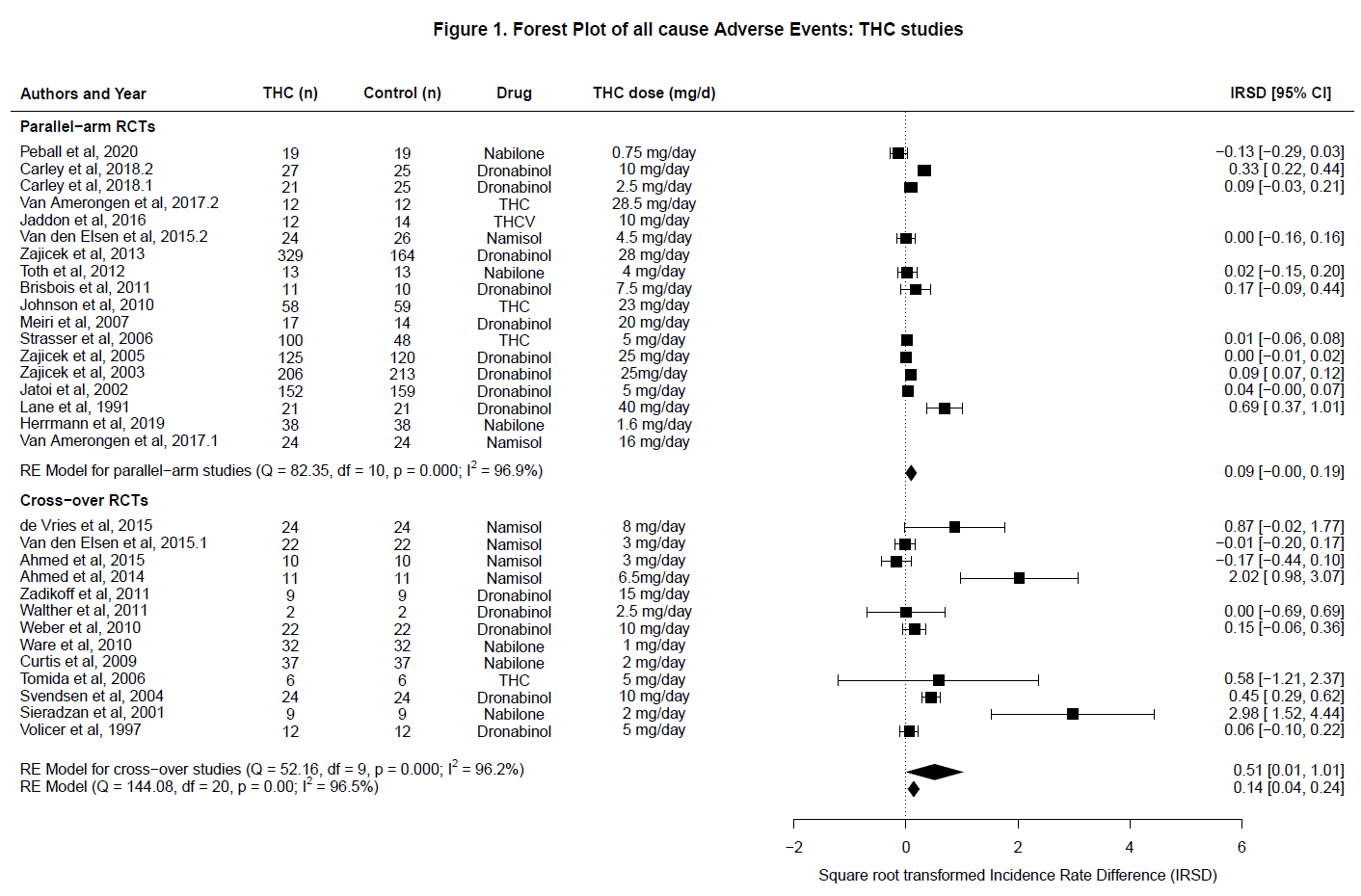


### Figure 2. Forest plot of treatment-related adverse events: THC studies


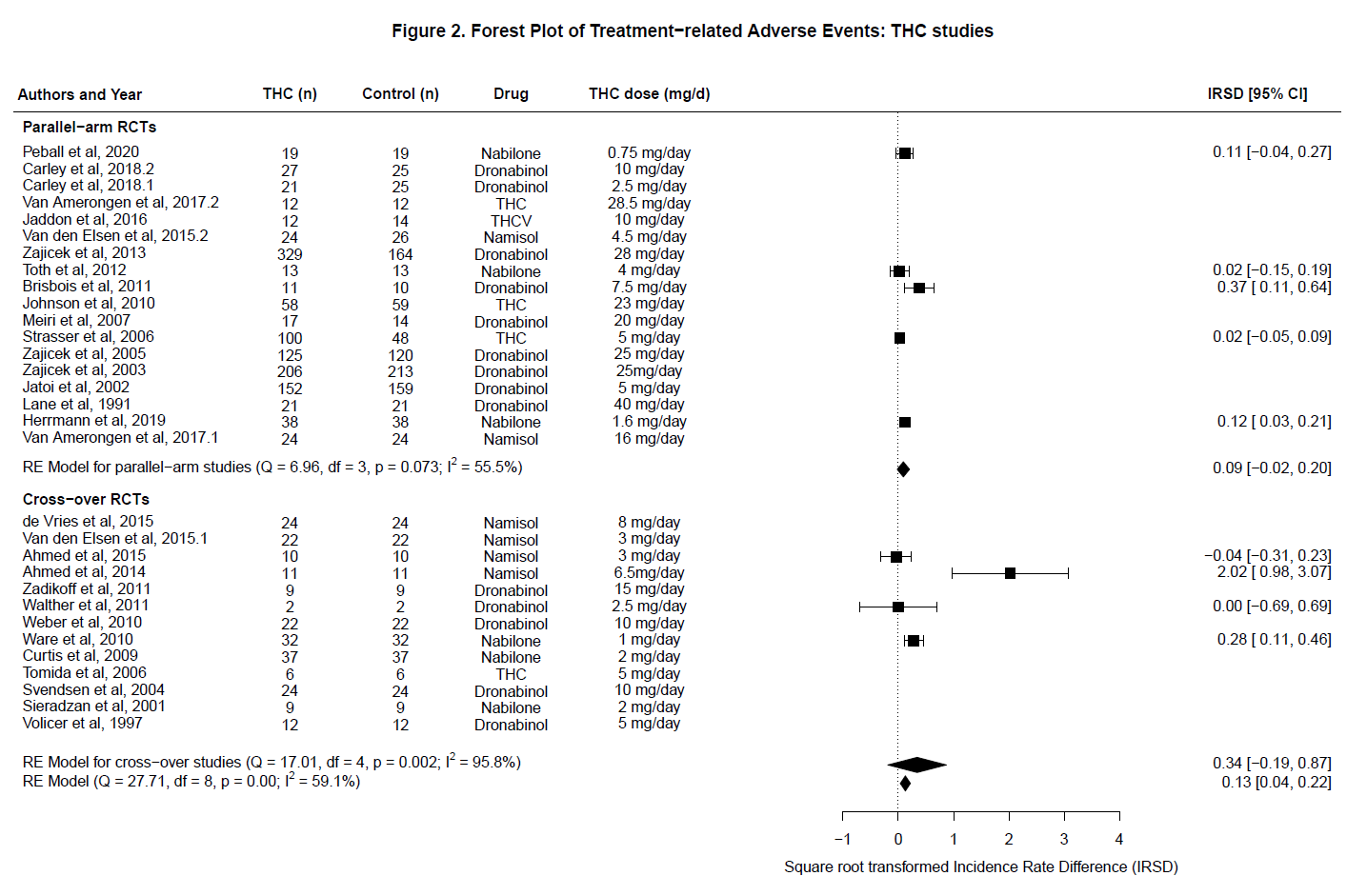


### Figure 3. Forest plot of all cause Serious Adverse Events: THC studies


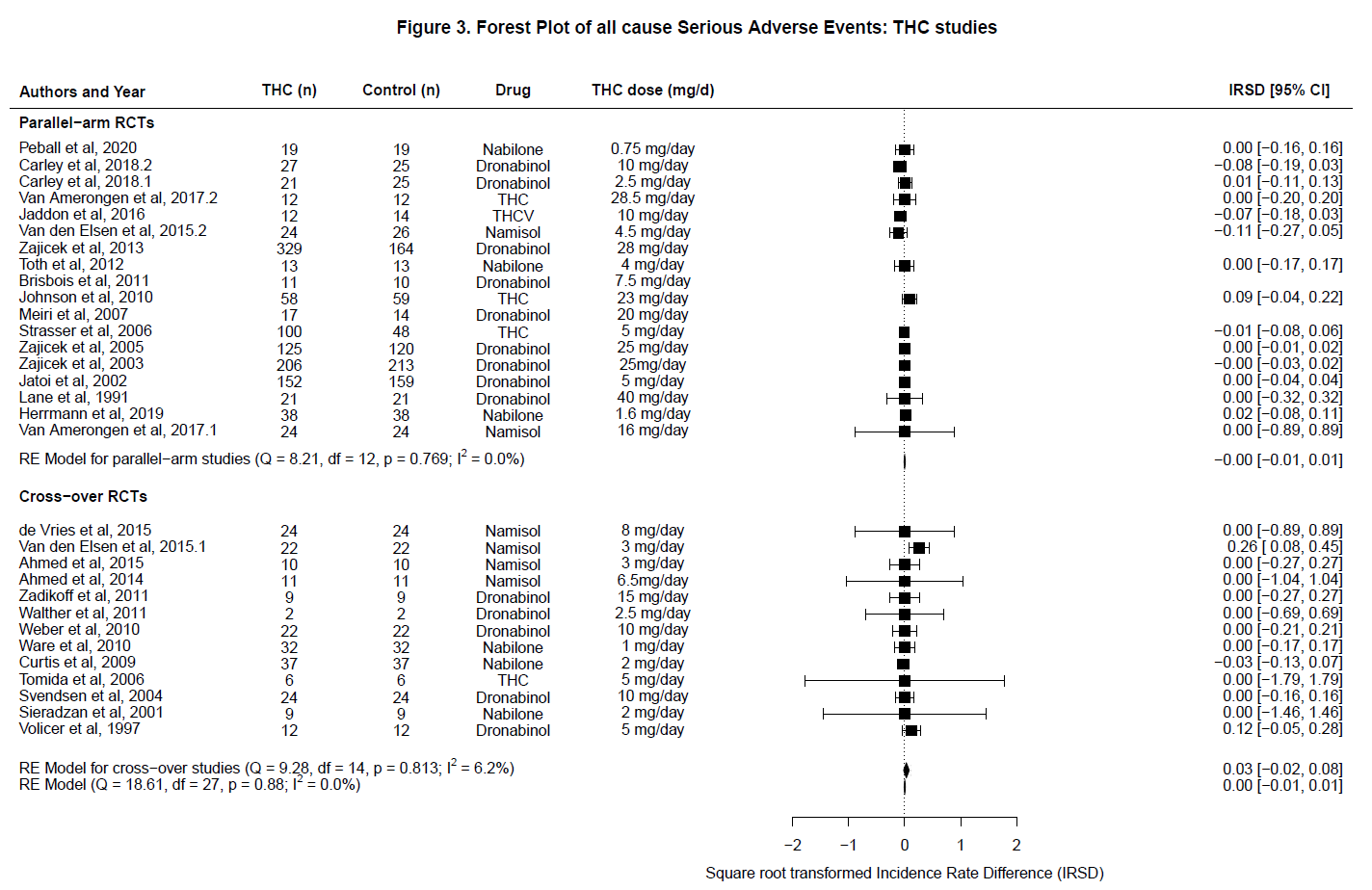


### Figure 4. Forest plot of Treatment-related Serious Adverse Events: THC studies


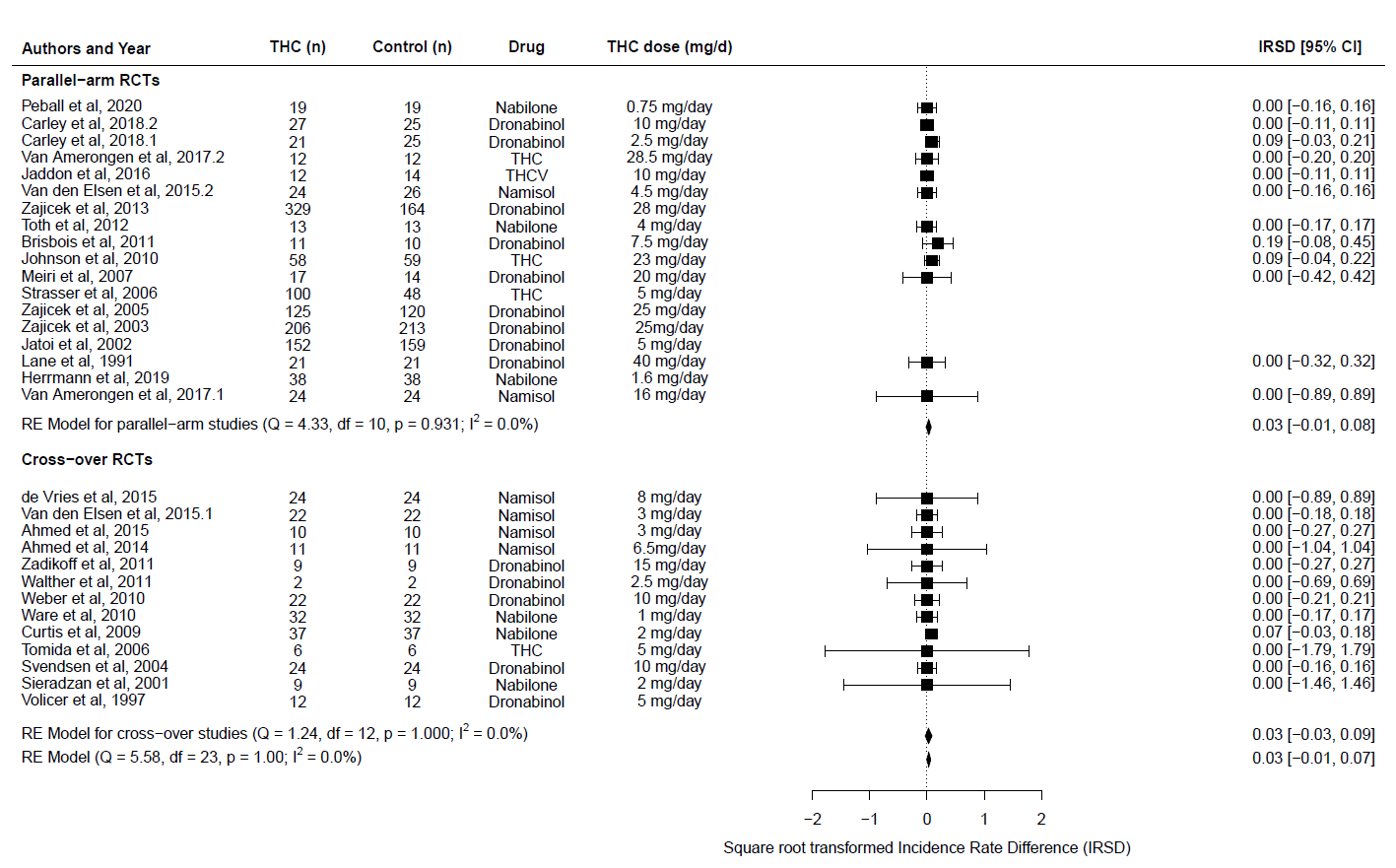


### Figure 5. Forest plot of all cause Withdrawals: THC studies


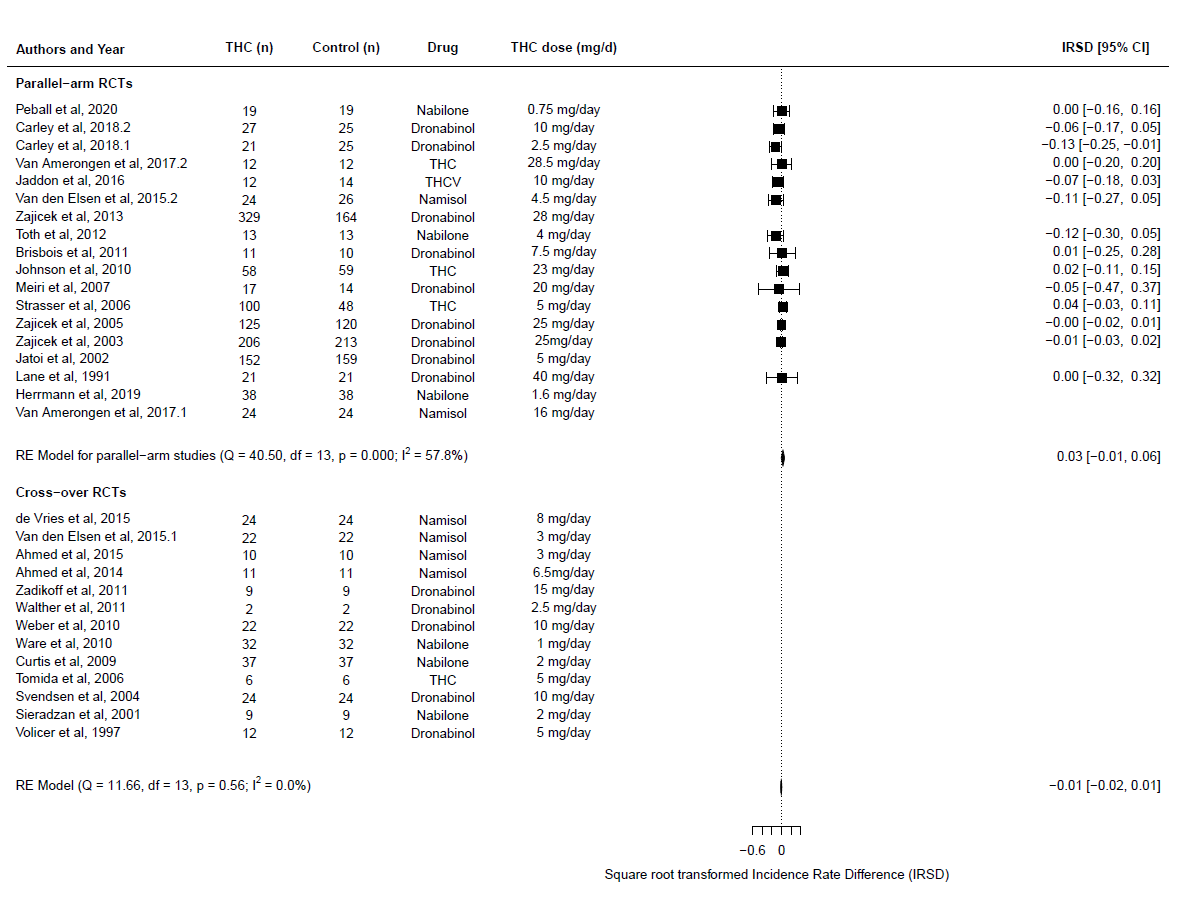


### Figure 6. Forest plot of treatment-related Withdrawals: THC studies


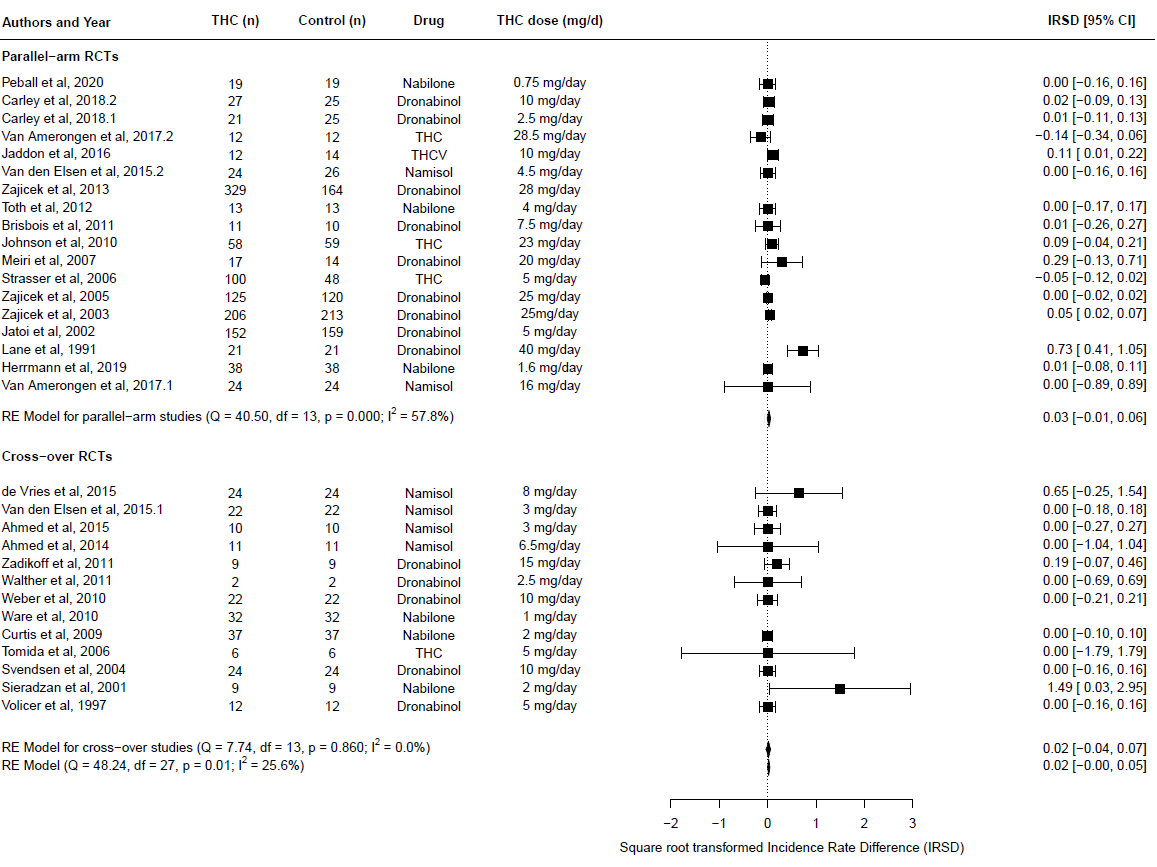


### Forest 7. Forest plot of deaths: THC studies


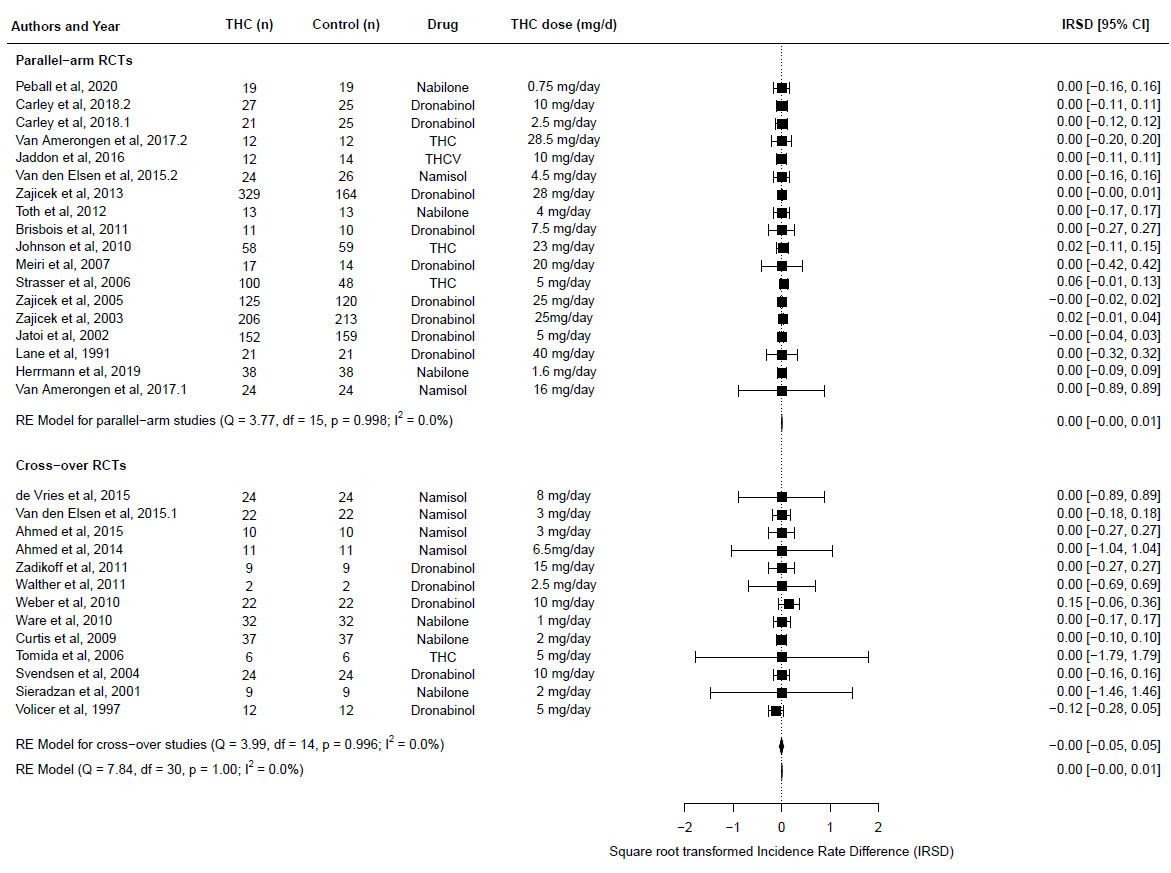


### Figure 8. Forest plot of All cause Adverse Events: THC:CBD studies


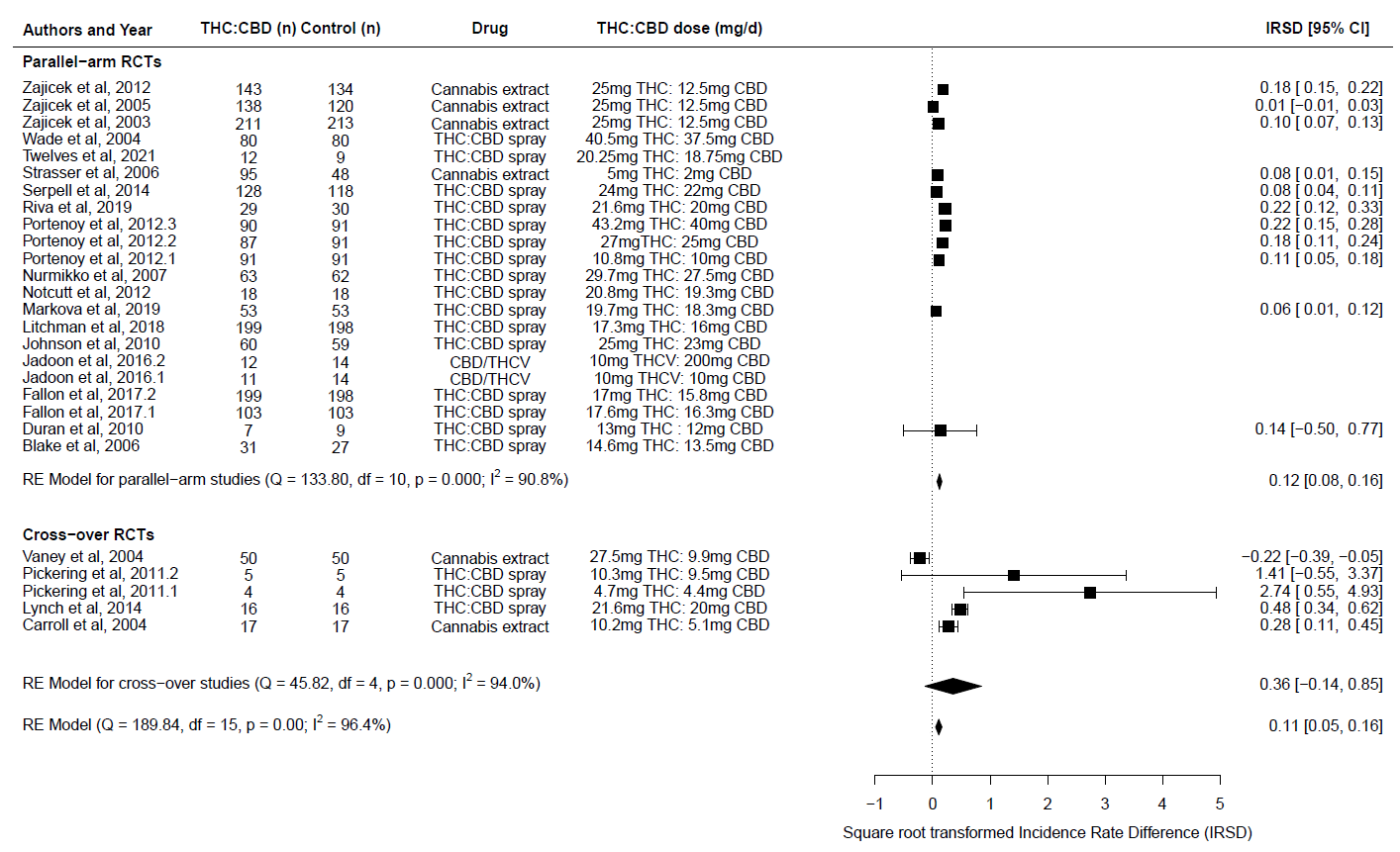


### Figure 9. Forest plot of Treatment-related Adverse Events: THC:CBD studies


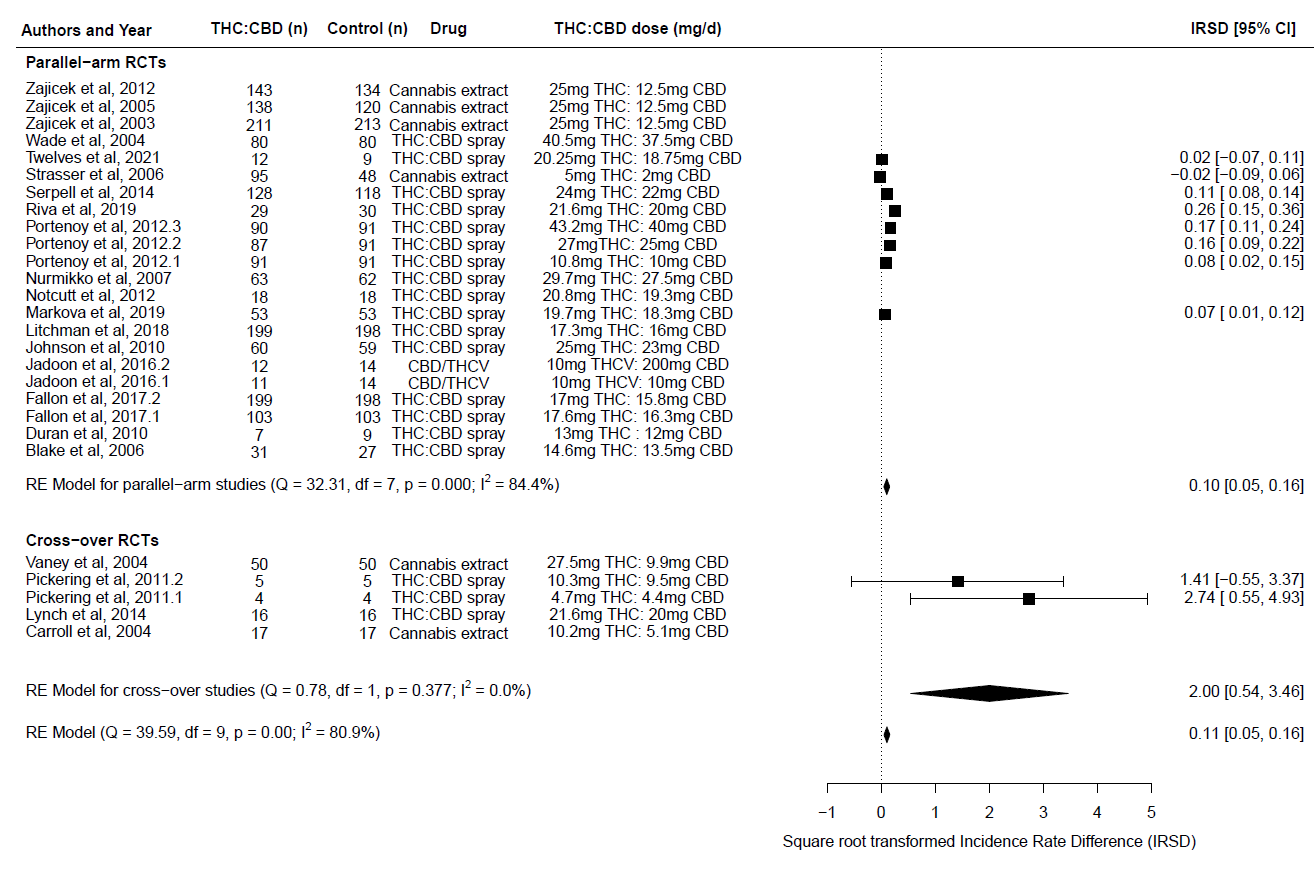


### Figure 10. Forest plot of all cause serious adverse events: THC:CBD studies


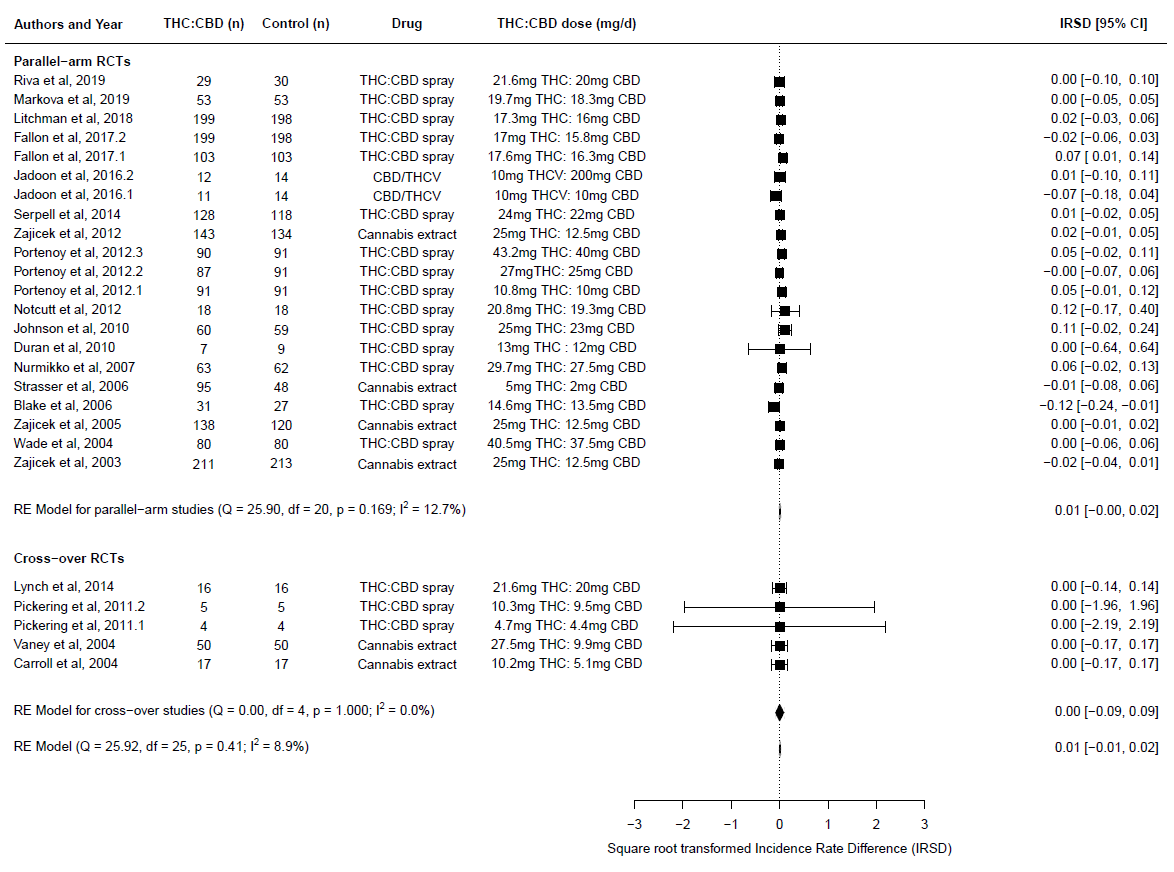


### Figure 11. Forest plot of Treatment-related Serious Adverse Events: THC:CBD studies


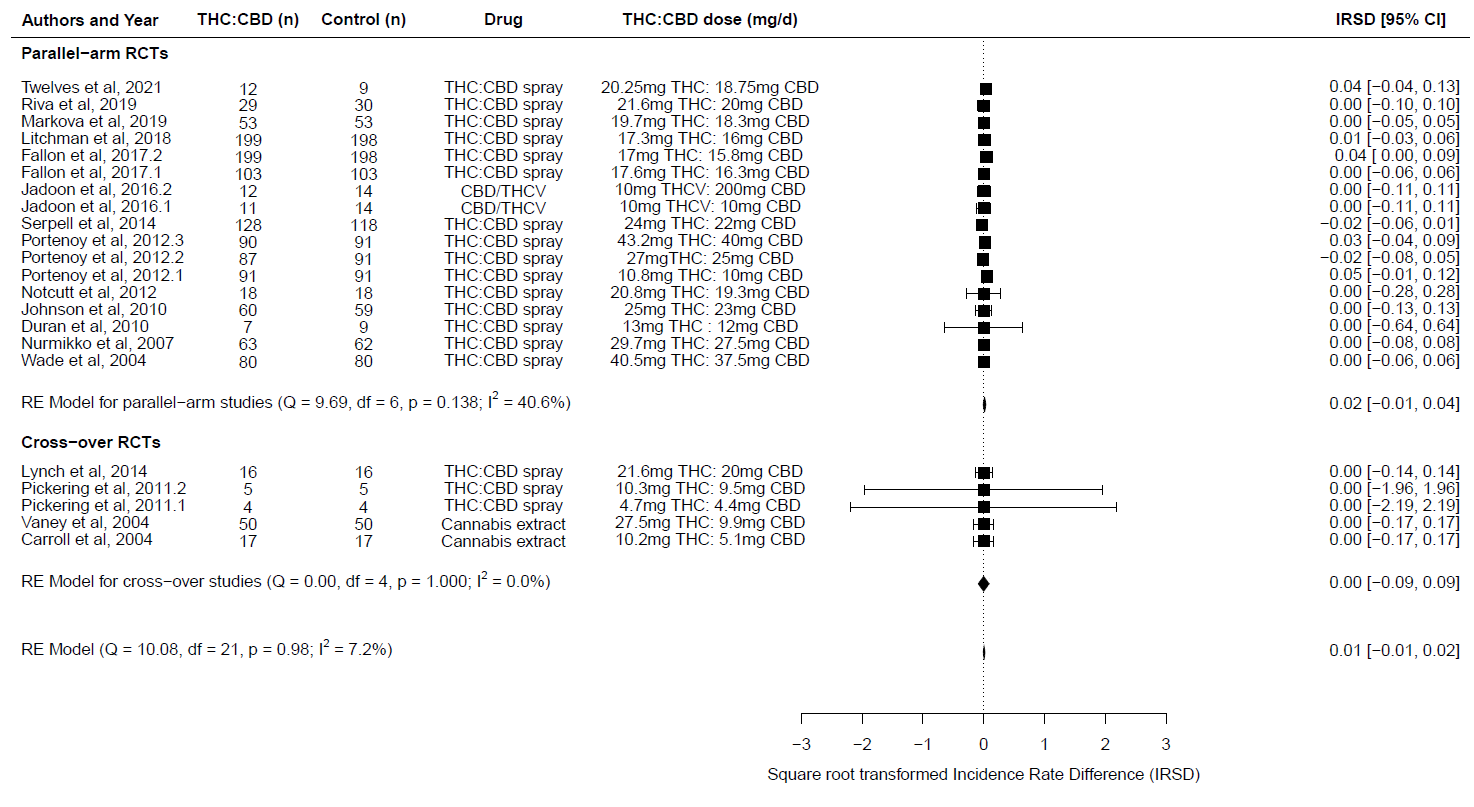


### Figure 12. Forest plot of all cause withdrawals: THC:CBD studies


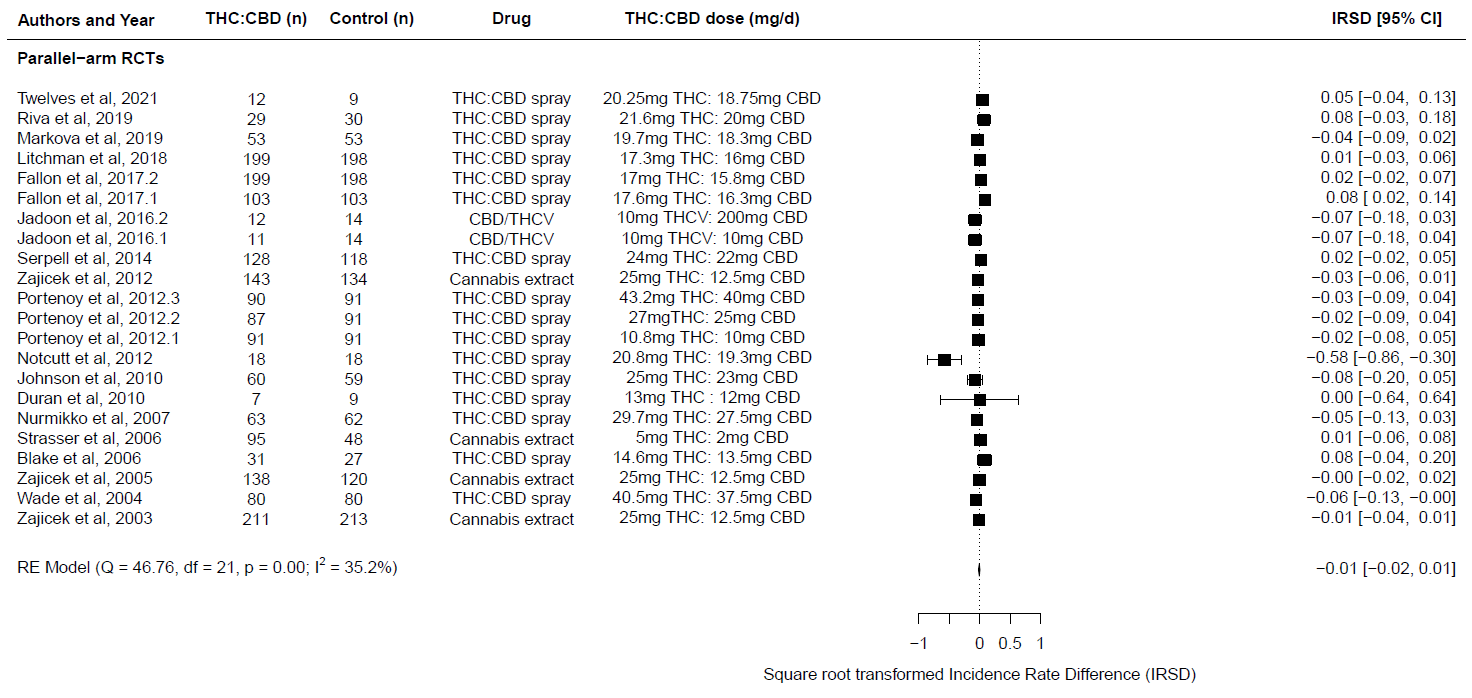


### Figure 13. Forest plot of treatment-related withdrawals: THC:CBD studies


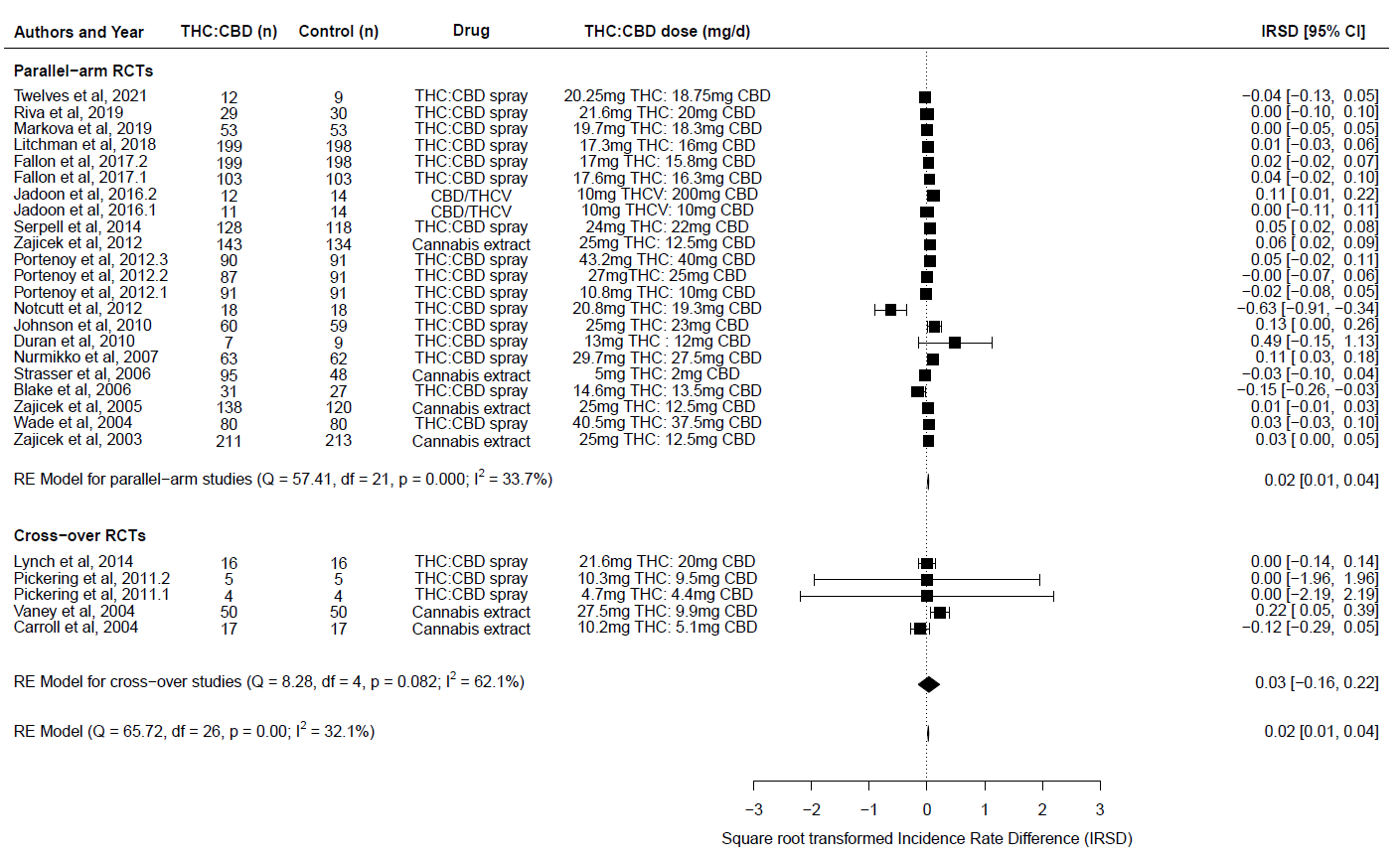


### Figure 14. Forest plot of Deaths: THC:CBD studies


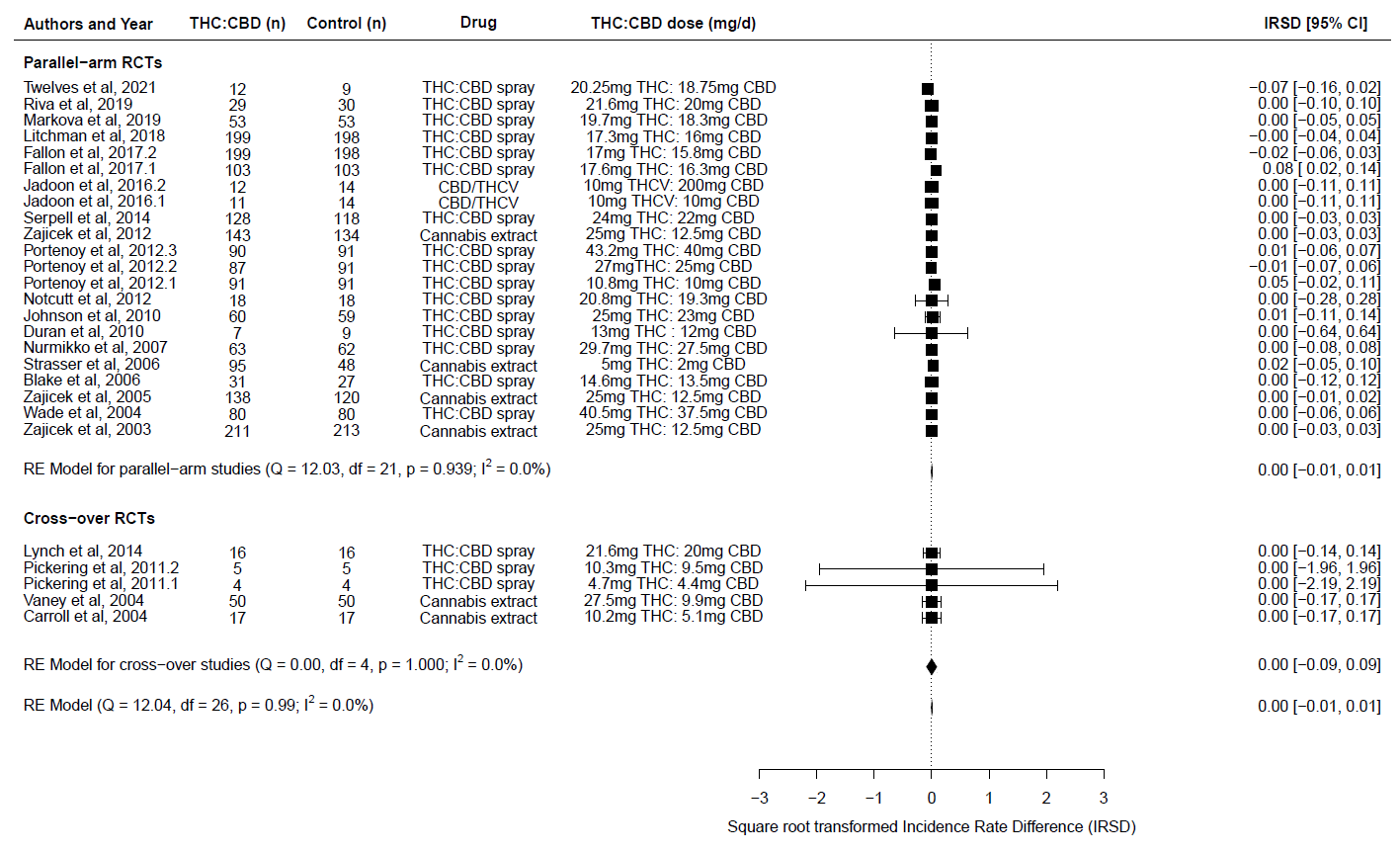


## Quality rating – risk of bias plots

Quality rating plots reporting the rating for each domain based on the GRADE assessment tool. The overall column reports the global rating for each study.

### Figure 15a. Traffic lights plot showing quality ratings for THC studies


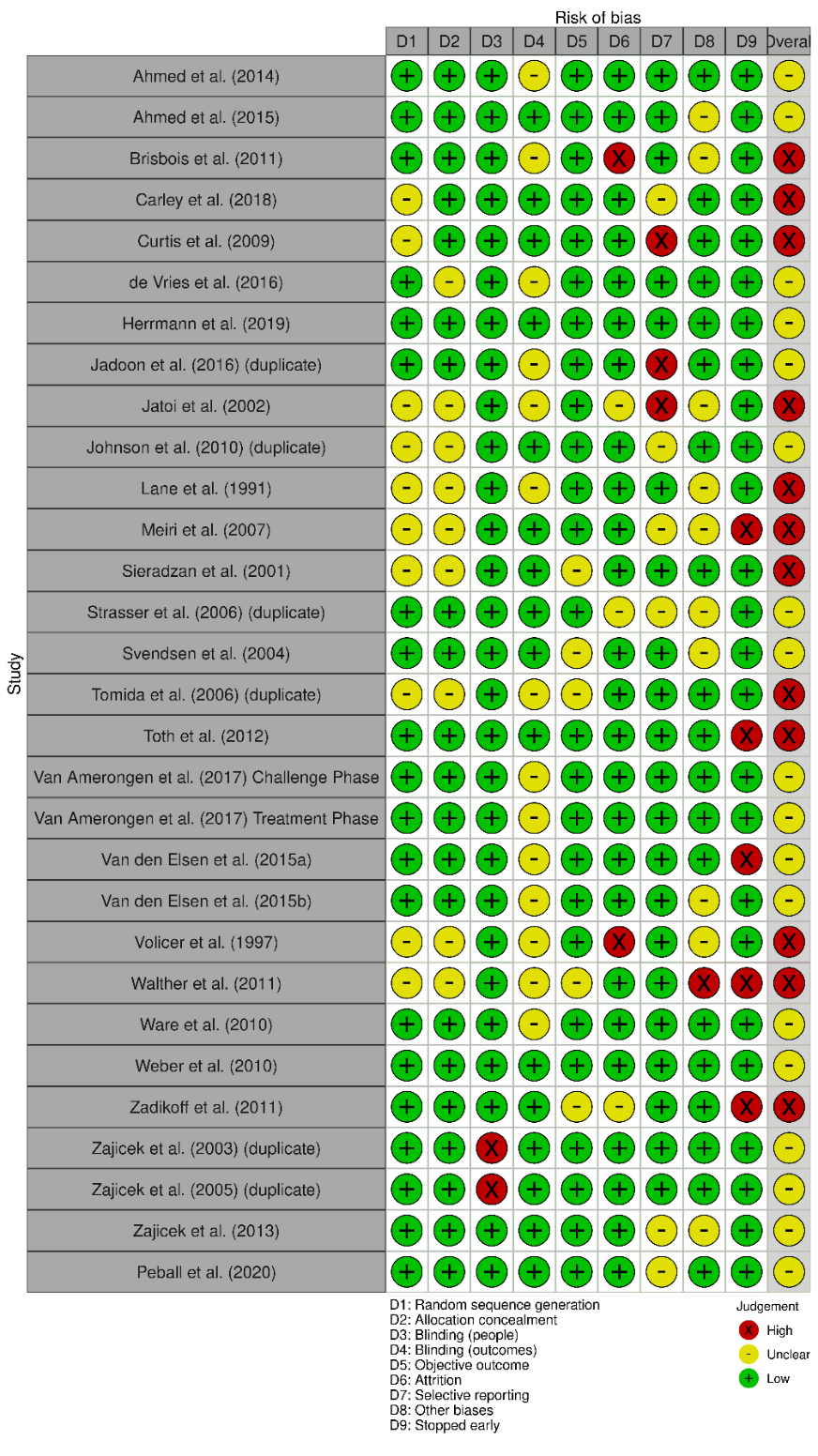


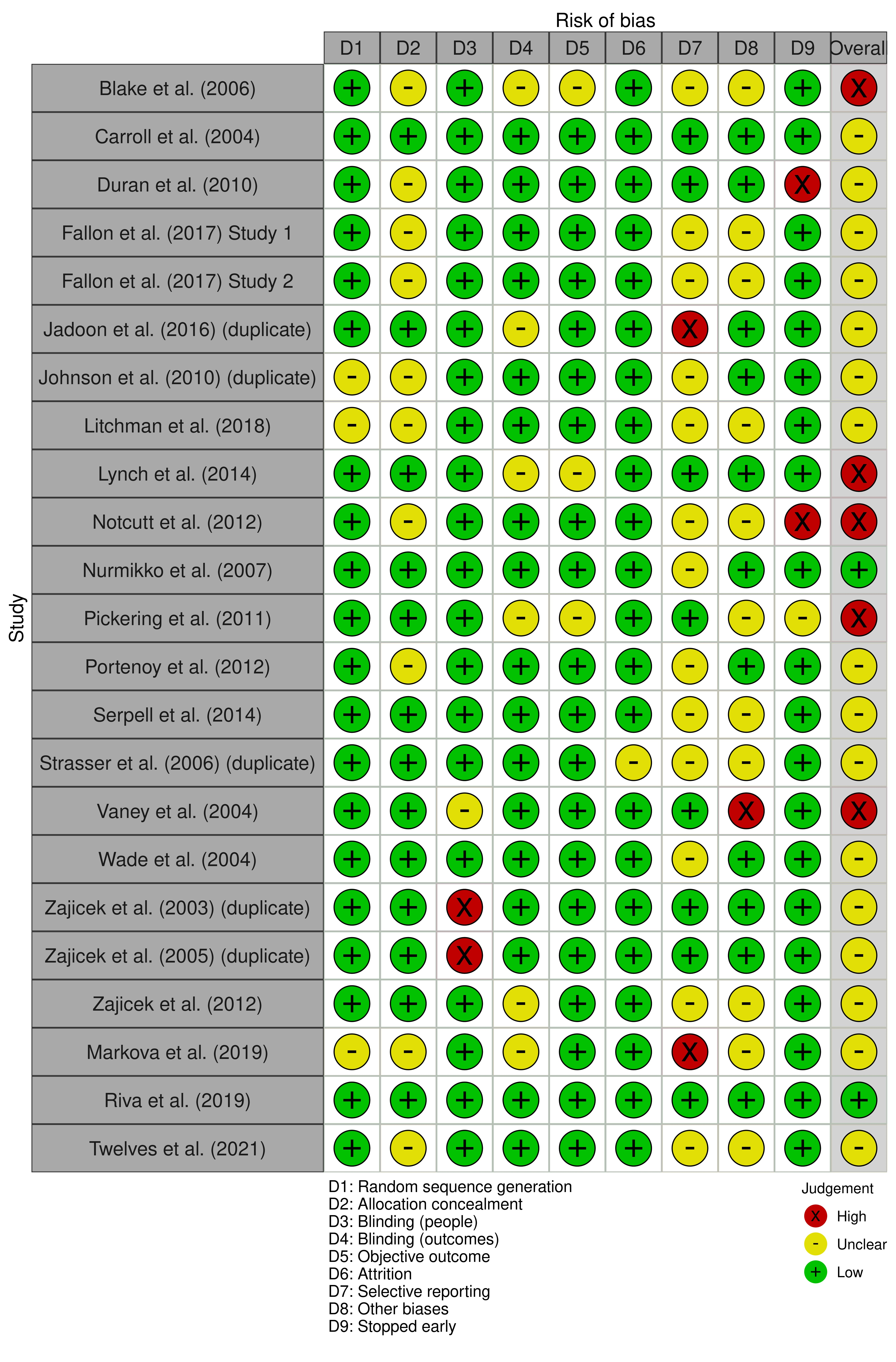


###
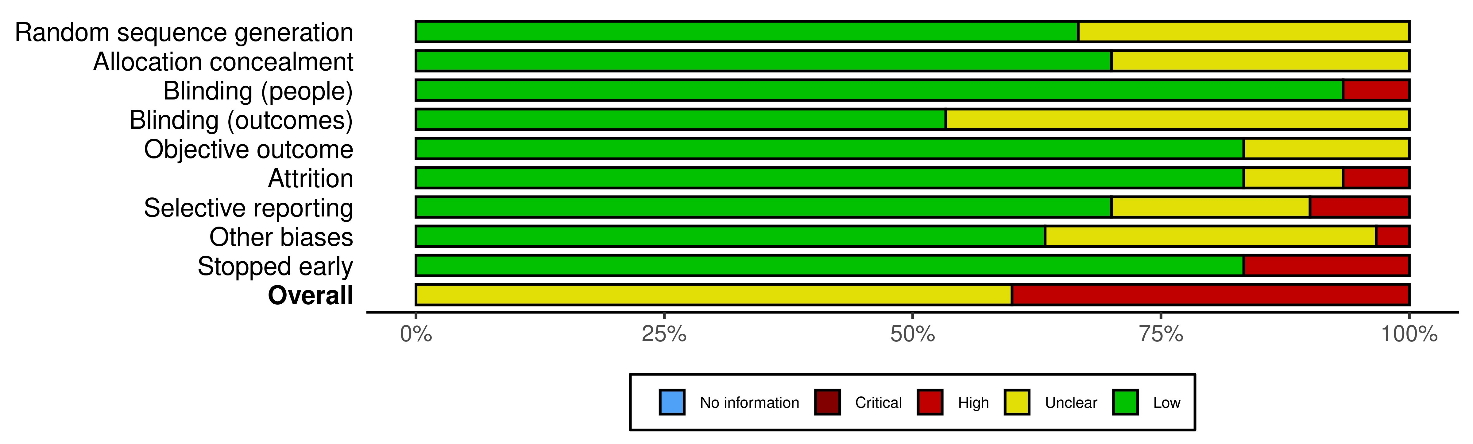
Figure 15b. Overall summary plot showing quality ratings per domain across studies for THC studies.


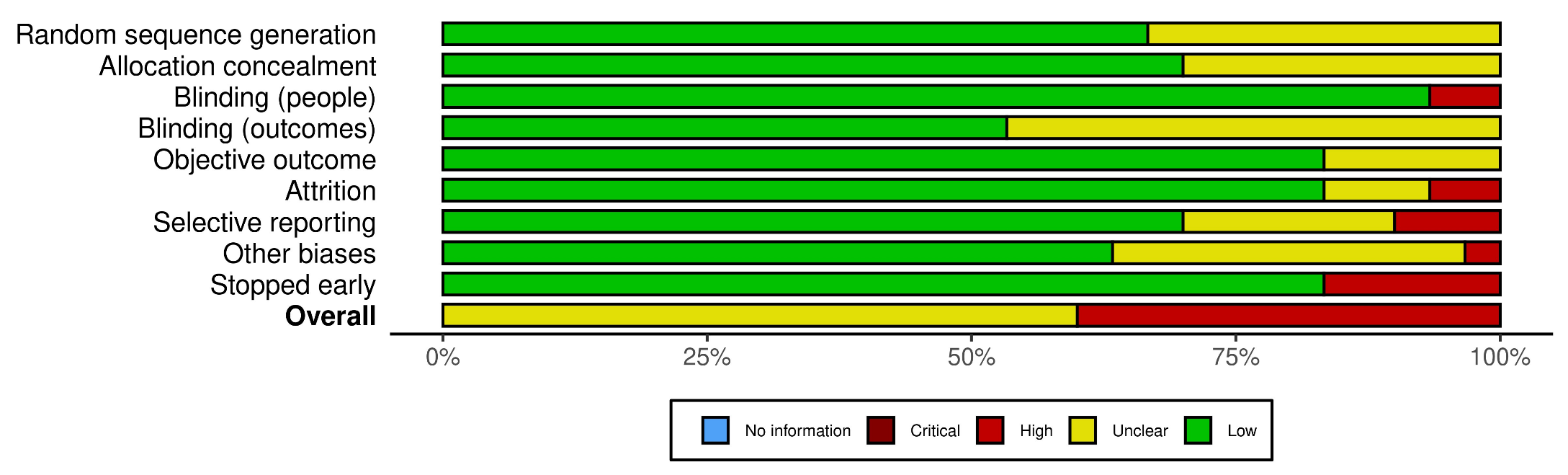


### Figure 16a. Traffic lights plot showing quality ratings for THC:CBD studies


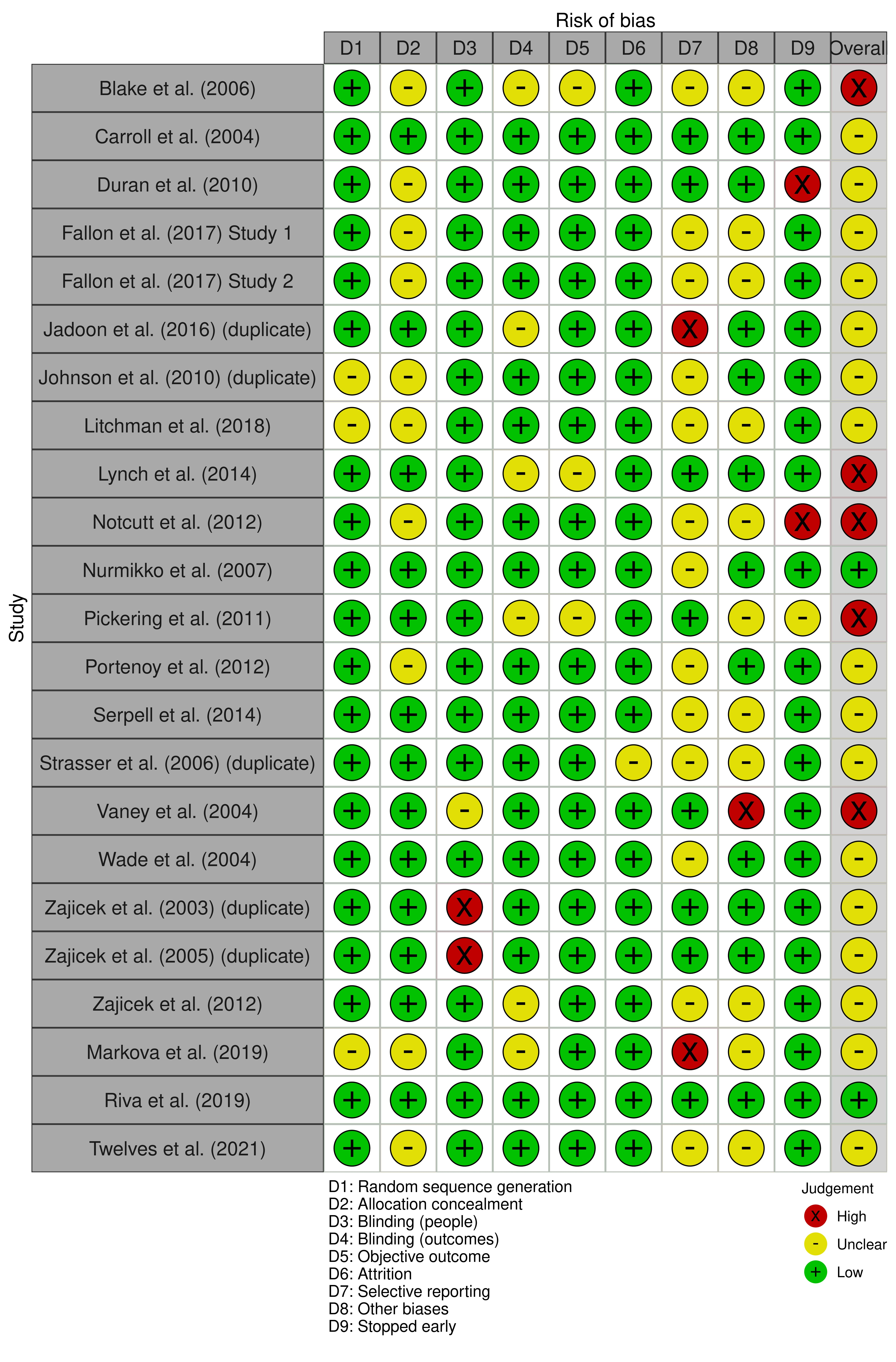


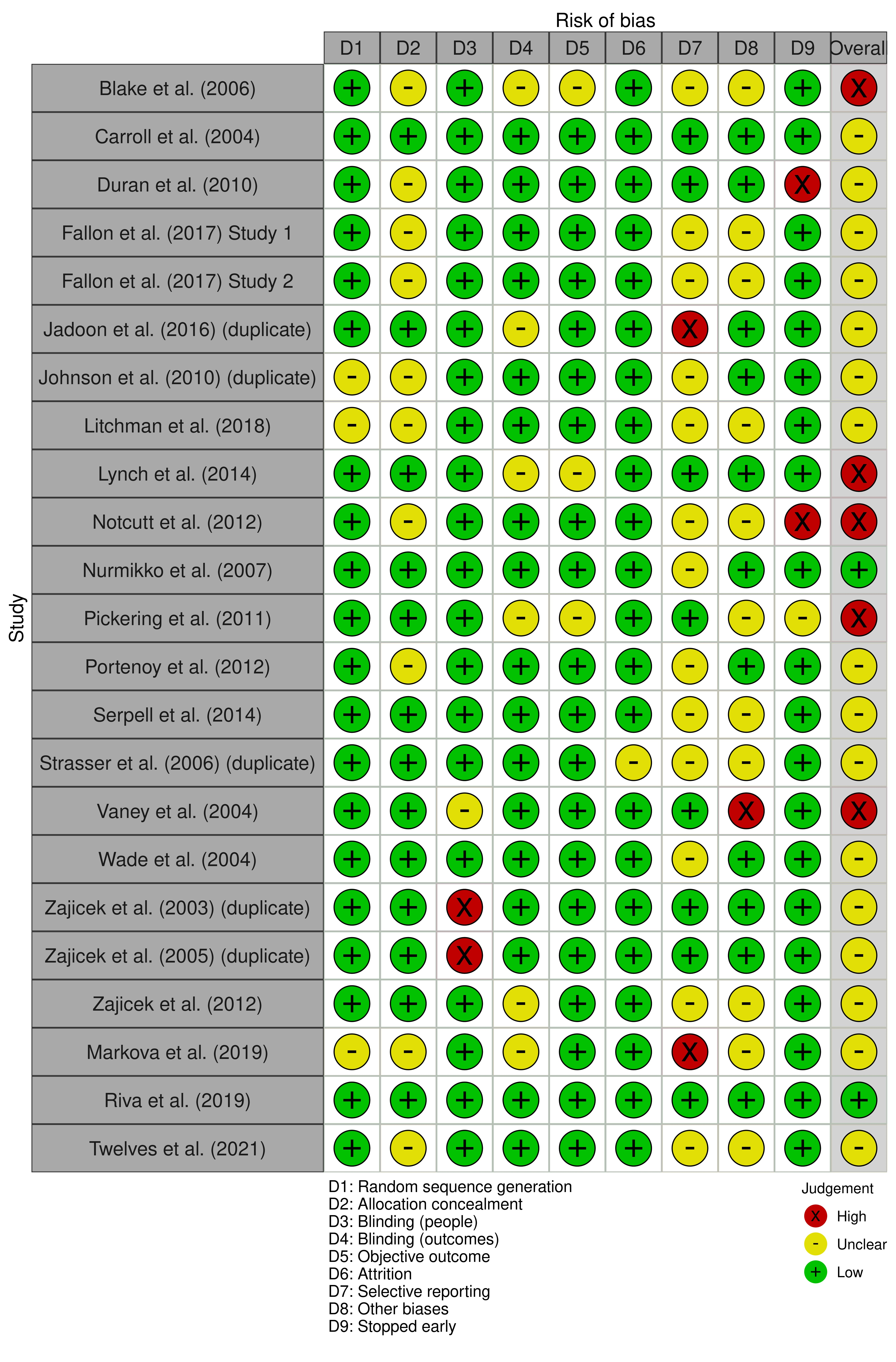


### Figure 16b. Overall summary plot showing quality ratings per domain across studies for THC:CBD studies


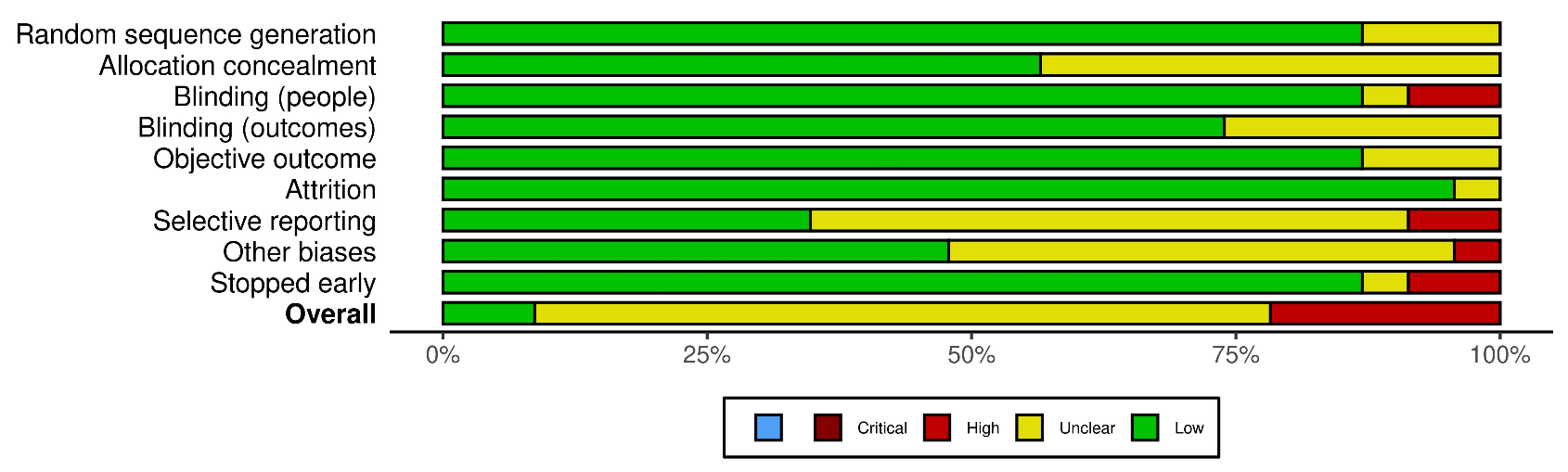


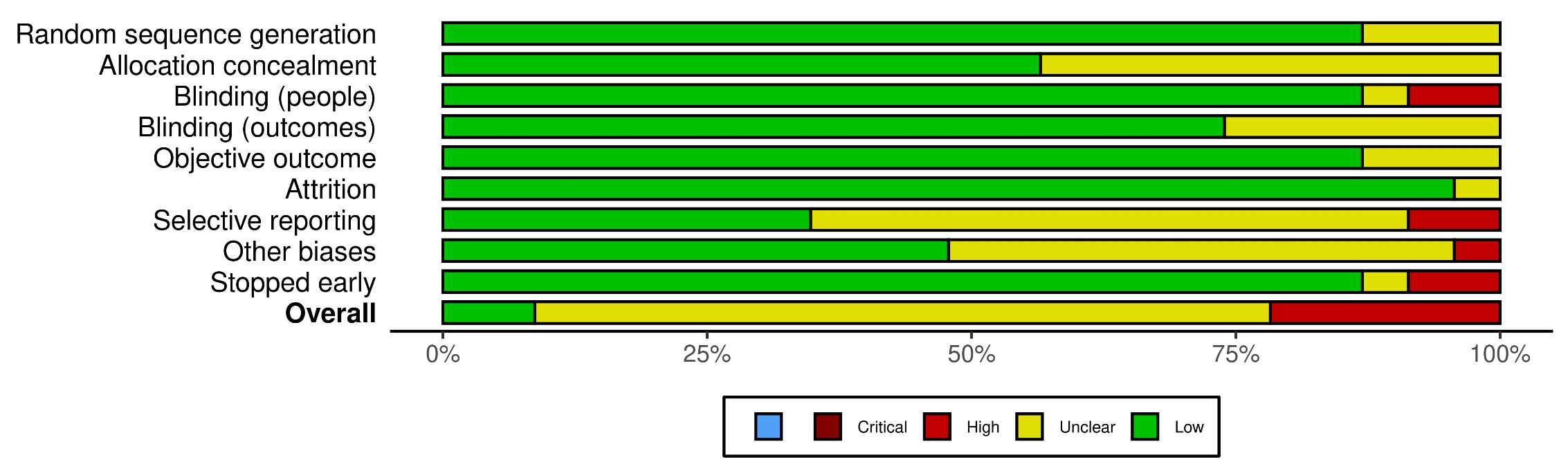


## Publication bias – funnel plots

**Funnel plots from the meta-analyses are reported for all-cause and treatment-related events (i.e., adverse events, serious adverse events, withdrawals, and deaths), and for the overall and subgroups analyses (i.e., overall, crossover, RCT studies).**

THC studies

### Figure 17a. Funnel plots for all-cause adverse events (AEs) meta-analyses for THC studies


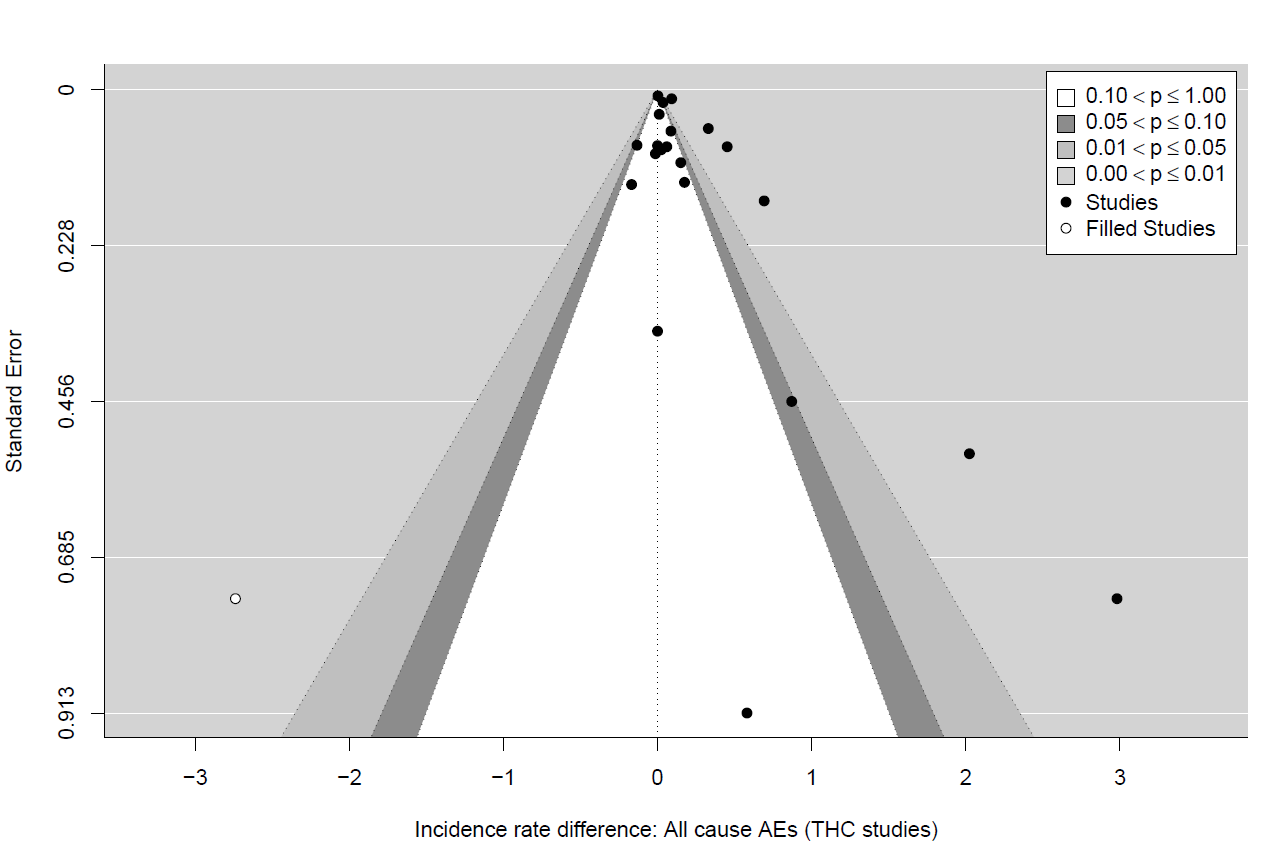


### Figure 17b. Funnel plots for all-cause adverse events (AEs) meta-analyses for THC crossover studies


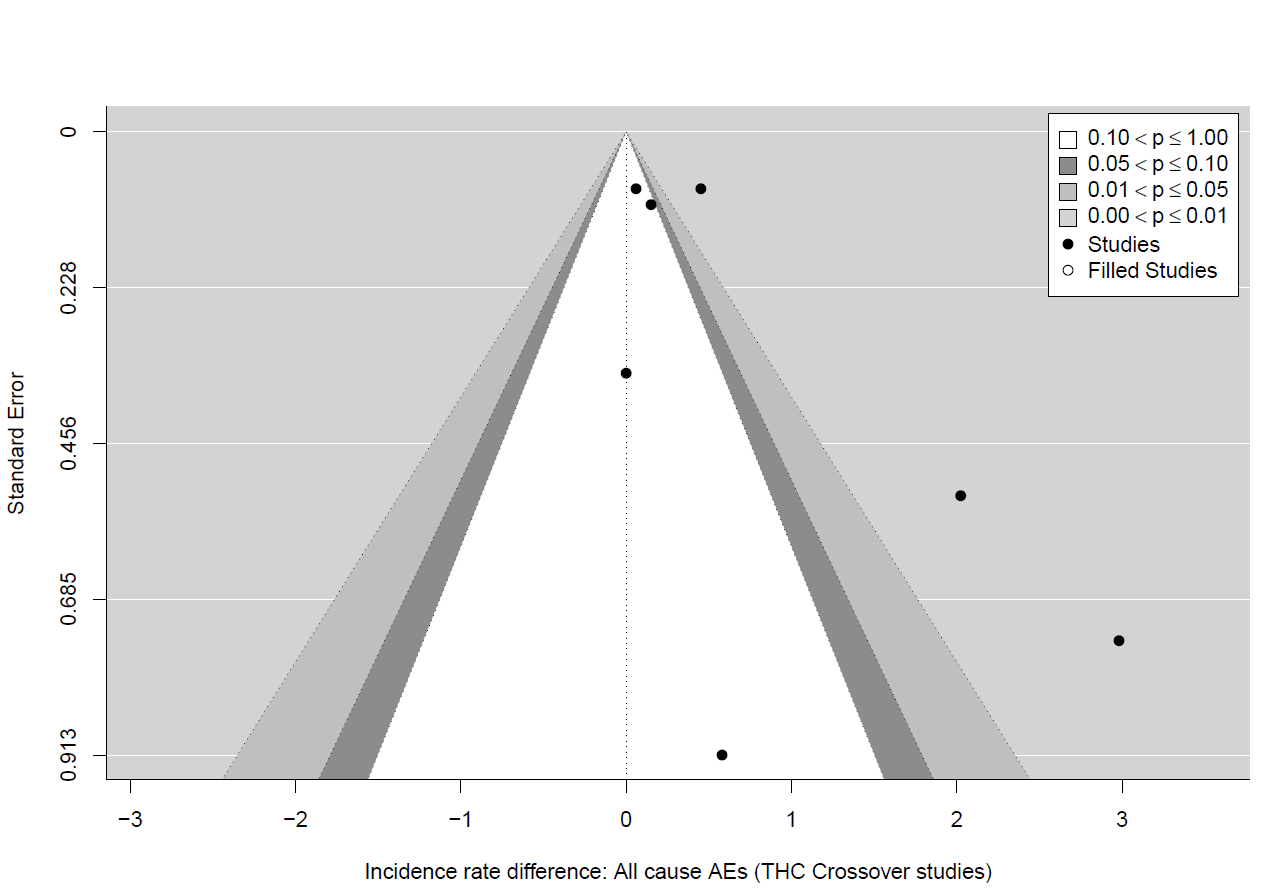


### Figure 17c. Funnel plots for all-cause adverse events (AEs) meta-analyses for THC RCT studies


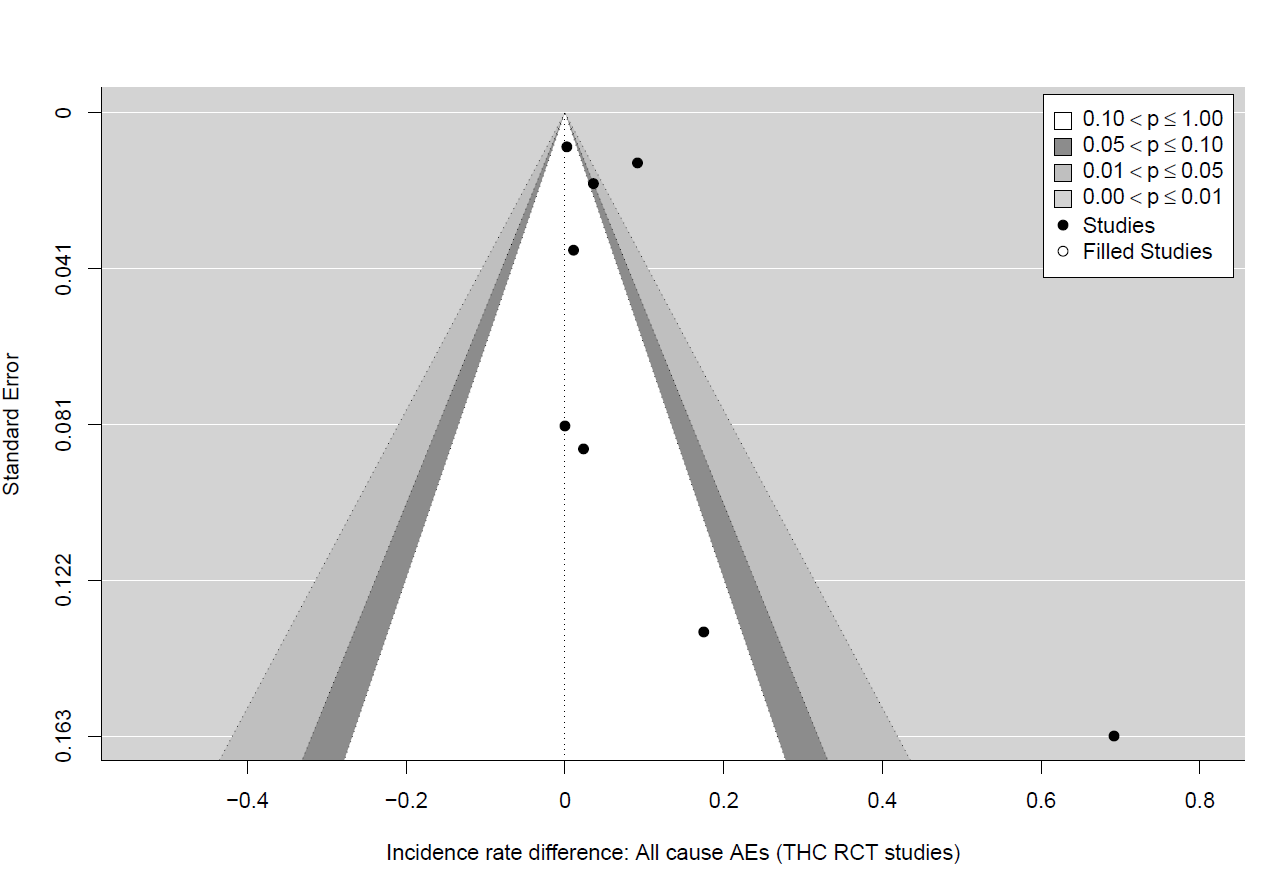


### Figure 17d. Funnel plots for treatment-related adverse events (AEs) meta-analyses for THC studies


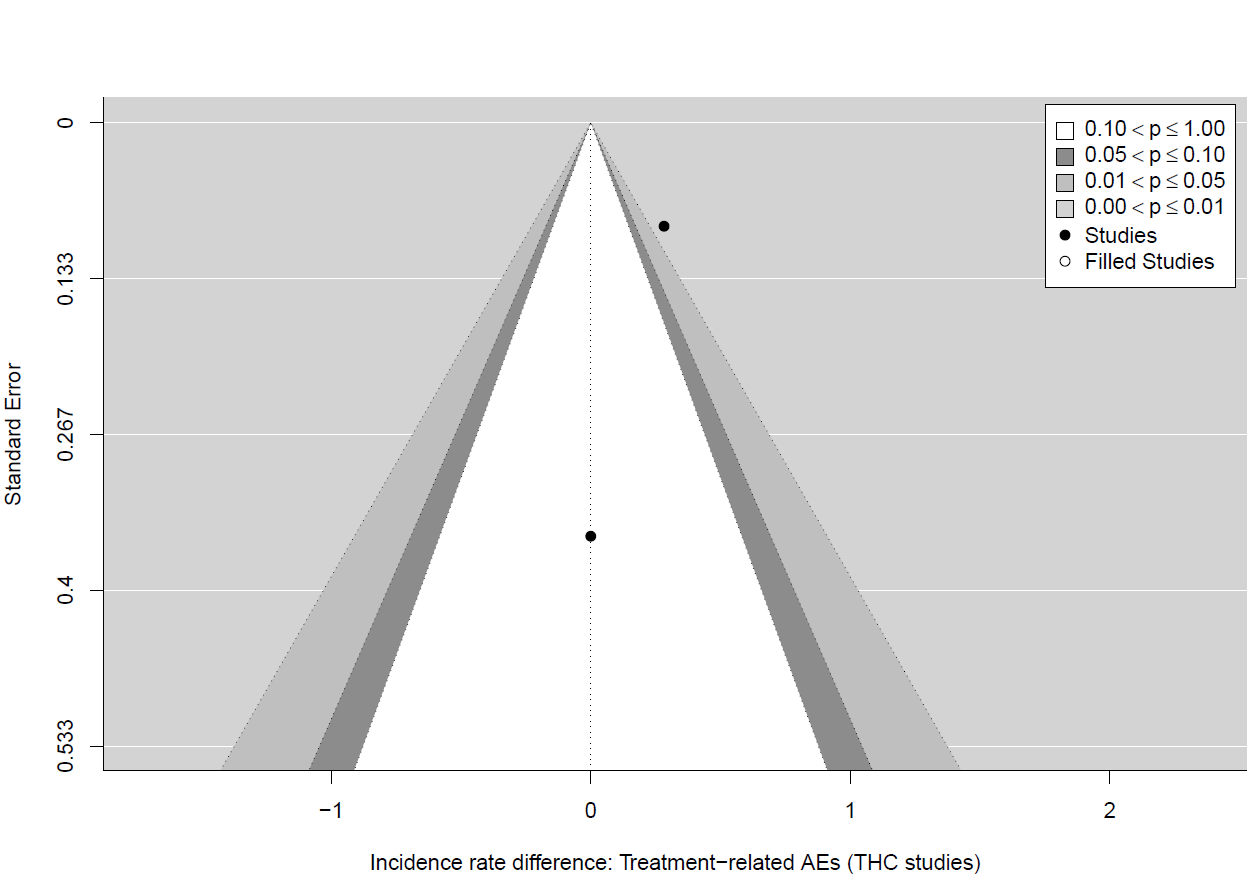


### Figure 17e. Funnel plots for treatment-related adverse events (AEs) meta-analyses for THC crossover studies


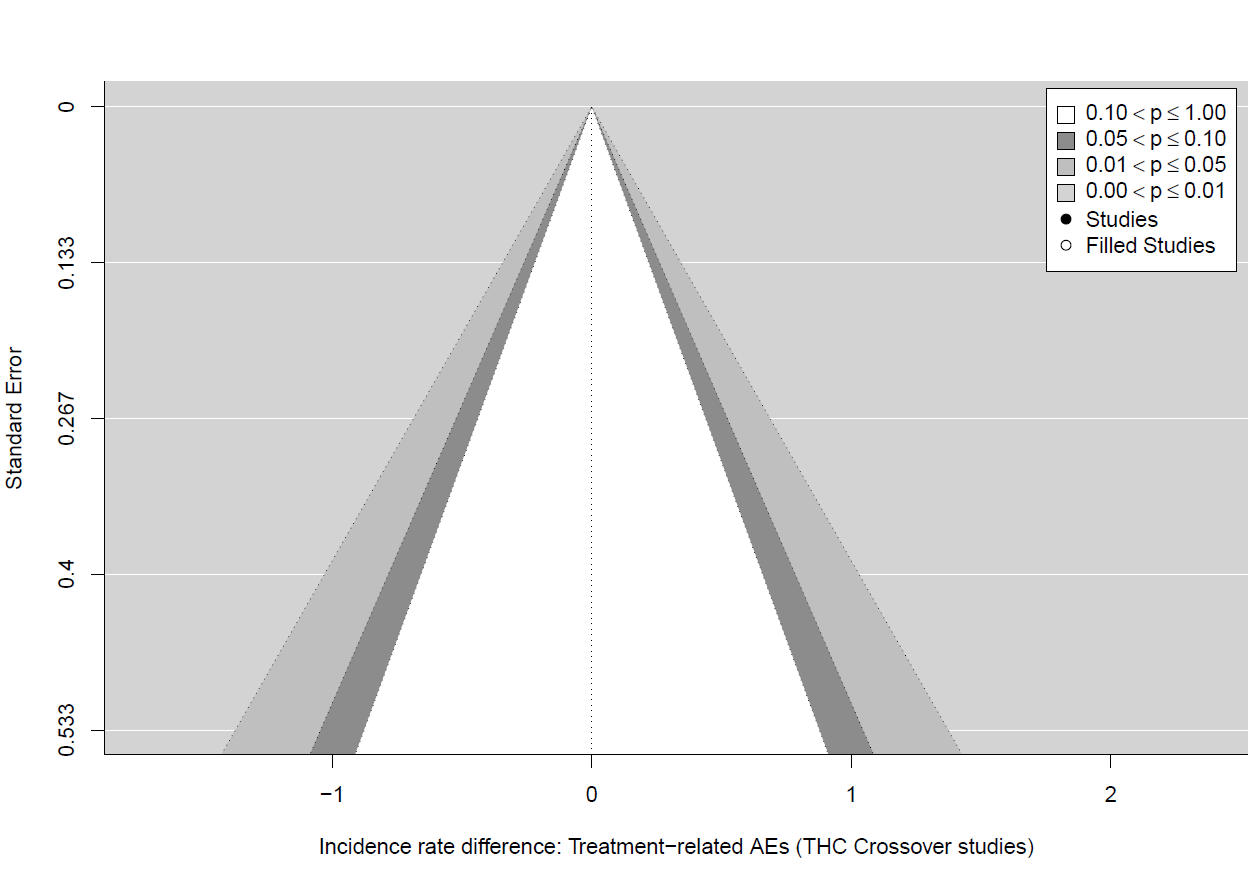


### Figure 17f. Funnel plots for treatment-related adverse events (AEs) meta-analyses for THC RCT studies


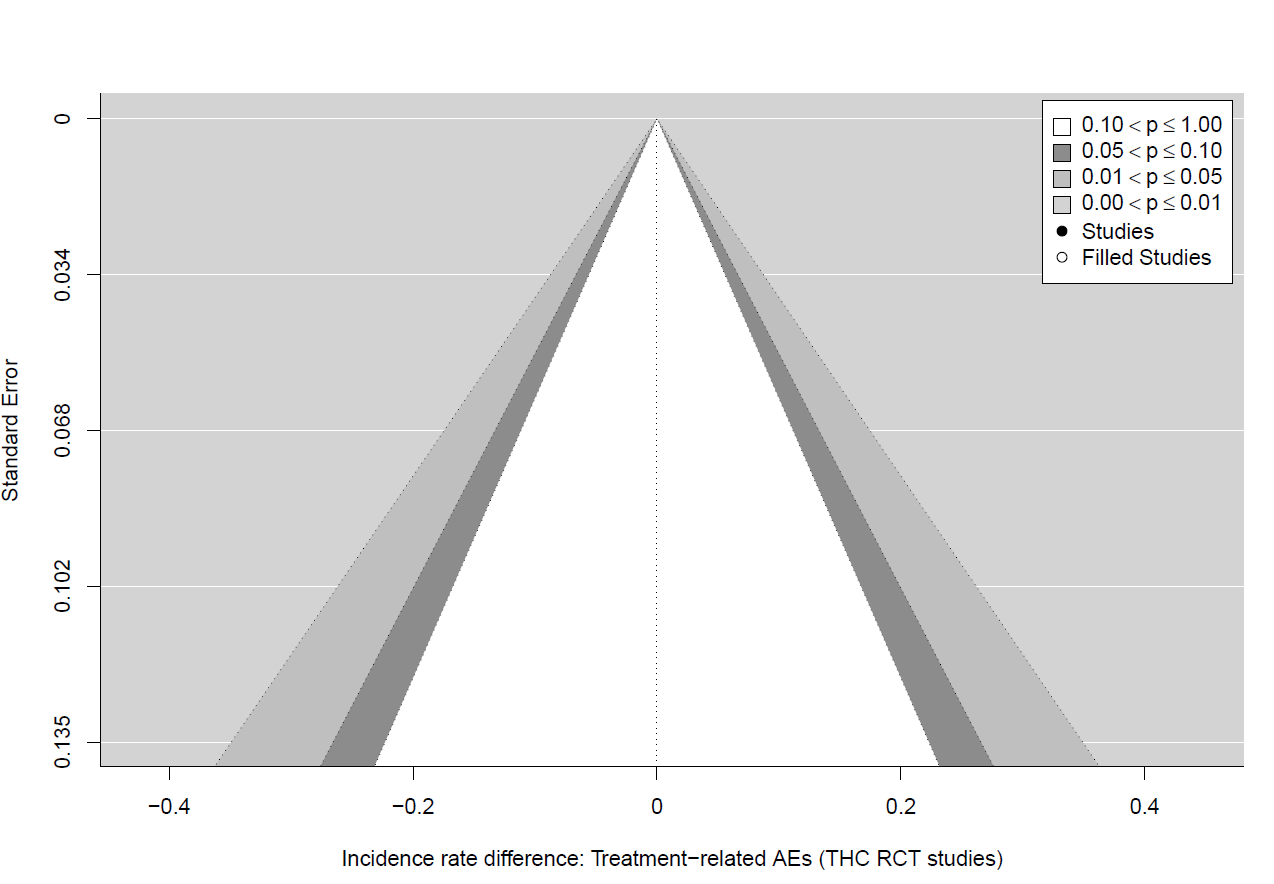


### Figure 18a. Funnel plots for all-cause serious adverse events (SAEs) meta-analyses for THC studies.


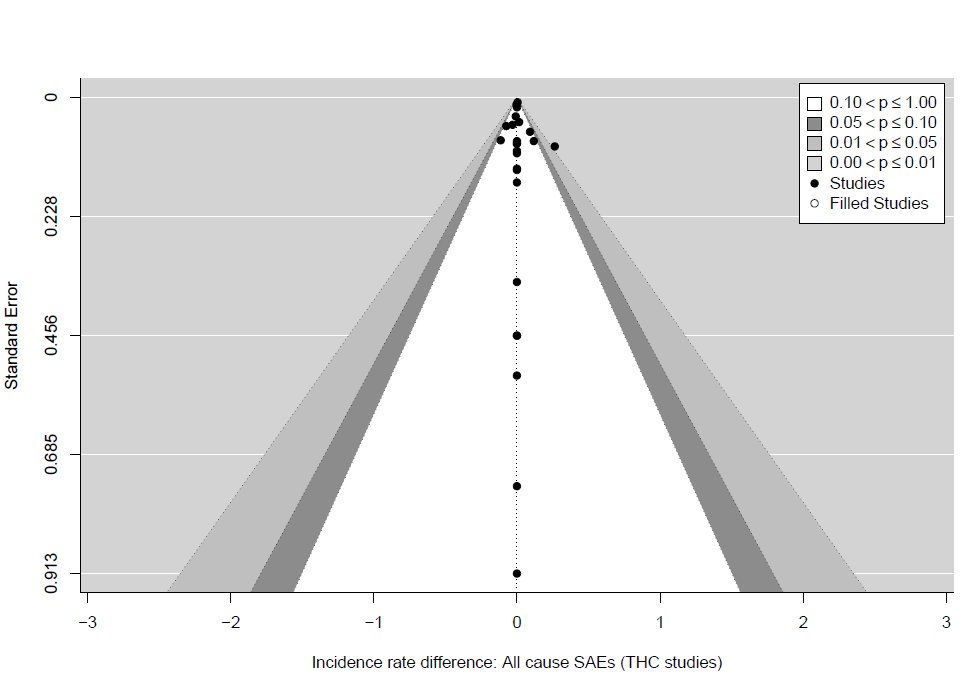


### Figure 18b. Funnel plots for all-cause serious adverse events (SAEs) meta-analyses for THC crossover studies


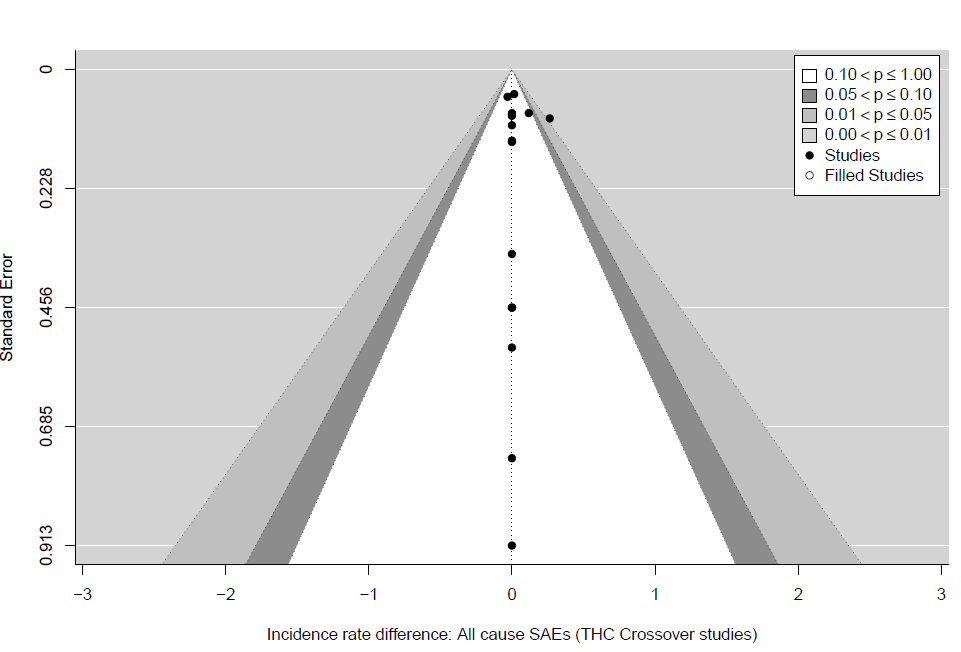


### Figure 18c. Funnel plots for all-cause serious adverse events (SAEs) meta-analyses for THC RCT studies


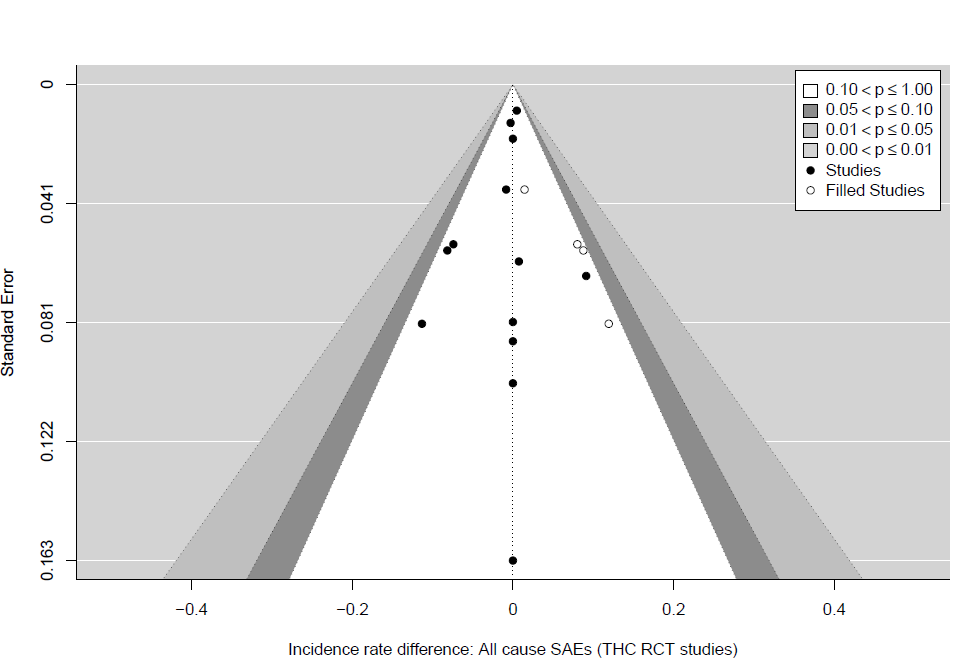


### Figure 18d. Funnel plots for treatment-related serious adverse events (SAEs) meta-analyses for THC studies


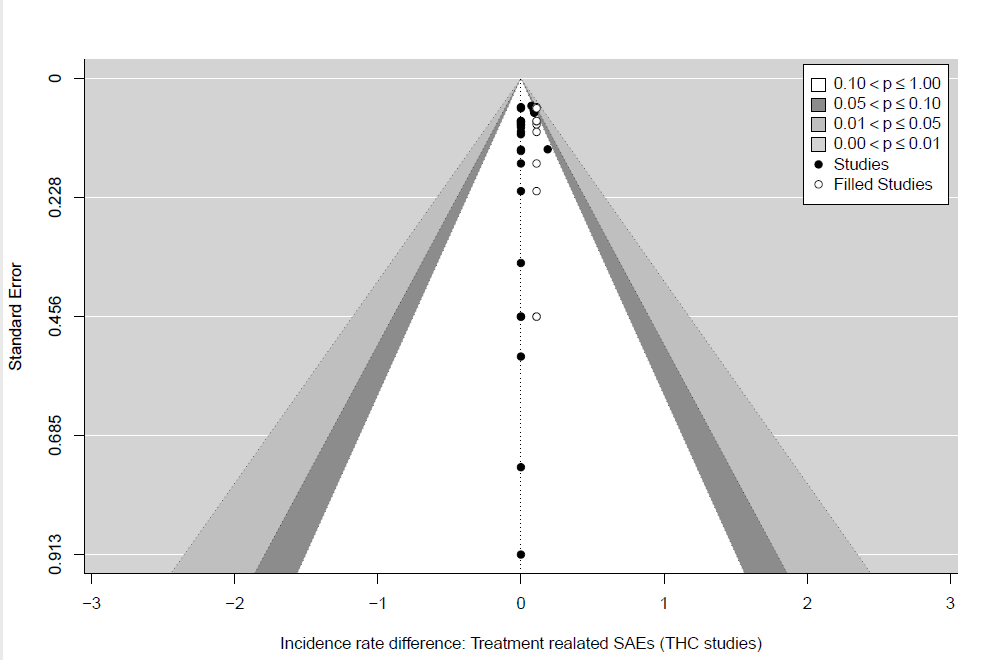


### Figure 18e. Funnel plots for treatment-related serious adverse events (SAEs) meta-analyses for THC crossover studies


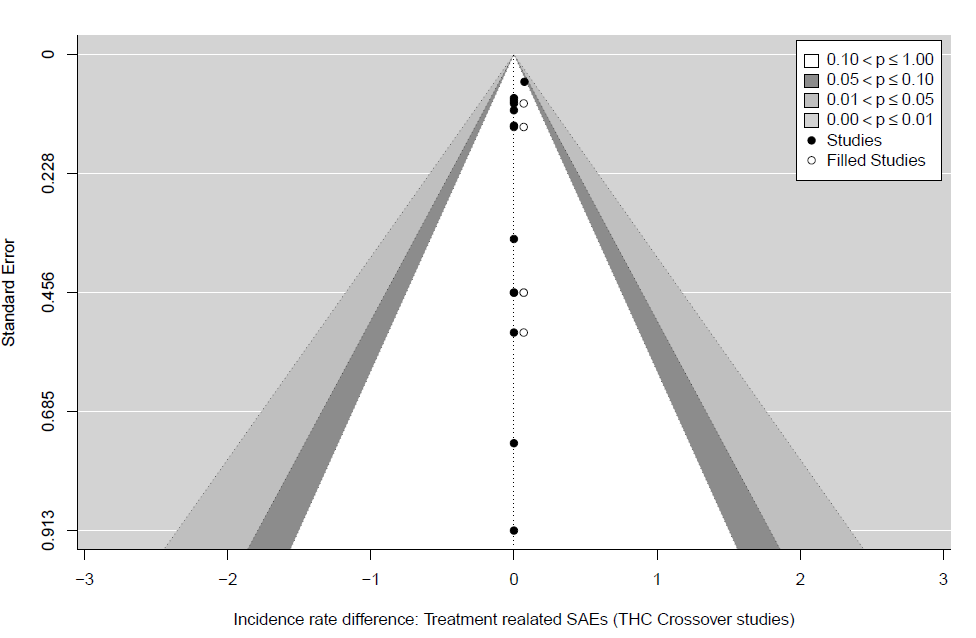


### Figure 18f. Funnel plots for treatment-related serious adverse events (SAEs) meta-analyses for THC RCT studies


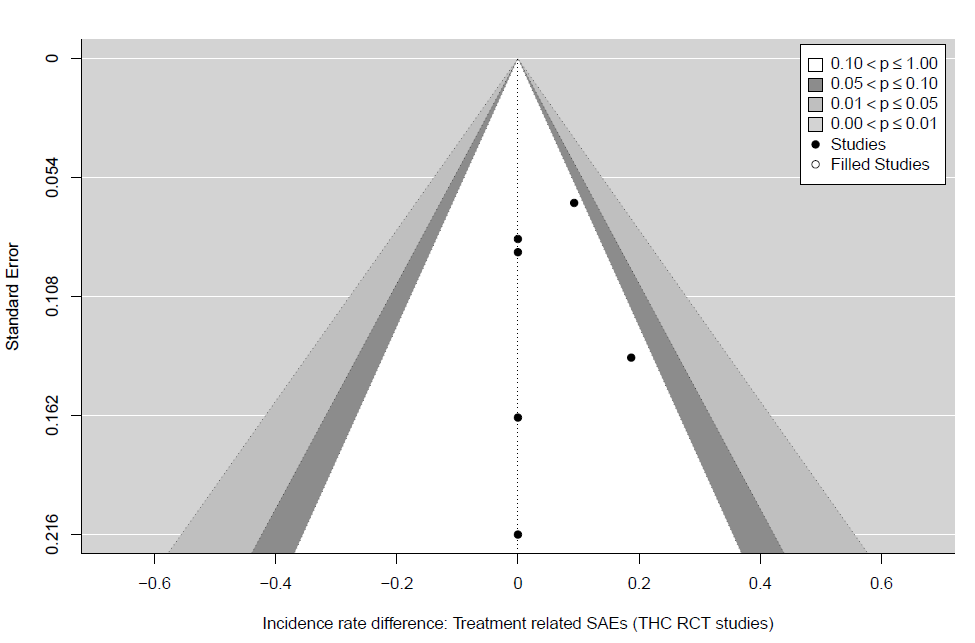


### Figure 19a. Funnel plots for all-cause withdrawals meta-analyses for THC studies


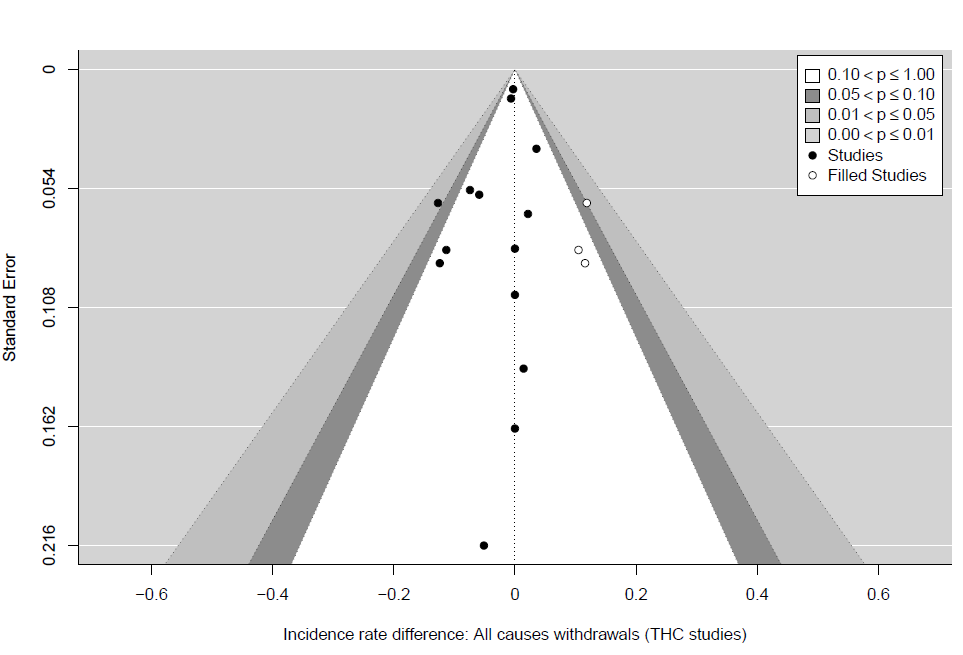


### Figure 19b. Funnel plots for all-cause withdrawals meta-analyses for THC RCT studies


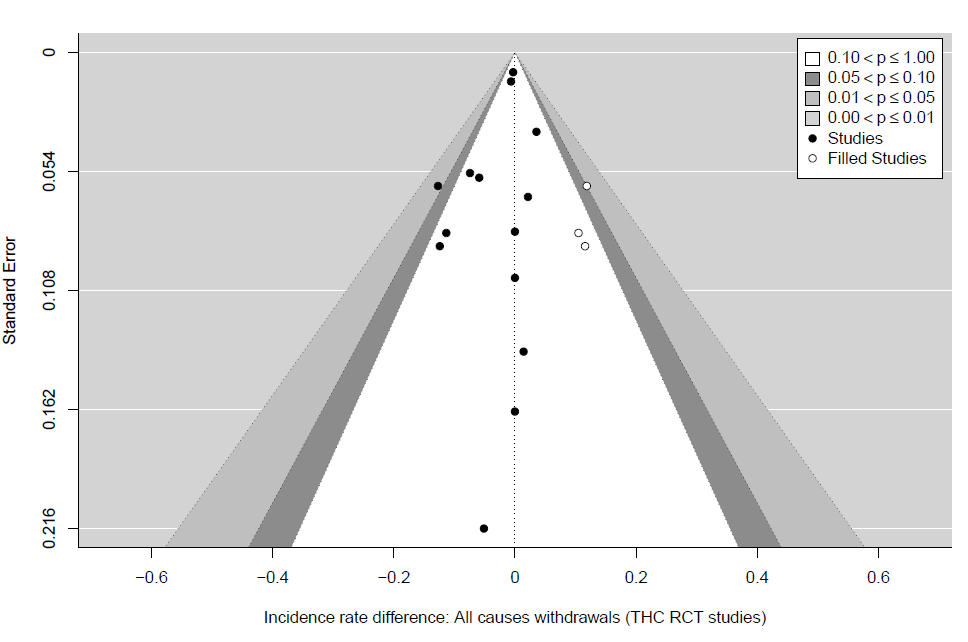


### Figure 19c. Funnel plots for treatment-related withdrawals meta-analyses for THC studies.


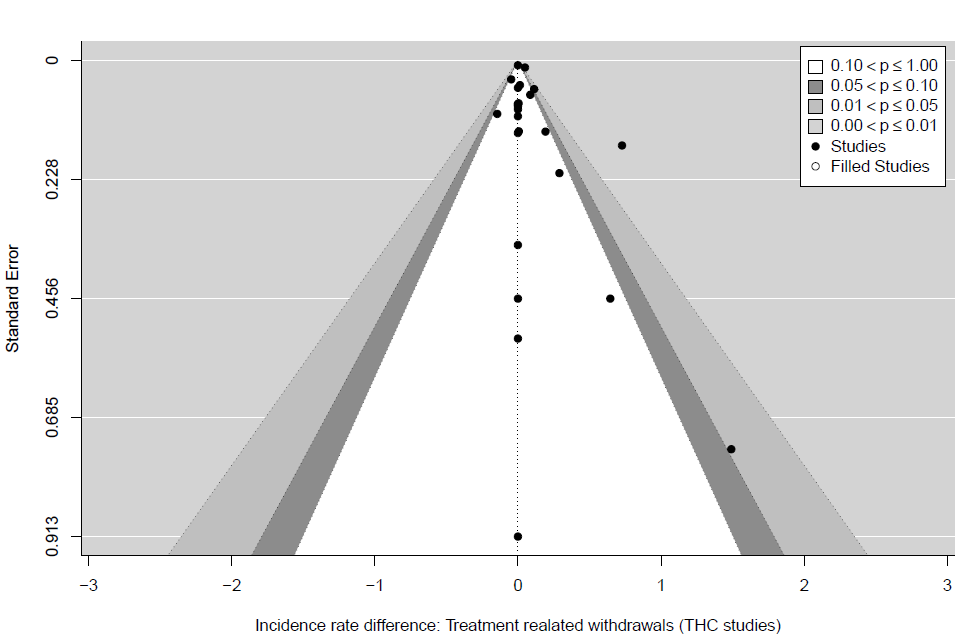


### Figure 19d. Funnel plots for treatment-related withdrawals meta-analyses for THC crossover studies


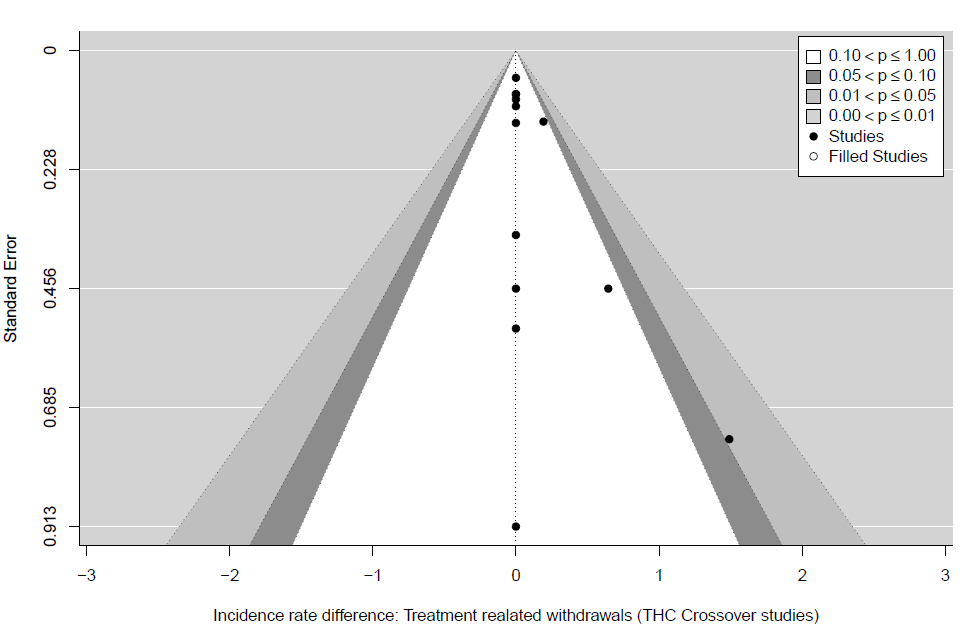


### Figure 19e. Funnel plots for treatment-related withdrawals meta-analyses for THC RCT studies


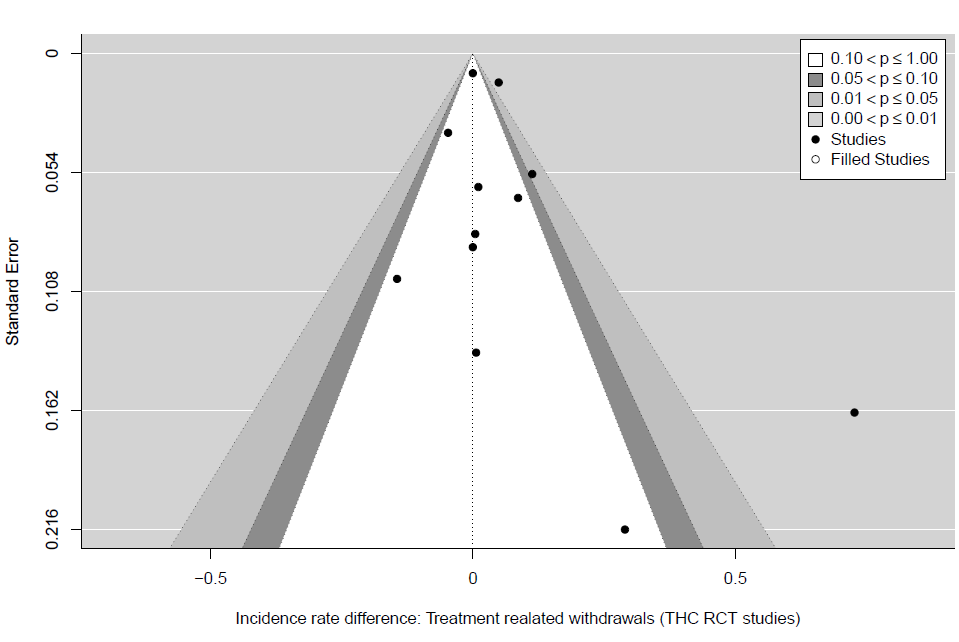


### Figure 20a. Funnel plots for deaths meta-analyses for THC studies


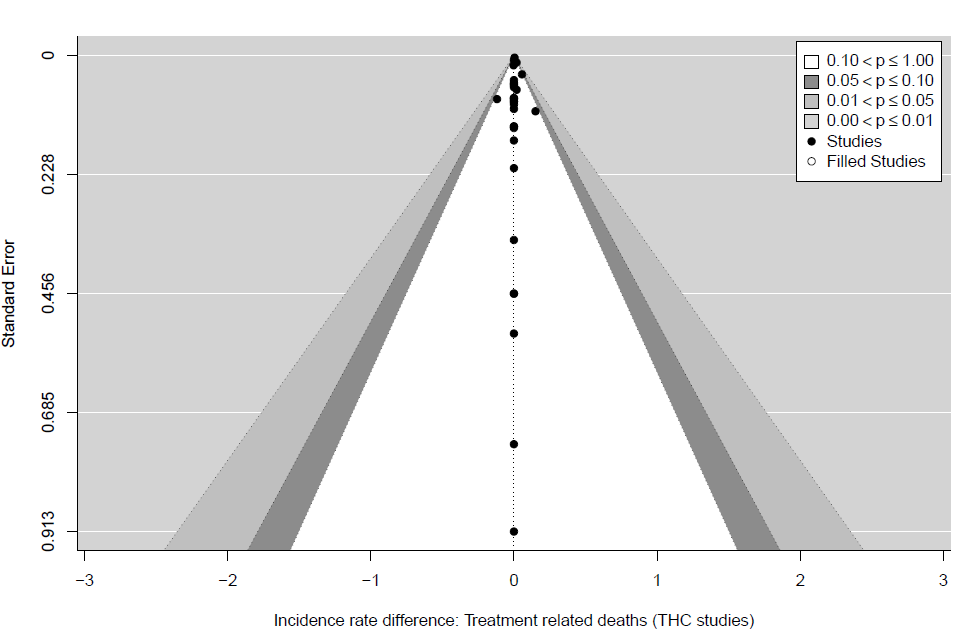


### Figure 20b. Funnel plots for deaths meta-analyses for THC crossover studies


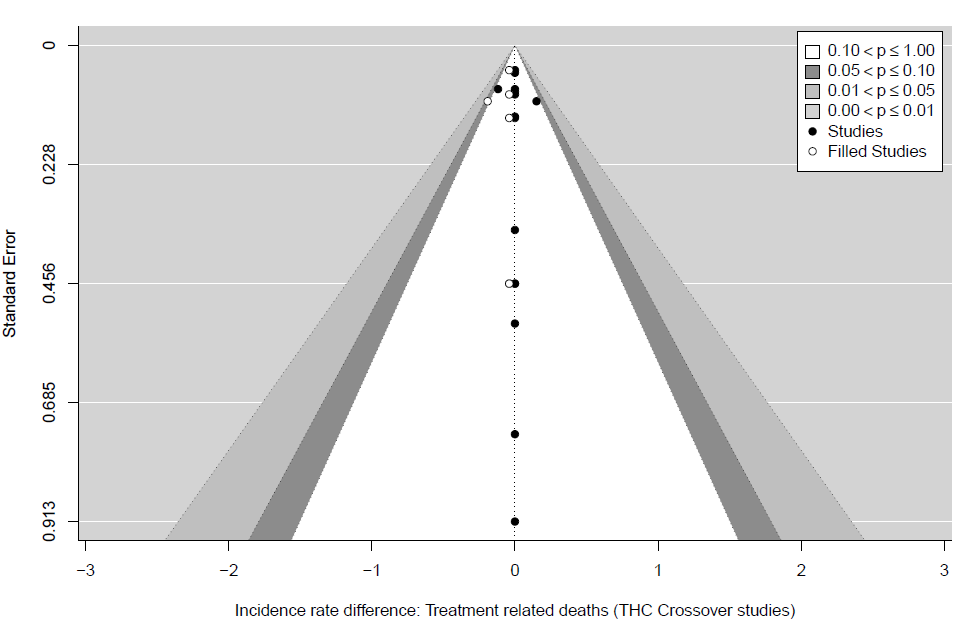


### Figure 20c. Funnel plots for deaths meta-analyses for THC RCT studies


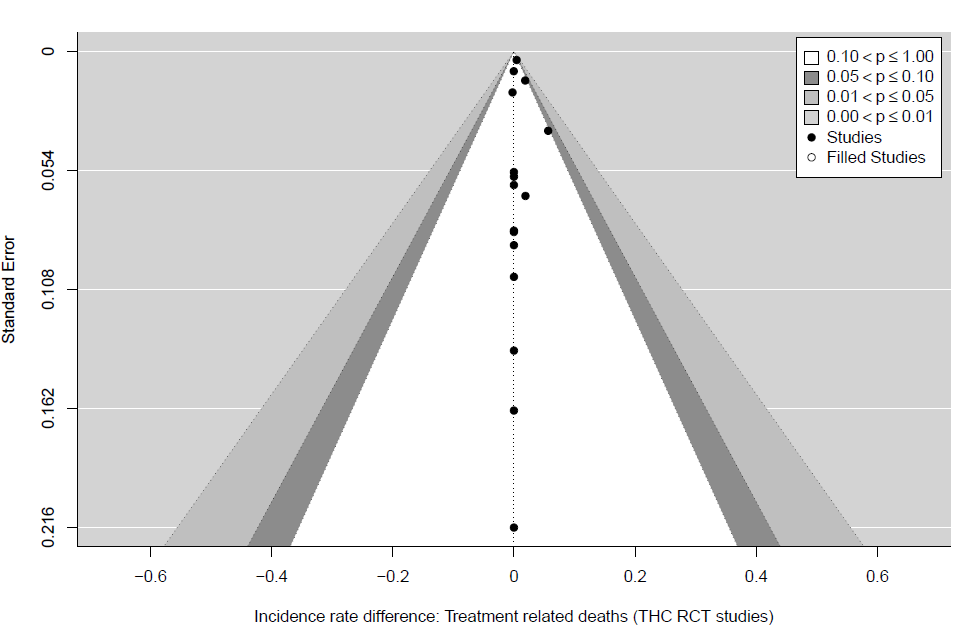


THC:CBD studies

### Figure 21a. Funnel plots for all-cause adverse events (AEs) meta-analyses for THC:CBD studies


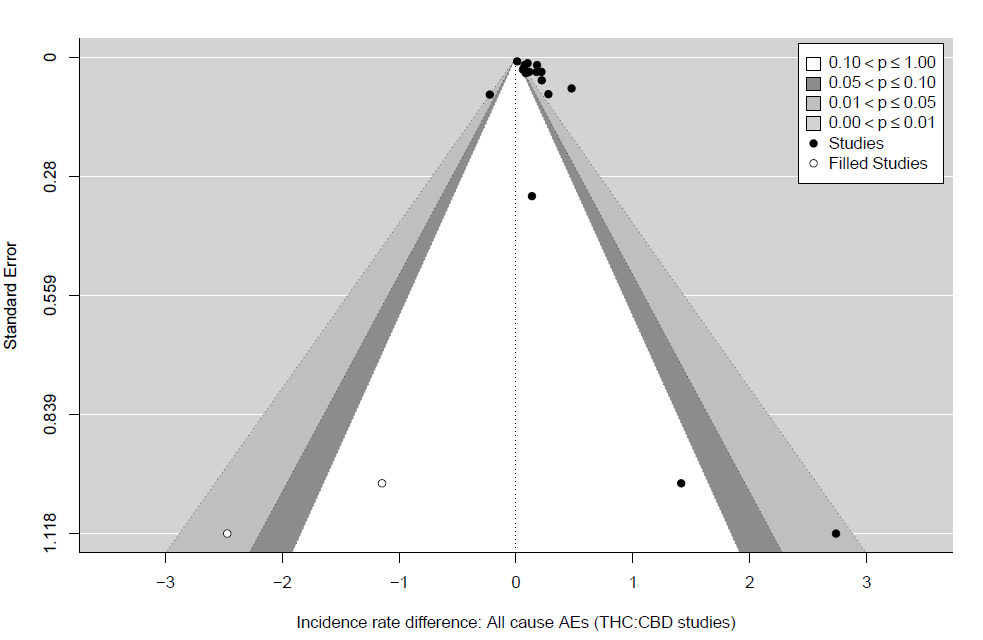


### Figure 21b. Funnel plots for all-cause adverse events (AEs) meta-analyses for THC:CBD crossover studies


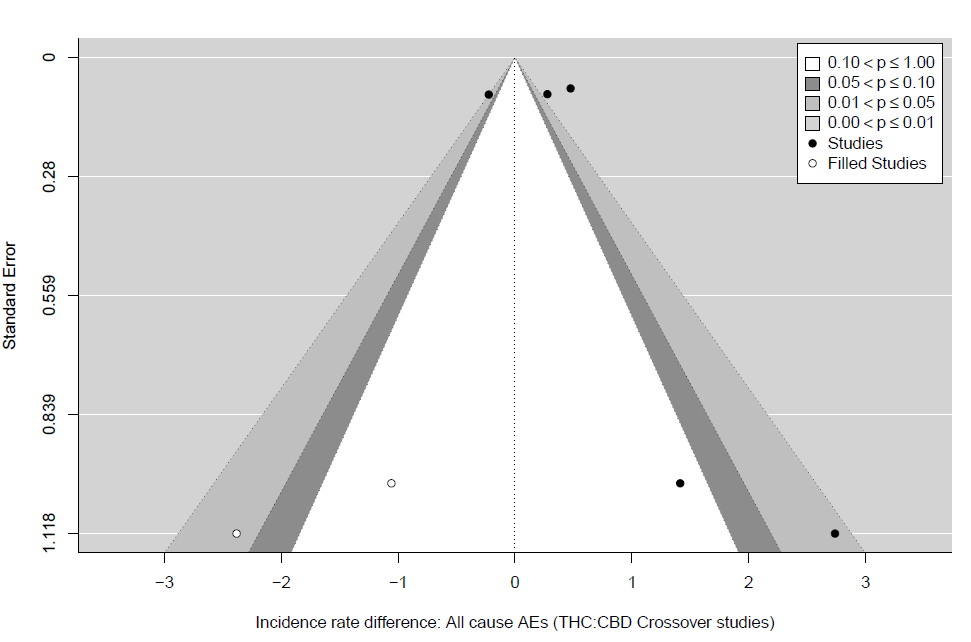


### Figure 21c. Funnel plots for all-cause adverse events (AEs) meta-analyses for THC:CBD RCT studies


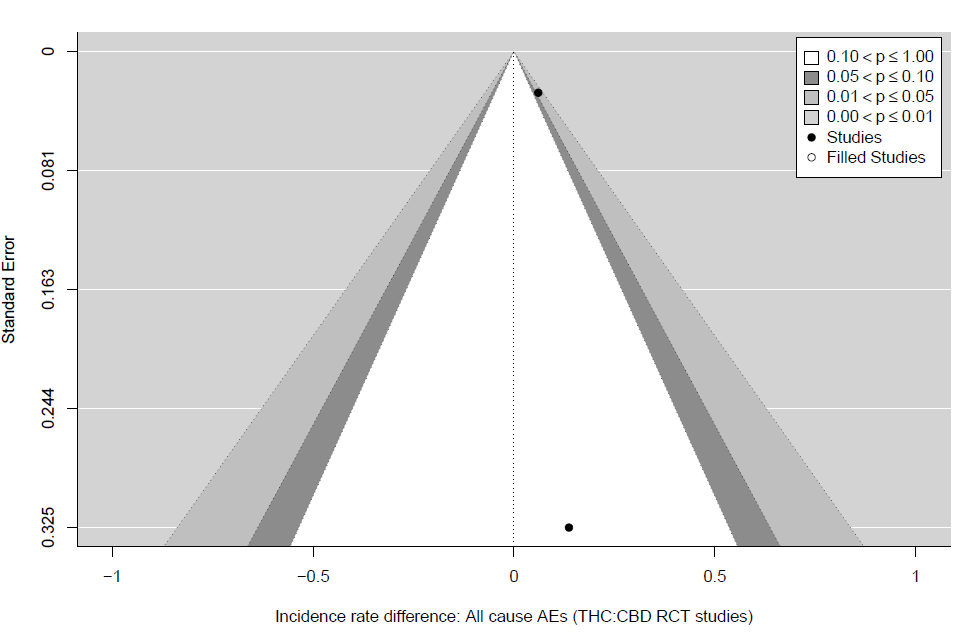


### Figure 21d. Funnel plots for treatment-related adverse events (AEs) meta-analyses for THC:CBD RCT studies


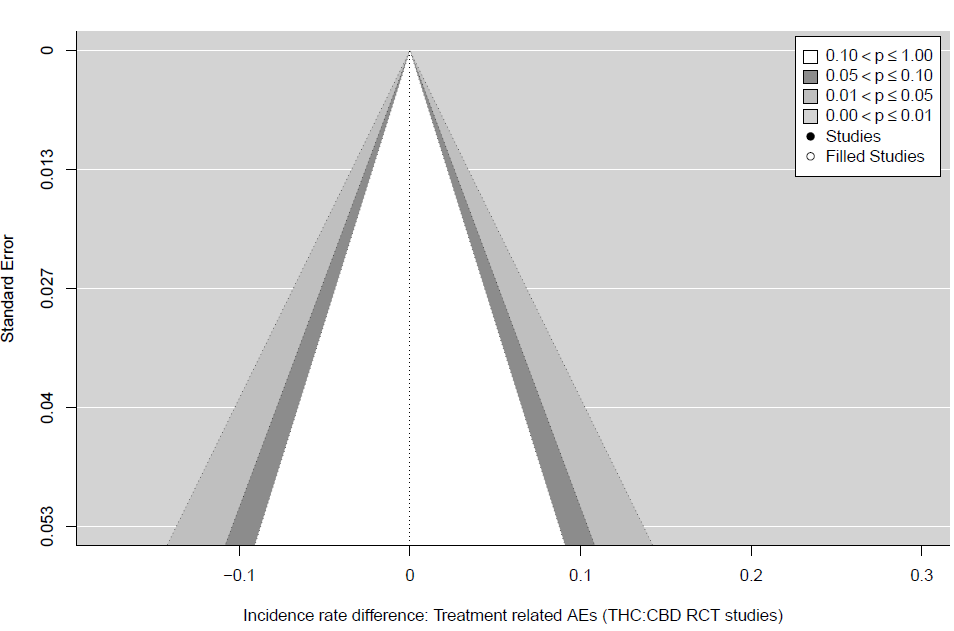


### Figure 22a. Funnel plots for all-cause serious adverse events (SAEs) meta-analyses for THC:CBD studies


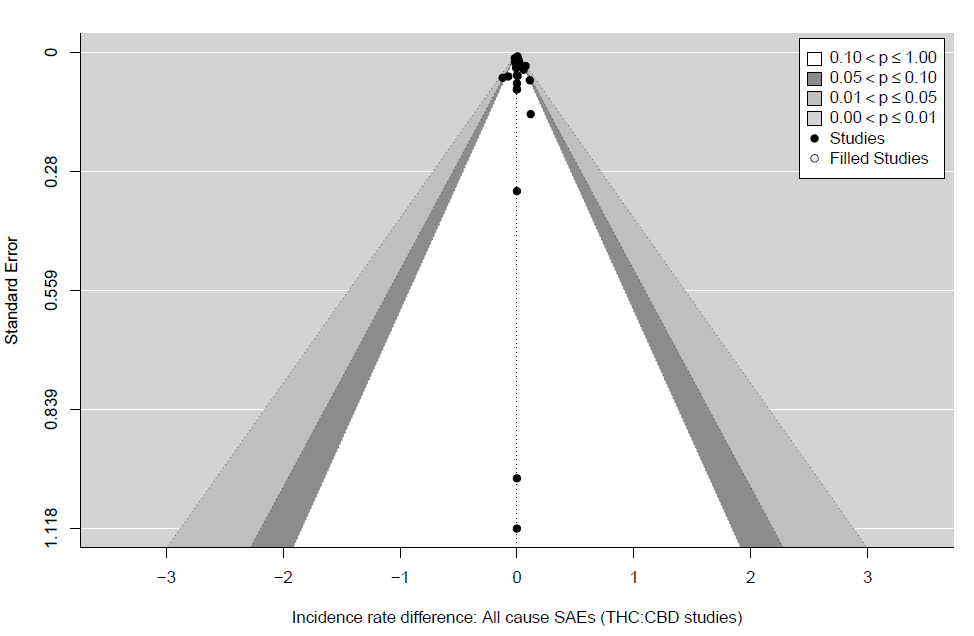


### Figure 22b. Funnel plots for all-cause serious adverse events (SAEs) meta-analyses for THC:CBD crossover studies


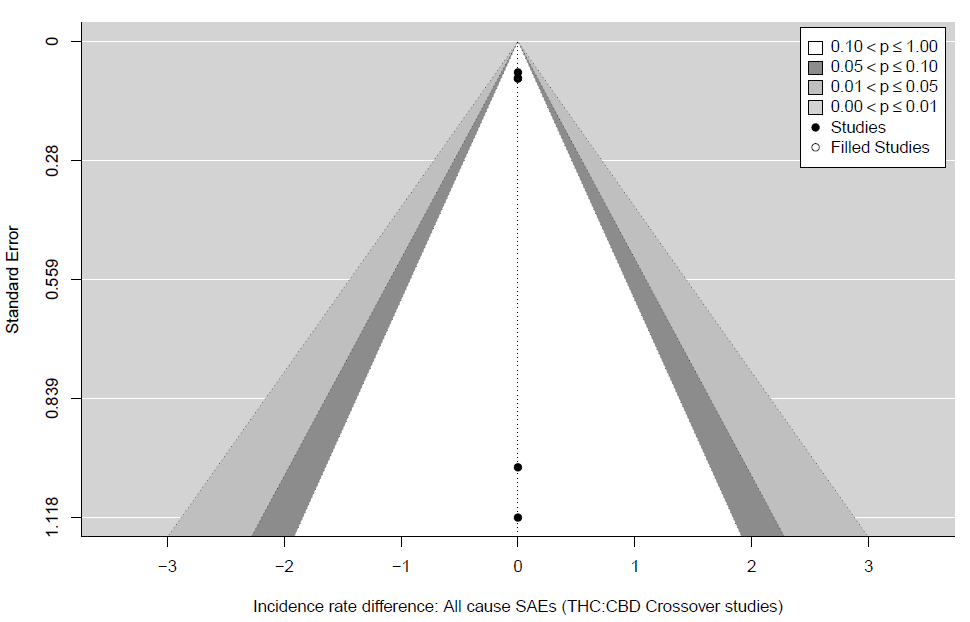


### Figure 22c. Funnel plots for all-cause serious adverse events (SAEs) meta-analyses for THC:CBD RCT studies


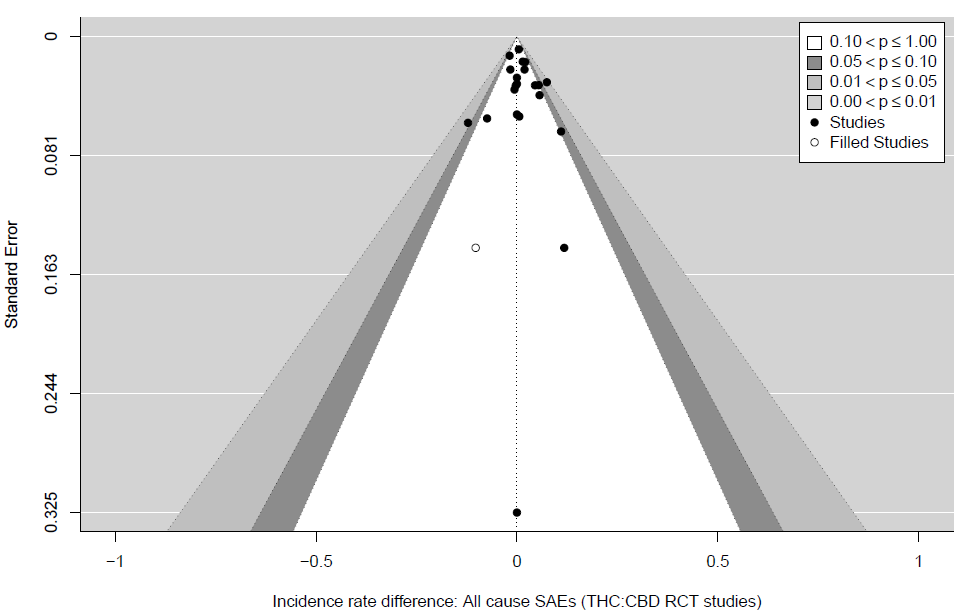


### Figure 22d. Funnel plots for treatment-related serious adverse events (SAEs) meta-analyses for THC:CBD studies


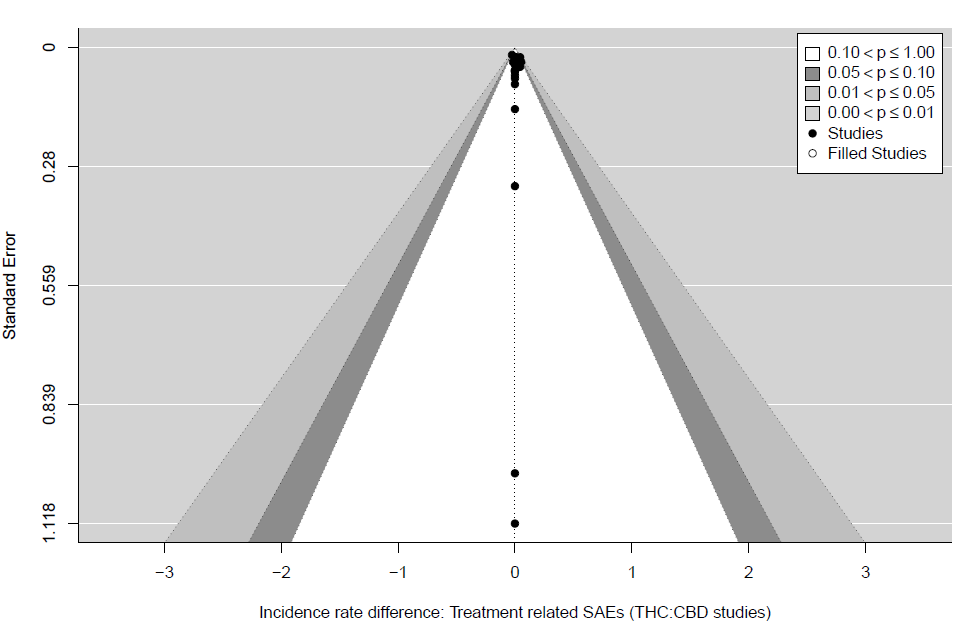


### Figure 22e. Funnel plots for treatment-related serious adverse events (SAEs) meta-analyses for THC:CBD crossover studies


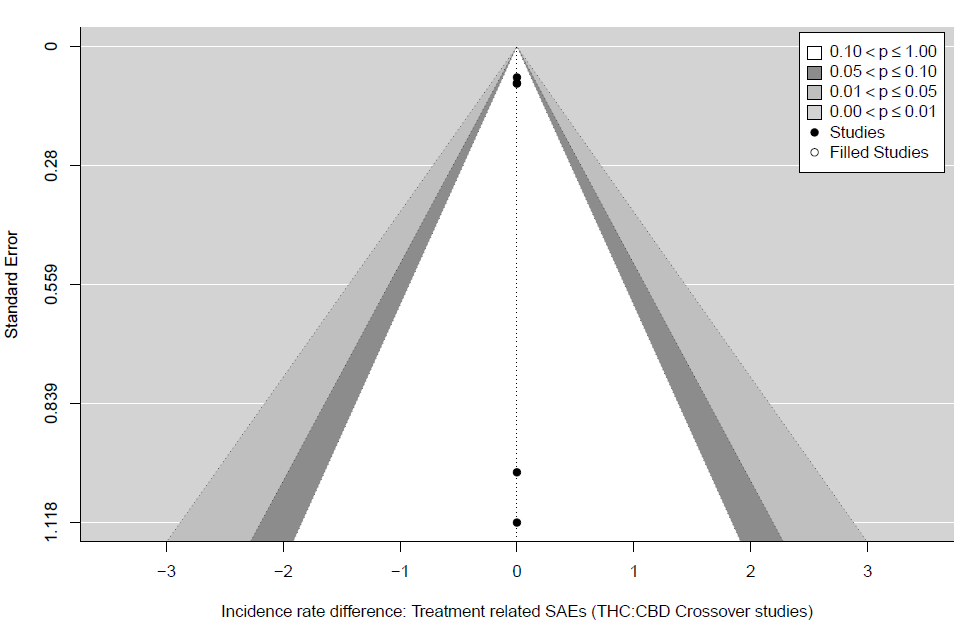


### Figure 22f. Funnel plots for treatment-related serious adverse events (SAEs) meta-analyses for THC:CBD RCT studies


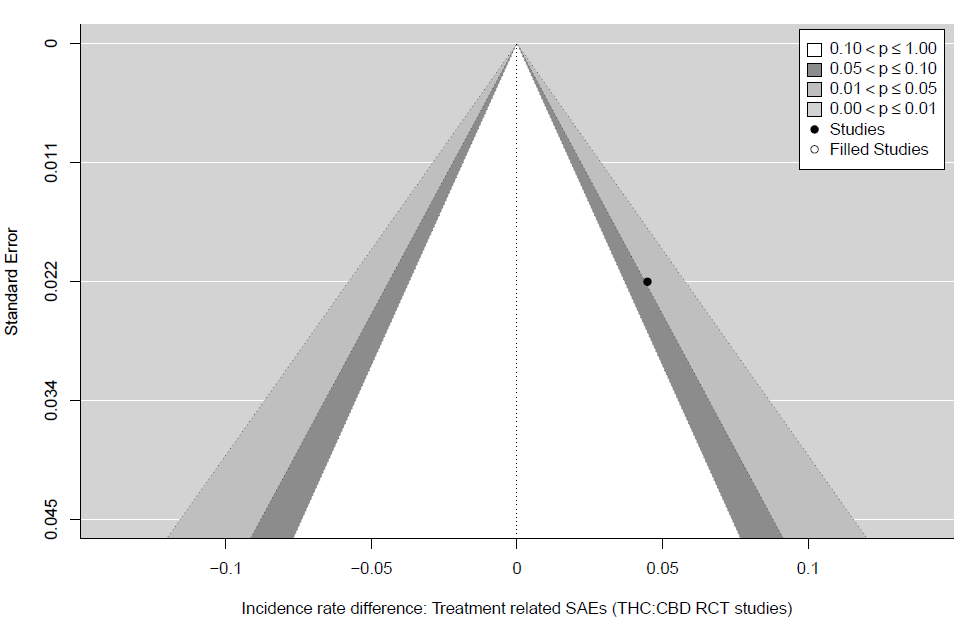


### Figure 23a. Funnel plots for all-cause withdrawals meta-analyses for THC:CBD studies


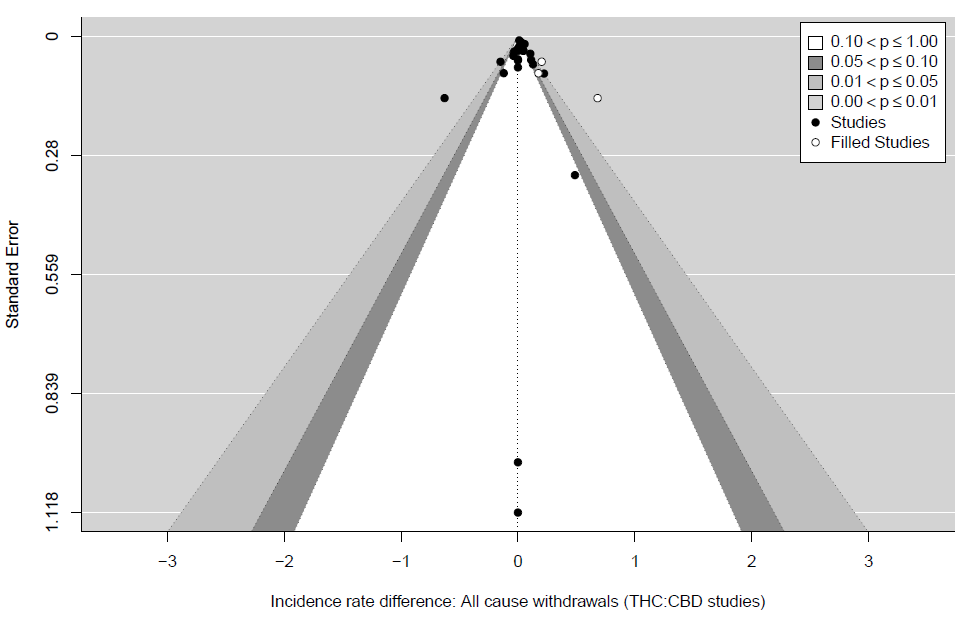


### Figure 23b. Funnel plots for all-cause withdrawals meta-analyses for THC:CBD RCT studies


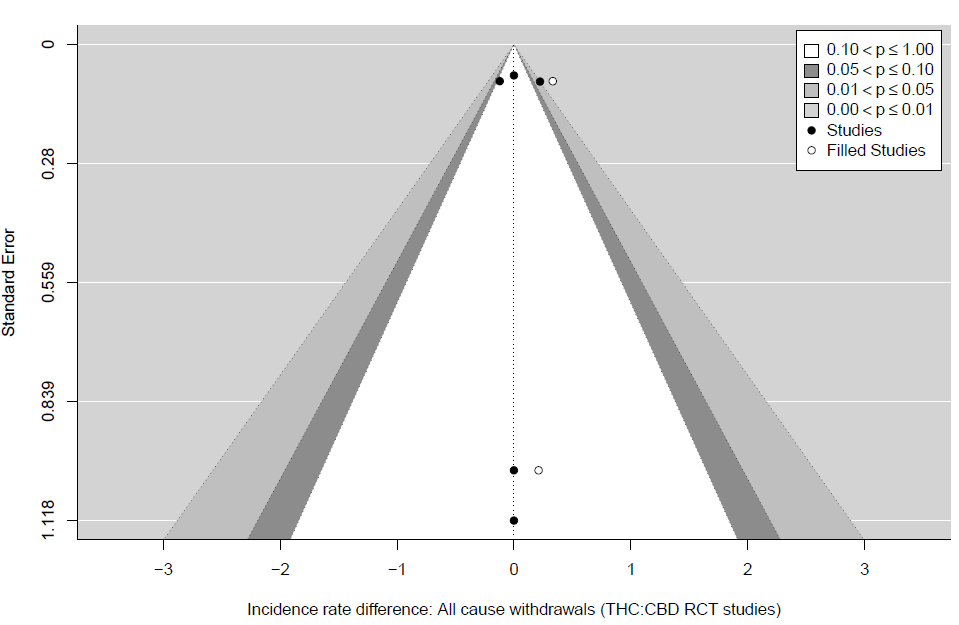


### Figure 23c. Funnel plots for treatment-related withdrawals meta-analyses for THC:CBD studies


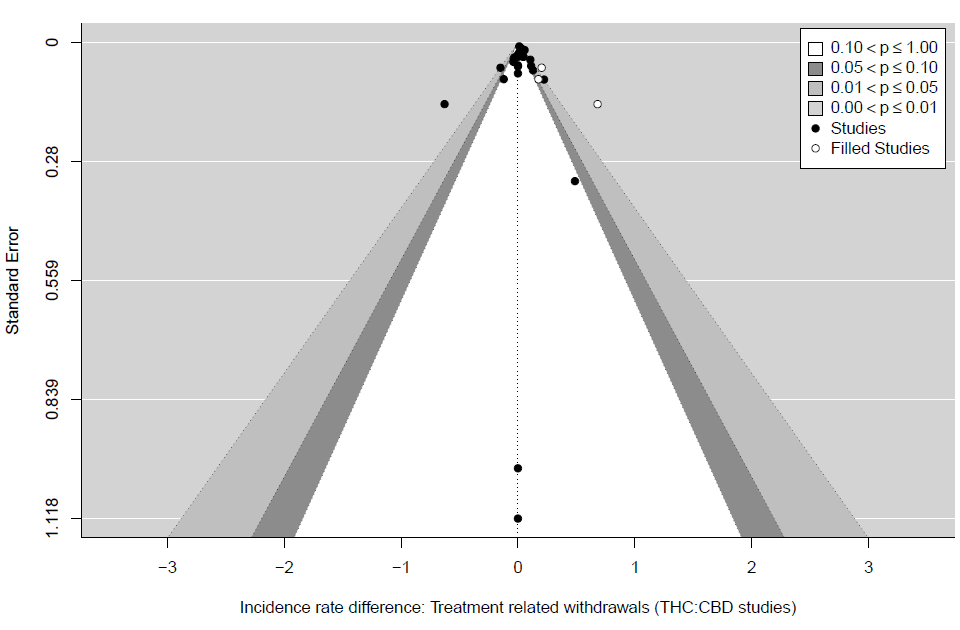


### Figure 23d. Funnel plots for treatment-related withdrawals meta-analyses for THC:CBD crossover studies


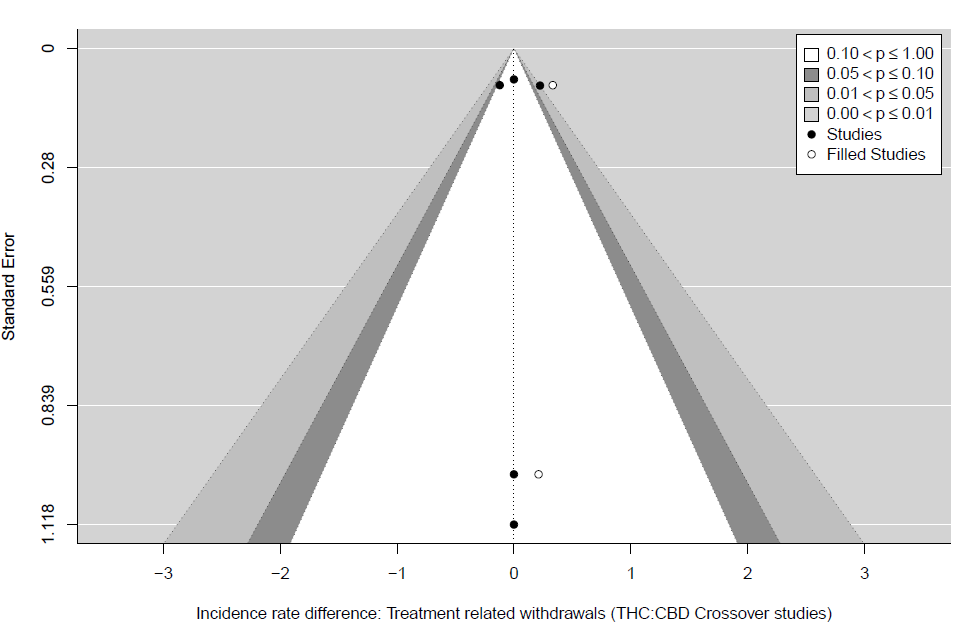


### Figure 23e. Funnel plots for treatment-related withdrawals meta-analyses for THC:CBD RCT studies


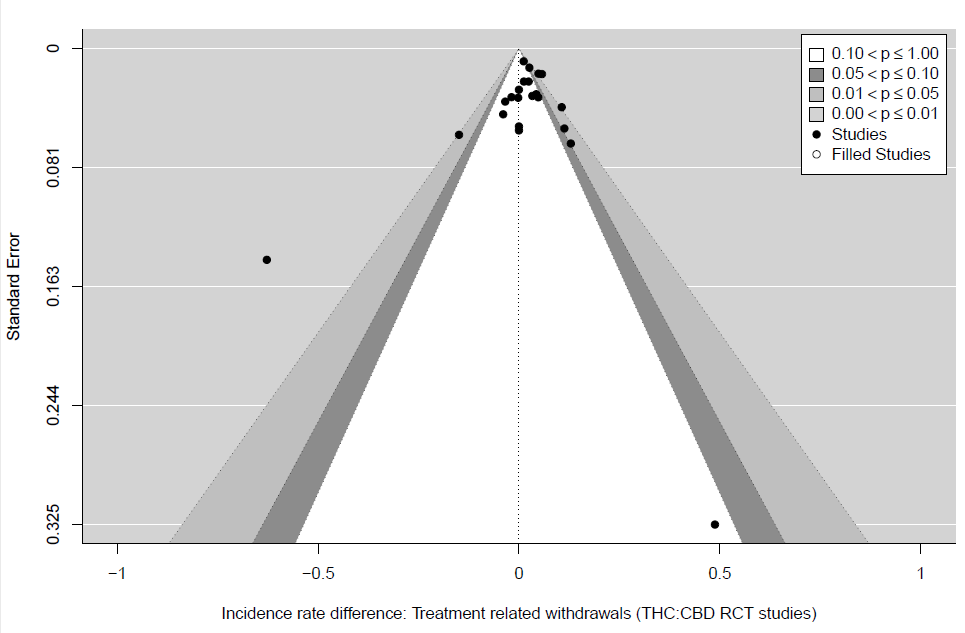


### Figure 24a. Funnel plots for deaths meta-analyses for THC:CBD studies


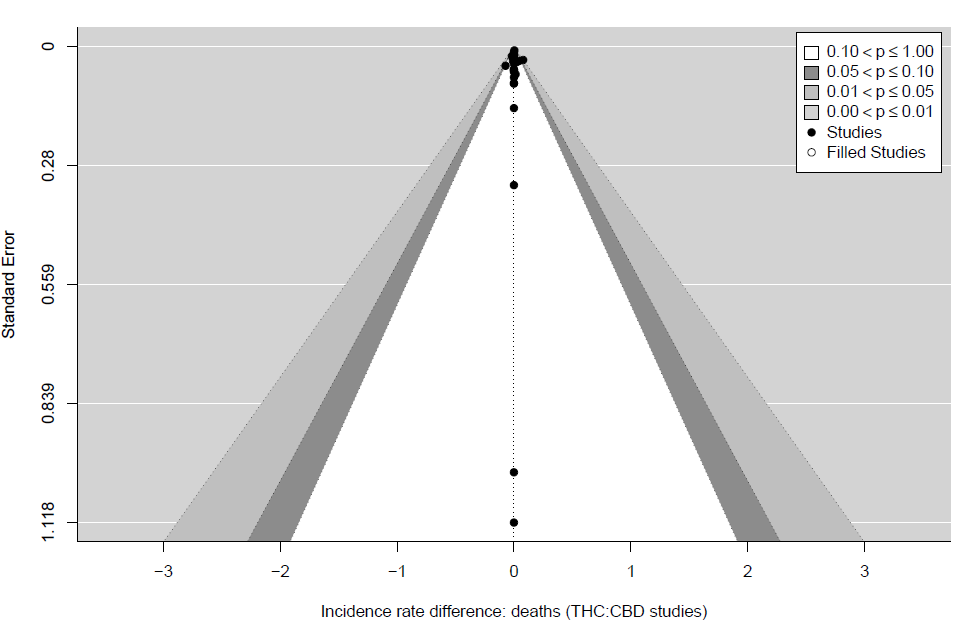


### Figure 24b. Funnel plots for deaths meta-analyses for THC:CBD crossover studies


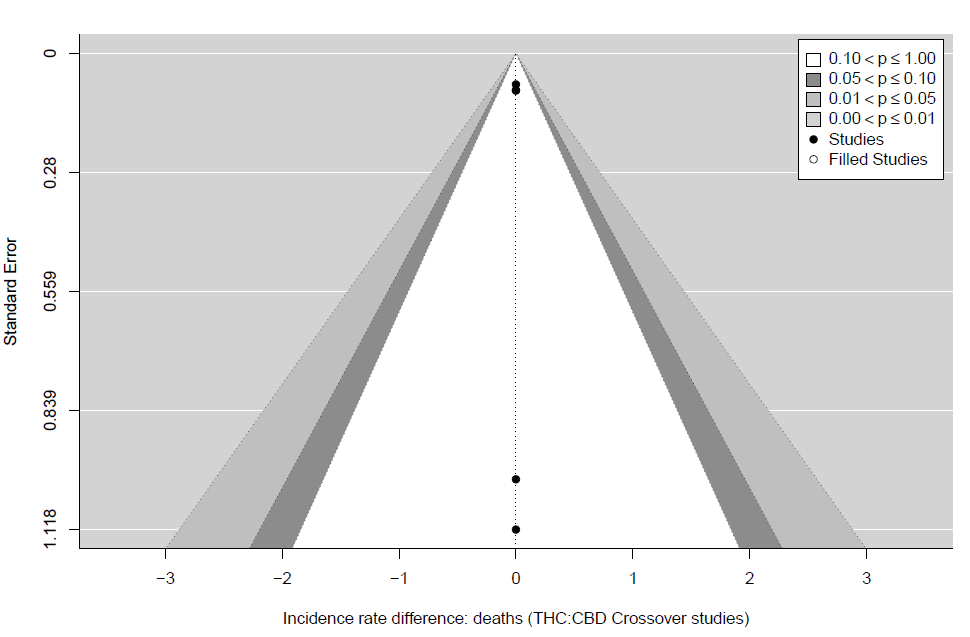


### Figure 24c. Funnel plots for deaths meta-analyses for THC:CBD RCT studies


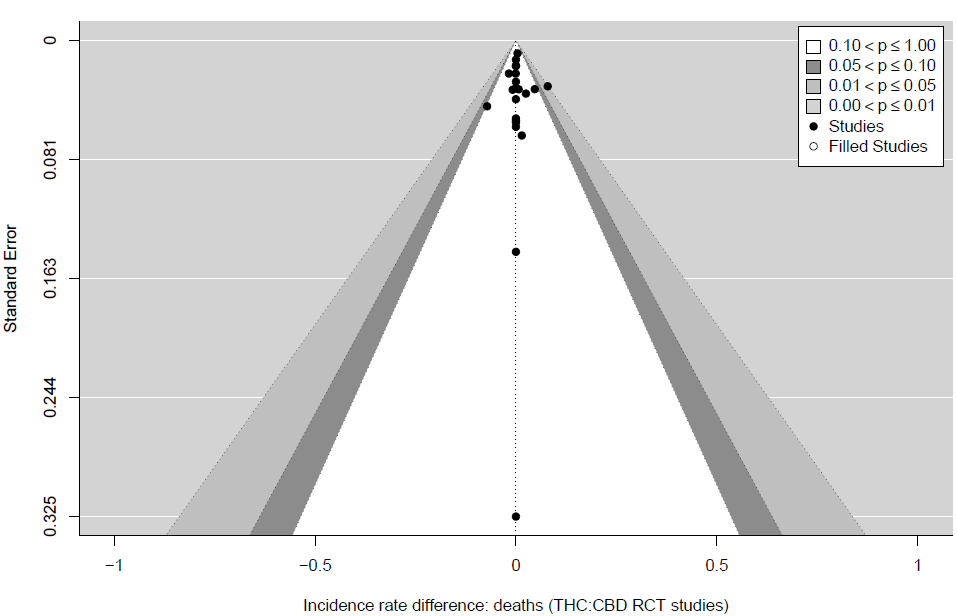


# TABLES

## Table 1a: Characteristics of included randomised controlled trials of THC in middle aged and older adults (N=31)

| Study ID (country) | Study Design  (RCT) | THC:  Sample included/analysed N  Mean age (SD), Male % | Comparator:  Sample included/  analysed N  Mean age (SD), Male % | Indication | THC classification | Comparator | THC treatment duration, weeks | Calculated daily average THC dose | Overall GRADE rating for study |
| --- | --- | --- | --- | --- | --- | --- | --- | --- | --- |
| Ahmed et al. 2014, (Netherlands) | Crossover | 12/11  72.00 (5), 50 | 12/11  72.00 (5), 50 | Healthy older subjects | Namisol | Placebo | 0.4ʇ | 6.5mg | Moderate |
| Ahmed et al. 2015  (Netherlands) | Crossover | 10/10  77.30 (5.6), 70 | 10/10  77.30 (5.6), 70 | Dementia | Namisol | Placebo | 2.6 | 3 mg | Moderate |
| Brisbois et al. 2011  (Canada) | Parallel-arm | 24/11  67.00 (10.9), 64 | 22/10  65.50 (8), 50 | Cancer patients with chemosensory alterations | Dronabinol | Placebo | 2.6 | 7.5 mg | Low |
| Carley et al. 2018  (USA) ^‡^ | Parallel-arm | 21/21  52.70 (7.7), 76 | 25/25  58.80 (6.1), 72 | Obstructive Sleep Apnoea | Dronabinol | Placebo | 6.0 | 2.5 mg | Low |
| Carley et al. 2018  (USA) ^‡^ | Parallel-arm | 27/27  54.70 (7), 67 | 25/25  58.80 (6.1), 72 | Obstructive Sleep Apnoea | Dronabinol | Placebo | 6.0 | 10 mg | Low |
| Curtis et al. 2009  (UK) | Crossover | 44/37  52.00 (9.5), 50 | 44/37  52.00 (9.5), 50 | Huntington’s disease | Nabilone | Placebo | 5.0 | 2 mg | Low |
| De Vries et al. 2016  (Netherlands) | Crossover | 25/24  52.00 (NR), 62 | 25/24  52.00 (NR), 62 | Chronic pancreatitis | Namisol | Diazepam | 0.1 ʇ | 8 mg | Moderate |
| Herrmann et al. 2019, (Canada) | Crossover | 39/38  87.00 (10), 77 | 39/38  87.00 (10), 77 | Alzheimer’s disease | Nabilone | Placebo | 6.0 | 1.6 mg | Moderate |
| Jadoon et al. 2016, UK | Parallel-arm | 12/12  63.00 (12.6), 83 | 14/14  59.00 (7.7), 50 | Diabetes (type 2) | THCV | Placebo | 13.0 | 10 mg | Moderate |
| Jatoi et al. 2002  (USA) | Parallel-arm | 152/152  67.00 (10), 66 | 159/159  65.00 (11), 65 | Cancer-related anorexia | Dronabinol | Megestrol acetate | 8.1 | 5 mg | Low |
| Johnson et al. 2010  (UK) ^§^ | Parallel-arm | 58/58  61.30 (12.5), 52 | 59/59  60.10 (12.3), 54 | Patients with cancer-related pain | THC extract spray | Placebo | 2.0 | 23 mg | Moderate |
| Lane et al. 1991  (USA) | Parallel-arm | 21/21  52.0 (20-68) ^*,†^  48 | 21/21  52.0 (22-64) ^*,†^  48 | Chemotherapy-induced nausea and vomiting | Dronabinol | Prochlorperazine | 0.9 ʇ | 40 mg | Low |
| Meiri et al. 2007  (USA) | Parallel-arm | 17/17  61.60 (14.2), 53 | 14/14  57.20 (8.6), 38 | Chemotherapy-induced nausea and vomiting | Dronabinol | Placebo | 0.7 ʇ | 20 mg | Low |
| Peball et al. 2020  (Austria) | Parallel-arm | 19/19  65.4 (7.94), 53 | 19/19  64.0 (8.04), 74 | Parkinson’s disease | Nabilone | Placebo | 4.0 | 0.75 mg | Moderate |
| Sieradzan et al. 2001(UK) | Crossover | 9/9  59.00 (NR), 44 | 9/9  59.00 (NR), 44 | Parkinson’s disease | Nabilone | Placebo | 0.1 ʇ | 2 mg | Very low |
| Strasser et al. 2006  (Germany) ^§^ | Parallel-arm | 100/100  60.00 (12), 54 | 48/48  62.00 (10), 52 | Cancer-related anorexia | THC | Placebo | 6.0 | 5 mg | Low |
| Svendsen et al. 2004  (Denmark) | Crossover | 24/24  50.0 (NR), 42 | 24/24  50.0 (NR), 42 | Multiple sclerosis | Dronabinol | Placebo | 3.0 | 10 mg | Moderate |
| Tomida et al. 2006  (UK) ^§^ | Crossover | 6/6  55.30 (5), 100 | 6/6  55.30 (5), 100 | Intraocular pressure | THC extract spray | Placebo | 0.1 ʇ | 5 mg | Low |
| Toth et al. 2012  (Canada) | Parallel-arm | 13/13  60.80 (15.3), 38 | 13/13  61.60 (14.6), 69 | Diabetic peripheral neuropathic pain | Nabilone | Placebo | 5.0 | 4 mg | Low |
| Van Amerongen et al. 2017, 2  (Netherlands) ^\|\|^ | Crossover | 24/24  54.30 (8.9), 33 | 24/24  54.30 (8.9), 33 | Multiple sclerosis | THC | Placebo | 0.1 ʇ | 16 mg | Moderate |
| Van Amerongen et al. 2017, 1  (Netherlands) ^\|\|^ | Parallel-arm | 12/12  57.30 (9), 33 | 12/12  51.40 (8), 33 | Multiple sclerosis | THC | Placebo | 4.0 | 28.5 mg | Moderate |
| Van den Elsen et al. 2015, 1  (Netherlands) | Parallel-arm | 24/24  79.00 (8), 46 | 26/26  78.00 (7), 54 | Dementia | Namisol | Placebo | 3.0 | 4.5 mg | Moderate |
| Van den Elsen et al. 2015, 2  (Netherlands) | Crossover | 22/22  76.40 (5.3), 68 | 22/22  76.40 (5.3), 68 | Dementia | Namisol | Placebo | 2.6 | 3 mg | Moderate |
| Volicer et al. 1997  (USA) | Crossover | 15/12  72.70 (4.9), 92 | 15/12  72.70 (4.9), 92 | Alzheimer’s disease | Dronabinol | Placebo | 6.0 | 5 mg | Very low |
| Walther et al. 2011  (Switzerland) | Crossover | 2/2  78.00 (NR), 100 | 2/2  78.00 (NR), 100 | Alzheimer’s disease | Dronabinol | Placebo | 2.0 | 2.5 mg | Very low |
| Ware et al. 2010  (Canada) | Crossover | 32/32  50.00 (11.2), 16 | 32/32  50.00 (11.2), 16 | Fibromyalgia | Nabilone | Amitriptyline | 2.0 | 1 mg | Moderate |
| Weber et al. 2010  (Switzerland) | Crossover | 27/22  57.00 (12), 74 | 27/22  57.00 (12), 74 | Amyotrophic lateral sclerosis patients with cramps | Dronabinol | Placebo | 2.0 | 10 mg | Moderate |
| Zadikoff et al. 2011  (Canada) | Crossover | 9/9  60.00 (7), 0 | 9/9  60.00 (7), 0 | Cervical dystonia | Dronabinol | Placebo | 3.0 | 15 mg | Low |
| Zajicek et al. 2003  (UK) ^§^ | Parallel-arm | 216/206  50.00 (8.2), 31 | 222/213  51.00 (7.6), 37 | Multiple sclerosis | Dronabinol | Placebo | 14.0 | 25mg | Moderate |
| Zajicek et al. 2005  (UK) ^§^ | Parallel-arm | 125/125  50.00 (8.2), 31 | 120/120  51.00 (7.6), 37 | Multiple sclerosis | Dronabinol | Placebo | 52.0 | 25 mg | Moderate |
| Zajicek et al. 2013  (UK) | Parallel-arm | 332/329  52.30 (7.6), 40 | 166/164  52.00 (8.2), 41 | Multiple sclerosis | Dronabinol | Placebo | 160.0 | 28 mg | Moderate |

*, Median age (range); †, Included as median age for whole study population was ≥50; ‡^,^ Article included more than one dose level; ʇ Treatment duration less than a week; §, Article included more than one cannabinoid intervention; ^||^ , Article included the results of multiple trials; ^¶^, Article included multiple study groups/indications ; NR, Not recorded.

## Table 1b: Characteristics of included randomised controlled trials of CBD:THC in middle aged and older adults (N=27).

| Study ID (country) | Study Design | CBD/THC:  Sample included/  analysed N  Mean age (SD), Male % | Comparator:  Sample included/  analysed N  Mean age (SD), Male % | Indication | CBD/THC classification | Comparator | CBD/THC treatment duration, weeks | Calculated daily average CBD/THC dose | GRADE rating |
| --- | --- | --- | --- | --- | --- | --- | --- | --- | --- |
| Blake et al. 2006  (UK) | Parallel-arm | 31/31  60.9 (10.6), 26 | 27/27  64.9 (8.5), 15 | Rheumatoid arthritis | THC:CBD spray | Placebo | 5.0 | 14.6mg THC: 13.5mg CBD | Low |
| Carroll et al. 2004  (UK) | Crossover | 19/17  67.0 (NR)  63 | 19/17  67.0 (NR)  63 | Levodopa induced dyskinesia in Parkinson’s disease | Cannabis extract | Placebo | 4.0 | 10.2mg THC: 5.1mg CBD | Moderate |
| Duran et al. 2010  (Spain) | Parallel-arm | 7/7  50 (41-70) *  0 | 9/9  50 (34-76) *  11 | Chemotherapy induced nausea and vomiting | THC:CBD spray | Placebo | 0.6 ʇ | 13mg THC : 12mg CBD | Moderate |
| Fallon et al. 2017, 1  (Multicentre) ^\|\|^ | Parallel-arm (withdrawal study) | 103/103  61.4 (10.9), 61 | 103103  61.6 (11.8), 53 | Advanced cancer patients with pain | THC:CBD spray | Placebo | 5.0 | 17.6mg THC: 16.3mg CBD | Moderate |
| Fallon et al. 2017, 2  (Multicentre) ^\|\|^ | Parallel-arm | 200/199  60.0 (11), 53 | 199/198  59.6 (11), 49 | Advanced cancer patients with pain | THC:CBD spray | Placebo | 5.0 | 17mg THC: 15.8mg CBD | Moderate |
| Jadoon et al. 2016, 1  (UK) ^‡,§^ | Parallel-arm | 11/11  59.0 (8.8), 55 | 14/14  59.0 (7.7), 50 | Type 2 diabetes | CBD/THCV | Placebo | 13.0 | 10mg THC: 10mg CBD | Moderate |
| Jadoon et al. 2016, 2  (UK) ^‡,§^ | Parallel-arm | 12/12  58.0 (8.1), 75 | 14/14  59.0 (7.7), 50 | Type 2 diabetes | CBD/THCV | Placebo | 13.0 | 10mg THC: 200mg CBD | Moderate |
| Johnson et al. 2010, (UK) ^§^ | Parallel-arm | 60/60  59.4 (12.1), 55 | 59/59  60.1 (12.3), 54 | Patients with cancer-related pain | THC:CBD spray | Placebo | 2.0 | 25mg THC: 23mg CBD | Moderate |
| Litchman et al. 2018, (Multicentre) | Parallel-arm | 199/199  59.2 (12), 56 | 198/198  60.7 (11.1), 52 | Advanced cancer patients with pain | THC:CBD spray | Placebo | 5.0 | 17.3mg THC: 16mg CBD | Moderate |
| Lynch et al. 2014  (USA) | Crossover | 18/16  56.0 (10.8), 17 | 18/16  56.0 (10.8), 17 | Chemotherapy-induced neuropathic pain | THC:CBD spray | Placebo | 6.0 | 21.6mg THC: 20mg CBD | Low |
| Markova et al. 2019, (Czech Republic) | Parallel-arm | 53/53  51.3 (10.2)  30 | 53/53  51.3 (10.2)  30 | Multiple sclerosis | THC:CBD spray | Placebo | 12.0 | 19.7mg THC: 18.3mg CBD | Low |
| Notcutt et al. 2012  (UK) | Parallel-arm (withdrawal study) | 18/18  59.7 (9)  50 | 18/18  54.4 (10.4)  33 | Multiple sclerosis | THC:CBD spray | Placebo | 4.0 | 20.8mg THC: 19.3mg CBD | Very low |
| Nurmikko et al. 2007, (UK) | Parallel-arm | 63/63  52.4 (15.8), 44 | 62/62  54.3 (15.2), 37 | Neuropathic pain | THC:CBD spray | Placebo | 5.0 | THC 29.7mg: CBD 27.5mg | High |
| ***Pickering et al. 2011, 1  (UK) ^¶^ | Crossover | 5/4  67.0 (NR), 50 | 5/4  67.0 (NR), 50 | COPD | THC:CBD spray | Placebo | 0.1 ʇ | 4.7mg THC: 4.4mg CBD | Low |
| Pickering et al. 2011, 2  (UK) ^¶^ | Crossover | 6/5  58.0 (NR), 80 | 6/5  58.0 (NR), 80 | Healthy controls | THC:CBD spray | Placebo | 0.1 ʇ | 10.3mg THC: 9.5mg CBD | Low |
| Portenoy et al. 2012, 1  (Multicentre) ^‡^ | Parallel-arm | 91/91  59.0 (12.3), 49 | 91/91  56.0 (12.2), 48 | Cancer patients with chronic pain | THC:CBD spray | Placebo | 5.0 | 10.8mg THC: 10mg CBD | Moderate |
| Portenoy et al. 2012, 2  (Multicentre) ^‡^ | Parallel-arm | 88/87  59.0 (13.1), 56 | 91/91  56.0 (12.2), 48 | Cancer patients with chronic pain | THC:CBD spray | Placebo | 5.0 | 27mgTHC: 25mg CBD | Moderate |
| Portenoy et al. 2012, 3  (Multicentre) ^‡^ | Parallel-arm | 90/90  58.0 (11.2), 53 | 91/91  56.0 (12.2), 48 | Cancer patients with chronic pain | THC:CBD spray | Placebo | 5.0 | 43.2mg THC: 40mg CBD | Moderate |
| Riva et al. 2019  (Italy) | Parallel-arm | 30/29  58.4 (10.6)  62 | 30/30  57.2 (13.8)  53 | Motor neurone disease | THC:CBD spray | Placebo | 6.0 | 21.6mg THC: 20.0mg CBD | High |
| Serpell et al. 2014, (UK) | Parallel-arm | 128/128  57.6 (14.4), 34 | 118/118  57.0 (14.1), 45 | Neuropathic pain | THC:CBD spray | Placebo | 14.0 | 24mg THC: 22mg CBD | Moderate |
| Strasser et al. 2006  (Germany) ^§^ | Parallel-arm | 95/95  61.0 (12), 56 | 48/48  62.0 (10), 52 | Cancer-related anorexia | Cannabis extract | Placebo | 6.0 | 5mg THC: 2mg CBD | Moderate |
| Vaney et al. 2004  (Switzerland) | Crossover | 57/50  55.0 (10), 49 | 57/50  55.0 (10), 49 | Multiple sclerosis | Cannabis extract | Placebo | 2.0 | 27.5mg THC: 9.9mg CBD | Low |
| Wade et al. 2004  (UK) | Parallel-arm | 80/80  51.0 (9.4), 41 | 80/80  50.0 (9.3), 35 | Multiple sclerosis | THC:CBD spray | Placebo | 6.0 | 40.5mg THC: 37.5mg CBD | Moderate |
| Zajicek et al. 2003, (UK) ^§^ | Parallel-arm | 219/211  51.0 (7.6), 36 | 222/213  51.0 (7.6), 37 | Multiple sclerosis | Cannabis extract | Placebo | 14.0 | 25mg THC: 12.5mg CBD | Moderate |
| Zajicek et al. 2005, (UK) ^§^ | Parallel-arm | 138/138  51.0 (7.6), 36 | 120/120  51.0 (7.6), 37 | Multiple sclerosis | Cannabis extract | Placebo | 52.0 | 25mg THC: 12.5mg CBD | Moderate |
| Zajicek et al. 2012, (UK) | Parallel-arm | 144/143  51.9 (7.7), 39 | 135/134  52.0 (7.9), 35 | Multiple sclerosis | Cannabis extract | Placebo | 12.0 | 25mg THC: 12.5mg CBD | Moderate |
| Twelves et al. 2021, (UK, Germany) | Parallel-arm | 12/12 57.8 (10.7), 42 | 9/9 57.8 (8.29), 89 | Cancer | THC:CBD spray | Placebo | 25.0 | 20.25mg THC: 18.75mg CB | Moderate |

*, Median age (range); †, Included as median age for whole study population was ≥50; ‡^,^ Article included more than one dose level; ʇ Treatment duration less than a week; §, Article included more than one cannabinoid intervention; ^||^ , Article included the results of multiple trials; ^¶^, Article included multiple study groups/indications ; NR, Not recorded.

## Table 2a. Incidence rate difference for each system of classification (THC studies)

Effect of cumulative THC treatment across studies expressed as incidence rate difference (IRD, indicated by the summary estimate followed by 95% confidence intervals and associated *p* value) for each system of classification.

| MedDRA high-level grouping | Summary estimate | 95% CI (lower, upper) | P value | k | QE | QEp | I2 |
| --- | --- | --- | --- | --- | --- | --- | --- |
| Gastrointestinal | **1.2589** | **0.1370, 3.5114** | **0,003** | **60** | **93.625** | **0.003** | NA |
| General | 0.0004 | 0.0233, 0.0371 | 0.819 | 54 | 11.805 | 1.000 | NA |
| Cardiac | 0.0004 | 0.0573, 0.0778 | 0.881 | 39 | 7.754 | 1.000 | NA |
| Nervous System | **0.8588** | **0.3106, 4.7064** | **<0.001** | **75** | **147.988** | **<0.001** | NA |
| Psychiatric | **0.4402** | **0.0971, 4.3620** | **0.008** | **86** | **116.445** | **0.013** | NA |
| Skin/Subcutaneous | 0.0332 | 0.1956, 0.0060 | 0.170 | 45 | 8.147 | 1.000 | NA |
| Metabolism | 0.0001 | 0.0588, 0.0690 | 0.938 | 48 | 9.566 | 1.000 | NA |
| Infections | 0.0030 | 0.0377, 0.0072 | 0.443 | 48 | 17.480 | 1.000 | NA |
| Musculoskeletal | 0.0068 | 0.0409, 0.0014 | 0.178 | 51 | 17.780 | 1.000 | NA |

* Medical Dictionary for Regulatory Activities (MedDRA).

I^2^= percent of total variability (heterogeneity plus sampling variability) attributed to heterogeneity among the true effects. N= number of studies included in analysis. QE= test statistic for the test of heterogeneity. QEp= p value for the test of heterogeneity. NA = not applicable

## Table 2b. Incidence rate difference for each system of classification (THC:CBD studies)

Effect of cumulative THC:CBD combination treatment across THC:CBD studies expressed as incidence rate difference (IRD, indicated by the summary estimate, followed by 95% confidence intervals and associated *p* value) for each system of classification.

| MedDRA high-level grouping | Summary estimate | 95% CI (lower, upper) | P value | k | QE | QEp | I2 |
| --- | --- | --- | --- | --- | --- | --- | --- |
| Gastrointestinal | **0.6877** | **0.2314, 1.3864** | **<0.001** | **57** | **101.314** | **0.000** | NA |
| General | **0.0966** | **0.0012, 0.3446** | **0.027** | 47 | 58.398 | 0.104 | NA |
| Cardiac | 0.0001 | 0.0540, 0.0647 | 0.929 | 30 | 14.345 | 0.989 | NA |
| Nervous System | **0.7046** | **0.2028, 1.5091** | **<0.001** | **74** | **177.124** | **<0.001** | NA |
| Psychiatric | **0.5610** | **0.1553, 1.2186** | **<0.001** | **80** | **157.206** | **<0.001** | NA |
| Skin/Subcutaneous | 0.0154 | 0.1365, 0.0147 | 0.322 | 28 | 7.245 | 1.000 | NA |
| Metabolism | 0.0168 | 0.0088, 0.1247 | 0.255 | 40 | 29.717 | 0.858 | NA |
| Infections | 0.0030 | 0.0377, 0.0072 | 0.443 | 30 | 3.440 | 1.000 | NA |
| Musculoskeletal | 0.0068 | 0.0409, 0.0014 | 0.177 | 29 | 6.771 | 1.000 | NA |

* Medical Dictionary for Regulatory Activities (MedDRA).

I^2^= percent of total variability (heterogeneity plus sampling variability) attributed to heterogeneity among the true effects. N= number of studies included in analysis. QE= test statistic for the test of heterogeneity. QEp= p value for the test of heterogeneity. NA = not applicable

# REFERENCES

1. Velayudhan L, McGoohan K, Bhattacharyya S. Safety and tolerability of natural and synthetic cannabinoids in adults aged over 50 years: A systematic review and meta-analysis. PLoS Med. 2021;18(3):e1003524. Epub 2021/03/30. doi: 10.1371/journal.pmed.1003524. PubMed PMID: 33780450; PubMed Central PMCID: PMCPMC8007034.

2. Twelves C, Sabel M, Checketts D, Miller S, Tayo B, Jove M, et al. A phase 1b randomised, placebo-controlled trial of nabiximols cannabinoid oromucosal spray with temozolomide in patients with recurrent glioblastoma. British journal of cancer. 2021;124(8):1379-87. Epub 2021/02/25. doi: 10.1038/s41416-021-01259-3. PubMed PMID: 33623076; PubMed Central PMCID: PMCPMC8039032 M.J., L.B. and S.S. have no conflicts of interest to declare.

3. Guyatt GH, Oxman AD, Vist GE, Kunz R, Falck-Ytter Y, Alonso-Coello P, et al. GRADE: an emerging consensus on rating quality of evidence and strength of recommendations. BMJ (Clinical research ed). 2008;336(7650):924-6. Epub 2008/04/26. doi: 10.1136/bmj.39489.470347.AD. PubMed PMID: 18436948; PubMed Central PMCID: PMCPMC2335261 GRADE’s success has a positive influence on their academic career. Authors listed in the byline have received travel reimbursement and honorariums for presentations that included a review of GRADE’s approach to rating quality of evidence and grading recommendations. GHG acts as a consultant to UpToDate; his work includes helping UpToDate in their use of GRADE. HJS is documents editor and methodologist for the American Thoracic Society; one of his roles in these positions is helping implement the use of GRADE. He is supported by “The human factor, mobility and Marie Curie actions scientist reintegration European Commission grant: IGR 42192—GRADE.”.

4. Balshem H, Helfand M, Schünemann HJ, Oxman AD, Kunz R, Brozek J, et al. GRADE guidelines: 3. Rating the quality of evidence. Journal of clinical epidemiology. 2011;64(4):401-6. Epub 2011/01/07. doi: 10.1016/j.jclinepi.2010.07.015. PubMed PMID: 21208779.

5. Bosnjak Kuharic D, Markovic D, Brkovic T, Jeric Kegalj M, Rubic Z, Vuica Vukasovic A, et al. Cannabinoids for the treatment of dementia. The Cochrane database of systematic reviews. 2021;9(9):Cd012820. Epub 2021/09/18. doi: 10.1002/14651858.CD012820.pub2. PubMed PMID: 34532852; PubMed Central PMCID: PMCPMC8446835.

6. Torres-Moreno MC, Papaseit E, Torrens M, Farré M. Assessment of Efficacy and Tolerability of Medicinal Cannabinoids in Patients With Multiple Sclerosis: A Systematic Review and Meta-analysis. JAMA network open. 2018;1(6):e183485. Epub 2019/01/16. doi: 10.1001/jamanetworkopen.2018.3485. PubMed PMID: 30646241; PubMed Central PMCID: PMCPMC6324456.

7. Black N, Stockings E, Campbell G, Tran LT, Zagic D, Hall WD, et al. Cannabinoids for the treatment of mental disorders and symptoms of mental disorders: a systematic review and meta-analysis. The lancet Psychiatry. 2019;6(12):995-1010. Epub 2019/11/02. doi: 10.1016/s2215-0366(19)30401-8. PubMed PMID: 31672337; PubMed Central PMCID: PMCPMC6949116.

8. Wang L, Hong PJ, May C, Rehman Y, Oparin Y, Hong CJ, et al. Medical cannabis or cannabinoids for chronic non-cancer and cancer related pain: a systematic review and meta-analysis of randomised clinical trials. BMJ (Clinical research ed). 2021;374:n1034. Epub 2021/09/10. doi: 10.1136/bmj.n1034. PubMed PMID: 34497047.

9. AminiLari M, Wang L, Neumark S, Adli T, Couban RJ, Giangregorio A, et al. Medical cannabis and cannabinoids for impaired sleep: a systematic review and meta-analysis of randomized clinical trials. Sleep. 2022;45(2). Epub 2021/09/22. doi: 10.1093/sleep/zsab234. PubMed PMID: 34546363.
